# Glycopolymer-Functionalized MOF-808 Nanoparticles as a Cancer-Targeted Dual Drug Delivery System for Carboplatin and Floxuridine

**DOI:** 10.1021/acsanm.2c01632

**Published:** 2022-06-22

**Authors:** Fatma Demir Duman, Alessandra Monaco, Rachel Foulkes, C. Remzi Becer, Ross S. Forgan

**Affiliations:** † WestCHEM, School of Chemistry, University of Glasgow, University Avenue, Glasgow G12 8QQ, U.K.; ‡ Department of Chemistry, University of Warwick, CV4 7AL Coventry, U.K.

**Keywords:** metal−organic framework, glycopolymer, drug delivery, cancer, synergistic, targeting, carbohydrates

## Abstract

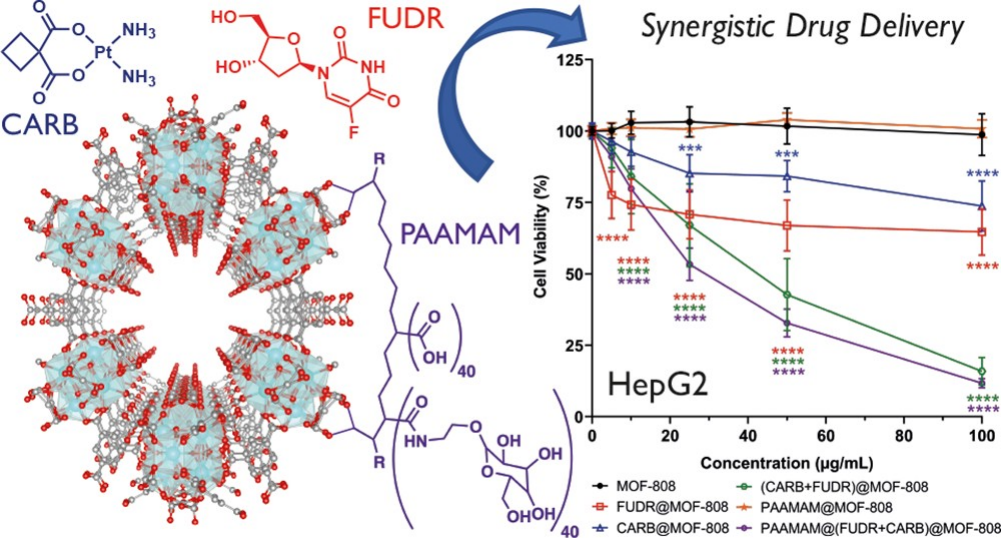

Codelivery of chemotherapeutics via nanomaterials has attracted much attention over the last decades due to improved drug delivery to tumor tissues, decreased systemic effects, and increased therapeutic efficacies. High porosities, large pore volumes and surface areas, and tunable structures have positioned metal–organic frameworks (MOFs) as promising drug delivery systems (DDSs). In particular, nanoscale Zr-linked MOFs such as MOF-808 offer notable advantages for biomedical applications such as high porosity, good stability, and biocompatibility. In this study, we report efficient dual drug delivery of floxuridine (FUDR) and carboplatin (CARB) loaded in MOF-808 nanoparticles to cancer cells. The nanoparticles were further functionalized by a poly(acrylic acid-mannose acrylamide) (PAAMAM) glycopolymer coating to obtain a highly selective DDS in cancer cells and enhance the therapeutic efficacy of chemotherapy. While MOF-808 was found to enhance the individual therapeutic effects of FUDR and CARB toward cancerous cells, combining FUDR and CARB was seen to cause a synergistic effect, further enhancing the cytotoxicity of the free drugs. Enhancement of CARB loading and therefore cytotoxicity of the CARB-loaded MOFs could be induced through a modified activation protocol, while coating of MOF-808 with the PAAMAM glycopolymer increased the uptake of the nanoparticles in cancer cells used in the study and offered a particularly significant selective drug delivery with high cytotoxicity in HepG2 human hepatocellular carcinoma cells. These results show how the enhancement of cytotoxicity is possible through both nanovector delivery and synergistic treatment, and that MOF-808 is a viable candidate for future drug delivery studies.

## Introduction

Cancer is a leading cause of death worldwide due to its inherent complexities.^1^ Genetic alterations, cellular abnormalities, and heterogeneity can cause aggressive growth, making the cancer less susceptible to conventional therapies.^2^ Currently, chemotherapy is the most commonly used technique to treat cancer because of its efficiency. However, conventional systemic single-drug systems suffer from several limitations, including limited accessibility to tumor tissues, rapid blood/renal clearance, nonspecific selectivity and adverse side effects.^3^ Over the years, the combination therapy of multiple non-cross-resistant anticancer drugs has been adopted in clinics, as it offers several benefits, including delay of the associated tumor cell mutations and tumor adaptation process. Multiple drugs with different molecular targets can function synergistically by decreasing the drug doses, thereby reducing side effects while achieving higher therapeutic efficacy and target selectivity.^4,5^

Floxuridine (FUDR), a hydrophilic derivative of 5-fluorouracil (5-FU), is a very effective drug with high antitumor activity against various tumors such as colorectal, liver, and colon cancer.^6^ FUDR inhibits thymidylate synthase and thereby prevents DNA synthesis. This antimetabolite is metabolized to fluorouracil when administered and can disrupt RNA synthesis by preventing the utilization of preformed uracil and incorporating into RNA.^7^ Although FUDR is a clinically effective drug, its poor oral absorption, low tumor selectivity, and nonselective uptake at nontumor tissues cause systemic side effects.^7,8^ Platinum-based drugs are heavily used in chemotherapy regimens for the treatment of various cancers, including ovarian, lung, liver, breast, head and neck, brain, colon, and testicular.^9^ Carboplatin (CARB) is a second-generation platinum-based chemotherapeutic which has a similar mechanism of action to that of archetypal cisplatin but causes much less toxicity and has improved antitumor activity.^10^ CARB acts as an alkylating agent by covalently binding to DNA and induces apoptosis and necrosis by damaging DNA replication. However, drug resistance of cancer cells limits its clinical application. Combining CARB with different chemotherapy drugs has resulted in a higher therapeutic index in the treatment of advanced cancers such as in advanced ovarian cancer with CARB/paclitaxel,^11^ in advanced bladder carcinoma with CARB/gemcitabine,^12^ and in advanced nonsmall-cell lung cancer with CARB/paclitaxel/gemcitabine combination chemotherapy.^13^ However, CARB has very short biological half-life and, similar to FUDR, it suffers high systemic toxicity.^14^ These factors, combined with their existing approval for use in the clinic, make CARB and FUDR prime candidates for targeted drug delivery, to enhance therapeutic efficiency by using smaller dosages that are targeted to the disease locus to minimize off-target effects.

The great progress in nanotechnology has opened up unprecedented opportunities for innovative combination strategies and controlled drug delivery, with nanoparticle systems improving the delivery of cargo to the target tissues and therefore the therapeutic efficacy of drugs.^15−19^ Compared to conventional systemic treatment regimens using multiple free drugs in a synergistic manner, the encapsulation of different drugs within a single nanoscale drug delivery system can dramatically reduce systemic toxicity by suppressing the premature degradation and nonspecific interactions of drugs with normal tissues, improving drug solubility, and providing longer circulation times in the blood.^20−26^ Such a structure can reduce frequency of drug administration, ensure controllable release profiles, and increase the accumulation of drugs in tumor tissues through the enhanced permeability and retention (EPR) effect and hence enhance the therapeutic efficacy.^24,27^

Metal–organic frameworks (MOFs), consisting of metal ions or clusters linked by multitopic organic ligands, represent a new generation of synthetic, porous, hybrid macromolecular structures.^28^ Their wide structural variety, owing to the possible combinations of metal clusters and organic linkers, has brought them many features, such as large pore volumes and surface areas, tunable pore environment, and surface chemistry, which make them advantageous for a wide range of applications as well as in drug delivery and imaging.^29−43^ Their high porosity and tunable structures offer high drug payloads and biocompatibility, as well as enhanced stability and hybridization with numerous functionalities such as introduction of targeting moieties to the outer surface^44^ for targeted delivery applications.^45^ As the targeting of cancerous tissues or tumors can significantly enhance the efficacy of the treatment, this is also associated with a reduction in off-target effects. Carbohydrates play an important role in cell–cell signaling, adhesion, cell migration, and cancer development and metastasis.^46−48^ Glycopolymers, which are composed of a synthetic polymer backbone decorated with pendant sugar moieties, have attracted great interest due to high specificity of carbohydrates to the sugar binding proteins (lectins) which are overexpressed on the surface of cancer cells and that can be employed to target and deliver drugs toward cancer cells by receptor-mediated endocytosis.^49,50^ The interaction between carbohydrates and lectins has a key role in cancer growth and progression, which involves interaction of tumor cells with other tumor cells, endothelial cells, and the extracellular membrane.^48,51^ The binding between carbohydrates and lectins is usually weak, but it can be significantly enhanced by the “glycocluster effect”, whereby polyvalent carbohydrate ligands increase the binding constant.^48,52^ Glycopolymers therefore have great potential for binding to lectins and providing high uptake of drug delivery systems to cancer cells.^53−55^

**Figure 1:**
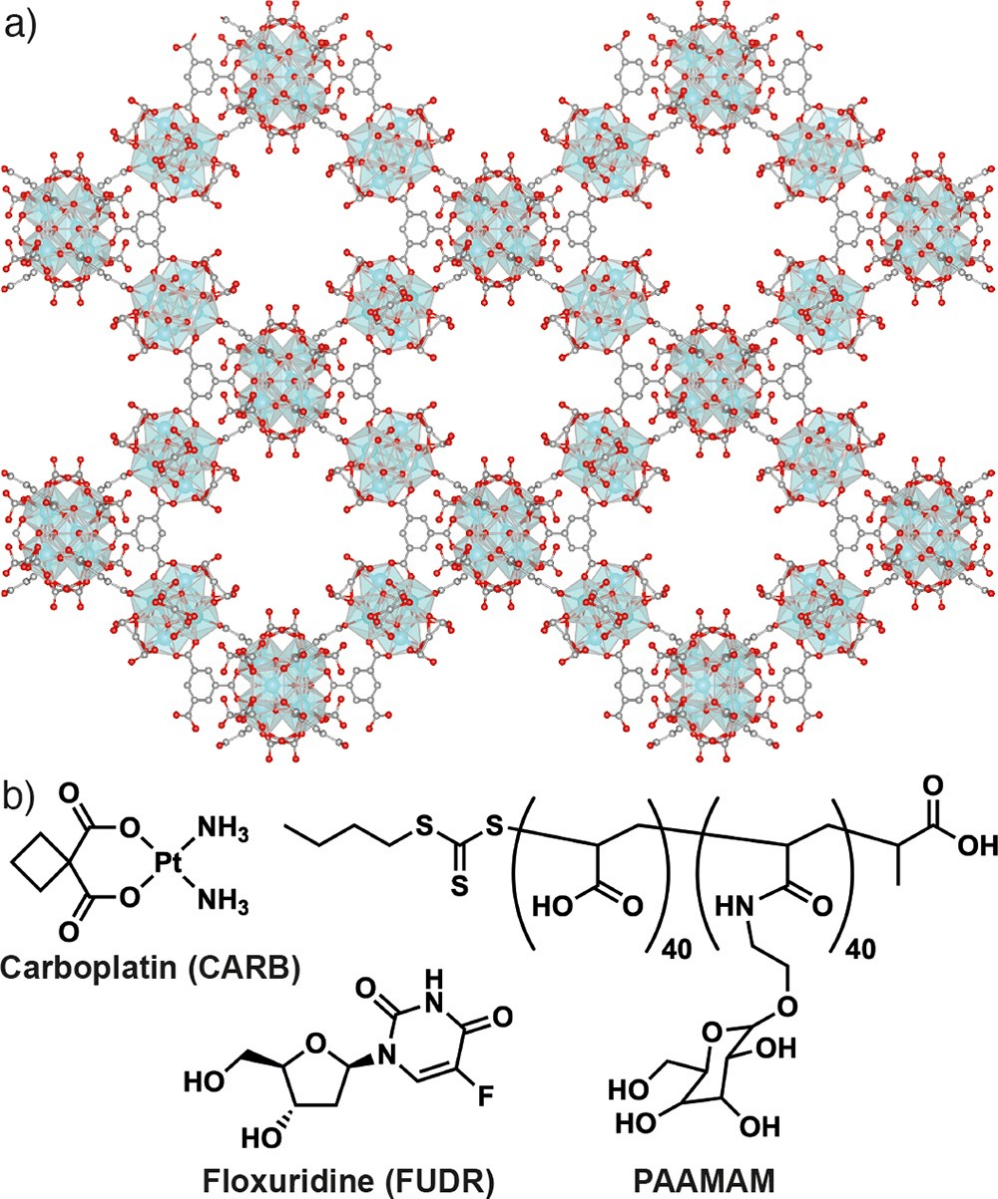
(a) Packing structure of MOF-808 showing its hexagonal pores of ∼1.8 nm diameter. (b) Chemical structures of the chemotherapeutic agents carboplatin (CARB) and floxuridine (FUDR) and the glycopolymer poly(acrylic acid-mannose acrylamide) (PAAMAM), used in this study.

For these reasons, we present here a facile synthesis of the Zr-MOF MOF-808 (Figure 1a),^56^ multifunctionalized through dual drug loading of FUDR and CARB, and poly(acrylic acid-mannose acrylamide) (PAAMAM) glycopolymer coating (Figure 1b), to enhance efficacy and reduce side-effects of chemotherapy by synergistic effects of the anticancer drugs and their higher accumulation in cancer cells by glycopolymer-mediated targeting. Comprehensive characterization has shown that ∼10% (*w*/*w*) loading of CARB and ∼1% (*w*/*w*) loading of FUDR in MOF-808 nanoparticles are easily achievable, with absolute values dependent on activation protocols. In vitro cytotoxicity studies performed in MCF-7 human breast adenocarcinoma, PANC-1 human pancreatic ductal adenocarcinoma, and HepG2 human hepatocellular carcinoma cells show that the cytotoxicity of the drugs is greatly enhanced through the use of MOF-808 as a DDS, and flow cytometry using calcein stained samples has shown that the glycopolymer coating enhances their cellular uptake, highlighting the efficacy of both MOF-808 as a DDS and the dual drug delivery protocol for anticancer cytotoxicity.

## Results and Discussion

Among a large number of MOF structures, Zr-based MOFs^57^ have attracted much attention due to their structural and chemical stabilities that make them suitable for a range of applications, not least drug delivery.^29^ MOF-808(Zr), a Zr-MOF first reported by the Yaghi group in 2014,^56^ offers many advantages including high surface area, large cavities, and abundant metal sites.^58^ MOF-808 is composed of [Zr_6_O_4_(OH)_4_(CO_2_)_6_(HCO_2_)_6_] secondary building units (SBUs) connected by 1,3,5-benzenetricarboxylate (BTC) ligands, resulting in a highly porous structure exhibiting the **spn** topology. The six remaining equatorial sites are coordinated with an additional monotopic ligands, such as formate, acetate, or propionate, as charge compensators and as crystal growth modulators to improve the crystallinity of the framework and control crystal size.^59^ The strong coordination bond between hard Zr^4+^ ions and hard carboxylate O-donors in the structure provides MOF-808 with excellent stability in thermal and acidic environments. The terminal anions of monotopic ligands on the remaining sites of the eight-coordinated Zr(IV) centers exhibit relative lability, allowing for postsynthetic modification (PSM) with therapeutics and targeted functional groups.^60−62^ Despite these advantages, MOF-808 has rarely been used as a drug delivery system compared to the much more widely studied Zr MOF UiO-66, potentially due to the fact that MOF-808 has been identified as an excellent catalyst for peptide hydrolysis.^60,63^ Nevertheless, Zhang and co-workers have shown an efficient pH-dependent drug delivery of 5-FU toward HeLa cervical adenocarcinoma cells using folate-targeted MOF-808 with no apparent in vitro cytotoxicity of the MOF itself.^62^ Davoodi and co-workers have used MOF-808 as an iodophore antimicrobial agent, reporting a 19 mm zone of inhibition against *Staphylococcus aureus* with controlled and sustained release of iodine from iodine-loaded MOF-808.^64^ In this study, we have shown MOF-808 can act as a cancer-targeted dual drug delivery system by combining FUDR and CARB in MOF structure for a synergistic combination therapy and by decorating MOF-808 with a glycopolymer for targeting cancer cells.

## Synthesis and Characterization

**Scheme 1:**
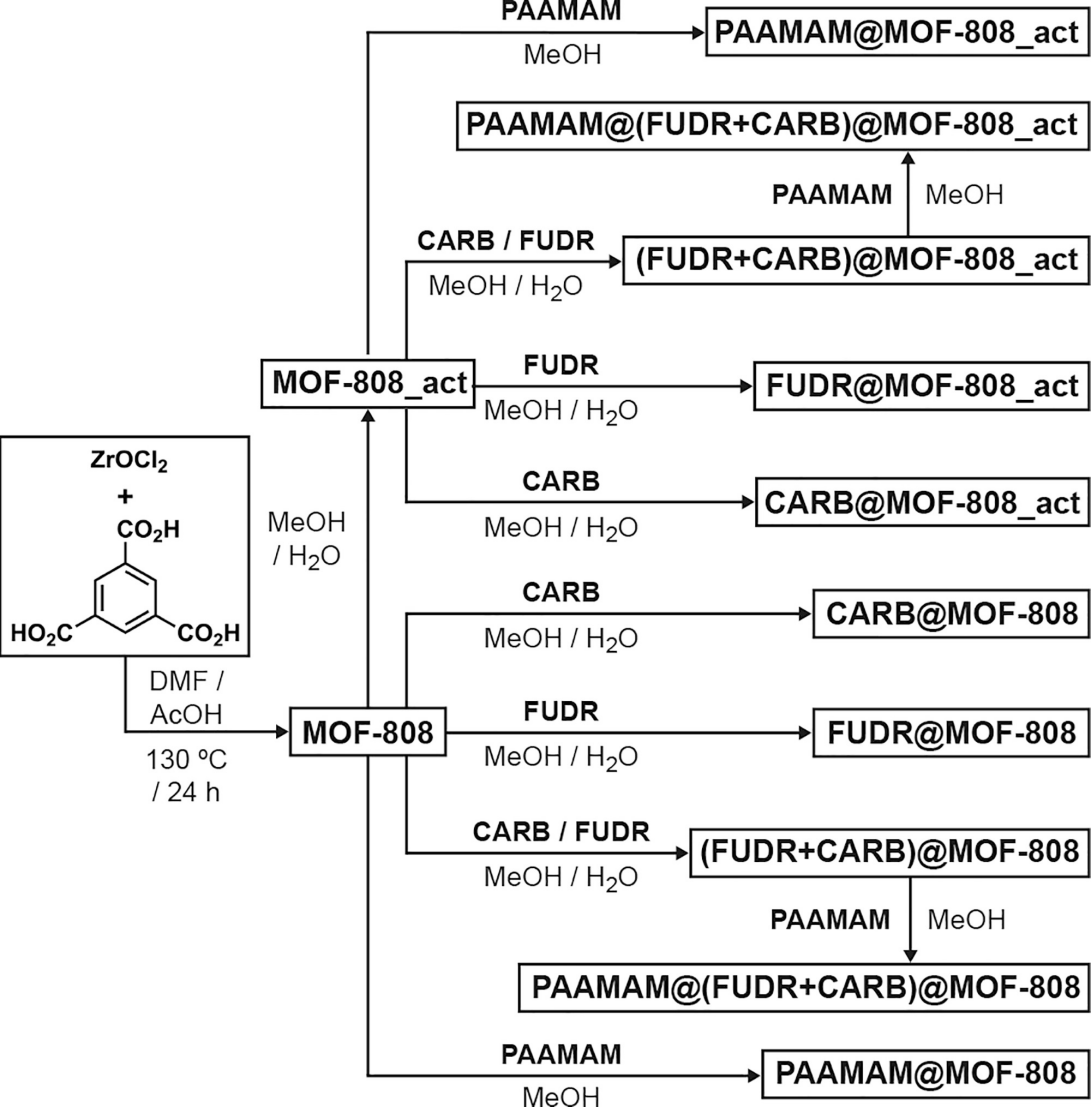
Sequential Synthesis of MOF-808, Its Activation with Methanol (MeOH) and Water (H_2_O), Drug Loading, and Polymer Coating

MOF-808 was synthesized by a solvothermal protocol using Zr precursors and benzene-1,3,5-tricarboxylic acid as well as acetic acid as a modulator (Scheme 1). Residual solvents were removed by sequential DMF and acetone washes, followed by vacuum drying. During optimization of drug-loading protocols, whereby FUDR and/or CARB were loaded into the MOF-808 nanoparticles in a mixed methanol (MeOH) and water (H_2_O) solution which was capable of dissolving both drugs, it was observed that the loading protocol served to further enhance the porosity of some of the samples, effectively activating them further. As such, a comparison was made between the as-synthesized MOF-808 nanoparticles and those that had deliberately been further activated with MeOH/H_2_O, termed MOF-808_act.

Powder X-ray diffraction (PXRD, Figure 2a) showed the successful formation of crystalline MOF-808 and the retention of crystallinity upon activation to MOF-808_act. Scanning electron microscopy (SEM, Figure 2b) showed MOF-808 is formed as octahedral nanoparticles of approximately 100 nm (see Figures S1–S12 for size analysis) with morphology retained upon activation.

**Figure 2:**
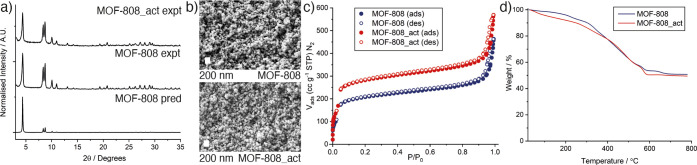
Characterization of MOF-808 and MOF-808_act. (a) Stacked experimental powder X-ray diffractograms compared to that predicted from the crystal structure.^56^ (b) Scanning electron micrographs of the nanoparticulate samples (scale bars 200 nm). (c) N_2_ adsorption/desorption isotherms (77 K). Corresponding pore size distributions are shown in Figure S13. (d) Thermogravimetric analyses of the samples carried out in air. Derivative mass change is shown in Figure S14.

The N_2_ adsorption desorption isotherms show both samples are porous, with significant interparticle uptake at *P*/*P*_0_ ∼ 0.95 characteristic of their nanoparticulate morphologies (Figure 2c). The MeOH/H_2_O activation protocol significantly increases the porosity; MOF-808 has *S*_BET_ = 792 m^2^ g^–1^ and a pore volume of 0.471 cc g^–1^, which increase to 1070 m^2^ g^–1^ and 0.607 cc g^–1^, respectively, for MOF-808_act. It is not clear why this activation process increases porosity to this extent, as thermogravimetric analysis (TGA) shows the samples have little defectivity or residual solvent (Figure 2d). Assuming an ideal formula of [Zr_6_O_4_(OH)_4_(C_9_H_3_O_6_)_2_(CH_3_COO)_6_], a ZrO_2_ residue of 51.0% would be expected, with experimental values for MOF-808 (50.5%) and MOF-808_act (49.5%) correlating closely.

The PAAMAM glycopolymer was synthesized via RAFT polymerization of d-mannose acrylamide and acrylic acid in a mixture of DMF/H_2_O (70/30 *v*/*v*) with [[(butylthio)carbonothioyl]thio]propanoic acid (PABTC) acting as the RAFT agent and 4,4′-azobis(4-cyanovaleric acid) (ACVA) as initiator (Scheme S1). ^1^H NMR spectroscopy showed full conversion to the glycopolymer by the disappearance of the resonances assigned to the vinyl groups of the monomers at δ = 5.5–6.5 ppm and the appearance of resonances assigned to the polymer backbone at δ = 1.2–2.3 ppm (Figure S15). Gel-permeation chromatography measurements of the polymer were carried out in an aqueous system which showed a narrow disperse peak with *M*_n,GPC_ = 8400 g mol^–1^ and *Đ* = 1.1 (Figure S16).

**Figure 3:**
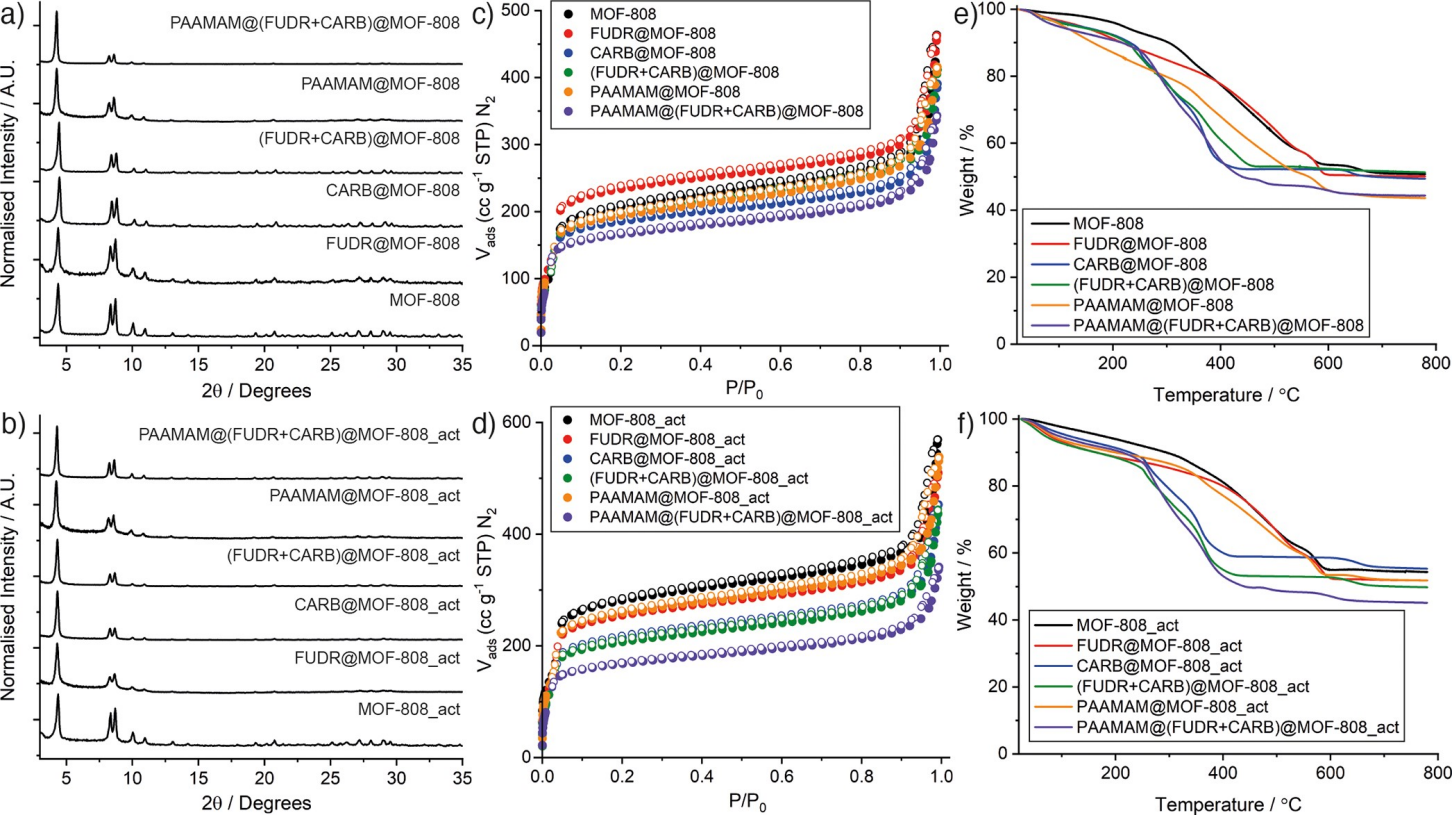
Physical characterization of empty and loaded MOF-808 and MOF-808_act nanoparticles. Stacked powder X-ray diffractograms of empty and loaded samples of (a) MOF-808 and (b) MOF-808_act. N_2_ adsorption/desorption isotherms (77 K) of empty and loaded samples of (c) MOF-808 and (d) MOF-808_act. Filled symbols represent adsorption, and empty symbols represent desorption. Corresponding pore-size distributions can be found in Figures S18 and S19. TGA traces of empty and loaded samples of (e) MOF-808 and (f) MOF-808_act. Corresponding analyses of first derivatives can be found in Figures S20 and S21.

Both MOF-808 and MOF-808_act were individually and dually loaded with FUDR and CARB in MeOH/H_2_O to attempt to achieve a synergistic effect and decrease the overall administrative dose. Subsequently, the dual drug-loaded nanoparticles and control samples of the empty MOFs were postsynthetically coated with PAAMAM in MeOH as shown in Scheme 1. The multiple polar functional groups on the drugs are expected to form noncovalent interactions with the polar Zr_6_ SBUs in MOF-808, while the PAAMAM polymer has rich carboxylate functionality to coordinate to the Zr_6_ units at the particle surfaces, which is a well-established strategy for surface modification of MOFs.^44^ Physical characterization is presented in Figure 3, delineated for samples of MOF-808 and MOF-808_act. Stacked powder X-ray diffraction (PXRD) patterns of MOF-808 indicated highly crystalline nanoparticles with retained crystallinity after postsynthetic drug loading and PAAMAM coating (Figures 3a and 3b). The amorphous structure of PAAMAM (Figure S17) did not affect the overall crystallinity of MOFs.

The N_2_ adsorption isotherms (Figures 3c and 3d) showed that all the nanoparticles are porous, with the general trend that loading of drug molecules into the MOFs results in a decrease in gravimetric uptake and BET surface area, as a consequence of incorporation of additional nonporous mass as well as loaded cargo taking up pore space within the MOF (Table 1). This is further evidenced by a decrease in intensity of the larger pore and a decrease in size of the narrow pores in the pore size distributions (Figures S18 and S19). The only exception is that FUDR loading of MOF-808 increased the porosity, which we believe is a combination of the MeOH/H_2_O loading protocol further activating the MOF and the relatively low FUDR loading. The porosity of FUDR@MOF-808 (*S*_BET_ = 888 m^2^ g^–1^, pore volume = 0.495 cc g^–1^) does remain lower than that of MOF-808_act, suggesting some loading of FUDR. Polymer coating also results in a decrease in porosity, which is indicative of incorporation of additional mass and/or blocking pore apertures at the particle surfaces. When both (FUDR+CARB)@MOF-808 and (FUDR+CARB)@MOF-808_act were coated with PAAMAM in MeOH, their surface areas decreased further, which suggests successful polymer coating without significant cargo leakage.

**Table 1:** Characterization Data for MOF-808 and MOF-808_act and Their Functionalized Derivatives

sample	BET surface area (m^2^/g)^a^	pore volume (cc/g)^a^	FUDR (%, w/w)^b^	CARB (%, w/w)^c^	estimated PAAMAM coating (%, w/w)^d^	size (nm)^e^	hydrodynamic size (nm)^f^	PDI^f^	zeta potential (mV)^f^
MOF-808	792	0.471	-	<0.02	-	108.4 ± 27.8	185.8 ± 28.6	0.330 ± 0.024	–21.3 ± 0.4
FUDR@MOF-808	888	0.495	1.18 ± 0.02	-	-	104.6 ± 25.5	150.7 ± 10.8	0.385 ± 0.016	–20.6 ± 0.3
CARB@MOF-808	697	0.409	-	10.99	-	110.2 ± 24.9	210.4 ± 7.6	0.406 ± 0.016	–16.5 ± 0.2
(FUDR+CARB)@MOF-808	736	0.444	0.59 ± 0.02	9.20	-	116.5 ± 29.1	200.5 ± 32.9	0.450 ± 0.043	–9.9 ± 0.3
PAAMAM@MOF-808	722	0.456	-	-	4.00	113.7 ± 25.9	255.9 ± 20.7	0.239 ± 0.014	–29.7 ± 0.1
PAAMAM@(FUDR+CARB)@MOF-808	631	0.371	0.46 ± 0.01	8.26	4.07	101.8 ± 23.2	253.0 ± 21.0	0.496 ± 0.077	–34.1 ± 0.9
MOF-808_act	1070	0.607	-	<0.02	-	110.9 ± 24.6	169.0 ± 6.6	0.410 ± 0.082	–18.7 ± 0.7
FUDR@MOF-808_act	958	0.557	1.29 ± 0.04	-	-	94.4 ± 18.7	199.5 ± 12.7	0.505 ± 0.055	–16.3 ± 0.6
CARB@MOF-808_act	804	0.482	-	13.74	-	106.6 ± 26.0	219.2 ± 13.8	0.515 ± 0.039	–12.6 ± 0.5
(FUDR+CARB)@MOF-808_act	770	0.47	0.63 ± 0.06	12.14	-	109.1 ± 24.0	216.1 ± 10.4	0.458 ± 0.031	–13.8 ± 0.3
PAAMAM@MOF-808_act	977	0.569	-	-	7.12	102.5 ± 24.2	197.5 ± 9.3	0.498 ± 0.033	–35.8 ± 1.13
PAAMAM@(FUDR+CARB)@MOF-808_act	637	0.377	0.69 ± 0.01	9.59	6.33	112.9 ± 30.3	230.0 ± 6.2	0.342 ± 0.009	–35.2 ± 1.1

a Derived from N_2_ adsorption isotherms (77 K).

b Measured by HPLC.

c Calculated from the ICP-OES measurement of Pt content in the samples.

d Calculated from TGA analysis.

e Average size distribution measured by randomly selecting 100 particles from an SEM image for each MOF sample.

f Measured by DLS Zetasizer.

Thermogravimetric analysis (TGA) was used to qualitatively assess drug loading in MOF-808 and MOF-808_act (Figure 3e and 3f). In general, drug loading induced thermal degradation at lower temperatures but did not cause any significant change in the residual mass, most probably due to low FUDR loading and the fact that CARB contains 52% (*w*/*w*) Pt, which would contribute to the residue. These facts, plus the complex and overlapping mass loss events, made quantification of drug loading impossible by this technique. This is evident in the derivative weight changes of the nanoparticles for MOF-808 (Figures S14 and S20) and MOF-808_act (Figures S14 and S21), which however do confirm qualitatively the incorporation of the drugs and the PAAMAM due to the similarity in their decomposition profiles (Figures S22 and S23). In contrast, the PAAMAM functionalization of both empty and loaded MOFs clearly decreased the mass of the inorganic residue, indicating increased organic content of the samples and so successful polymer incorporation. Comparing the traces for samples before and after coating showed that MOF-808 was coated with ∼4% (*w*/*w*) polymer, while MOF-808_act was coated with 6–7% (*w*/*w*) polymer. As PAAMAM is expected to surface modify the MOFs through the high affinity of the Zr^4+^ cations to the carboxylate functionalities of the PAAMAM,^65,66^ the higher PAAMAM coating with MOF-808_act might be a result of the activation process making more sites available at the particle surfaces.

Both FTIR spectroscopic analysis of the solids (Figures S24–S28) and ^1^H NMR spectroscopic analysis of acid digested samples (Figures S29–S34) qualitatively confirmed the presence of CARB and PAAMAM in the corresponding samples, although it was not possible to observe resonances for FUDR in the NMR spectra, nor peaks in the IR spectra, which are again indicative of low loading. NMR spectroscopic analysis is also hindered by the acid digestion protocol resulting in the disappearance and/or shifting of some characteristic peaks for FUDR and CARB (Figures S30 and S32).

To quantify drug content, two techniques were employed. CARB loading of the samples was measured by inductively coupled plasma optical emission spectroscopy (ICP-OES) to determine the Pt content in the MOF samples. FUDR loading of MOFs was determined by high-performance liquid chromatography (HPLC) analysis using degradation solutions of the nanoparticles and freshly prepared FUDR standard solutions (Figure S35). The results seen in Table 1 show that while CARB loading of the nanoparticles is quite high, between 8.26% and 13.74% (*w*/*w*), FUDR loading is one order of magnitude lower, between 0.46% ± 0.01 and 1.29% ± 0.04 (*w*/*w*). Dual drug loading was also successful, albeit with slightly lower loading values for each drug compared to the individually loaded samples. PAAMAM coating resulted in only small decreases in drug content, which would be expected from the overall increase in the total mass of the DDS causing a decrease in the ratio of drug to MOF and suggesting minimal leakage during surface modification. Across the samples, the CARB:FUDR mass ratio was maintained at approximately 14 to 19 equiv of CARB to FUDR. In general, the MeOH/H_2_O activation protocol was associated with enhanced drug loading, correlating well with the increase in porosity of MOF-808_act compared to MOF-808.

Scanning electron microscopy (SEM) was used for morphological analysis, confirming that the characteristic octahedral morphology of the MOFs was maintained during all postsynthetic modifications, including all drug loading and polymer coating (see Figures S1–S12 for size analysis). The samples were well-dispersed, with an average particle size of 108.4 ± 27.8 nm, and no significant changes in average particle size and morphology across the MOF samples (Table 1). The colloidal stability of the nanoparticles was investigated in water (pH = 7.4) by dynamic light scattering (DLS). As seen in Table 1, all nanoparticles show a good size range for applications in biomedicine of approximately 150–250 nm. The hydrodynamic size of MOF-808 is 185.8 ± 28.6 nm with a polydispersity index (PDI) of 0.330 ± 0.024 which suggests a small aggregation compared to the SEM data, while MeOH/H_2_O activation of MOF-808 resulted in an insignificant drop in the hydrodynamic size. In general, FUDR and/or CARB postsynthetic drug modifications caused a small increase in hydrodynamic size and a concomitant small reduction in the negative zeta potential, with the magnitudes of change larger for samples containing a greater weight percentage of drug. Due to the relatively small PAAMAM content (∼5% w/w) we would not expect to observe significant size differences in the SEM analysis due to the expected low thickness of the polymer coating. However, PAAMAM coating consistently enlarged the hydrodynamic particle size measured by DLS, while significantly increasing the anionic zeta potential of the nanoparticles, confirming successful integration of this carboxyl-rich PAAMAM as a surface coating on the nanoparticles.

## Cytotoxicity and Cellular Uptake

The efficacy of drug loaded MOF-808 and MOF-808_act and effect of the PAAMAM surface coating on cellular uptake and cytotoxicity have been evaluated in the human cancer cell lines MCF-7 (breast adenocarcinoma), PANC-1 (pancreatic ductal adenocarcinoma), and HepG2 (hepatocellular carcinoma). Cell viability was determined using the Alamar Blue cell viability assay at 24 and 72 h time points, and the half maximal inhibitory concentration (IC_50_) values were determined where possible. Control experiments using free CARB, FUDR, and PAAMAM were also carried out in-house for direct comparison.

In general, the nanoparticles did not induce significant cytotoxicity after 24 h incubation, with most cell viability values remaining greater than 80% (Figures S36 and S37). This was also observed for the control experiments where the cell lines were incubated with the free drugs for 24 h. Both MOF-808 and MOF-808_act do not show significant cytotoxicity after 24 h incubation, and neither does free PAAMAM. However, it was noted that, after 24 h, the samples that had reduced cell viability to the greatest extent were PAAMAM coated, potentially suggesting a more significant uptake of the polymer-coated MOFs. For all cell lines, the most cytotoxic materials after 24 h were PAAMAM@(FUDR+CARB)@MOF-808 and PAAMAM@(FUDR+CARB)@MOF-808_act, with cell viabilities in the 65–85% region.

**Figure 4:**
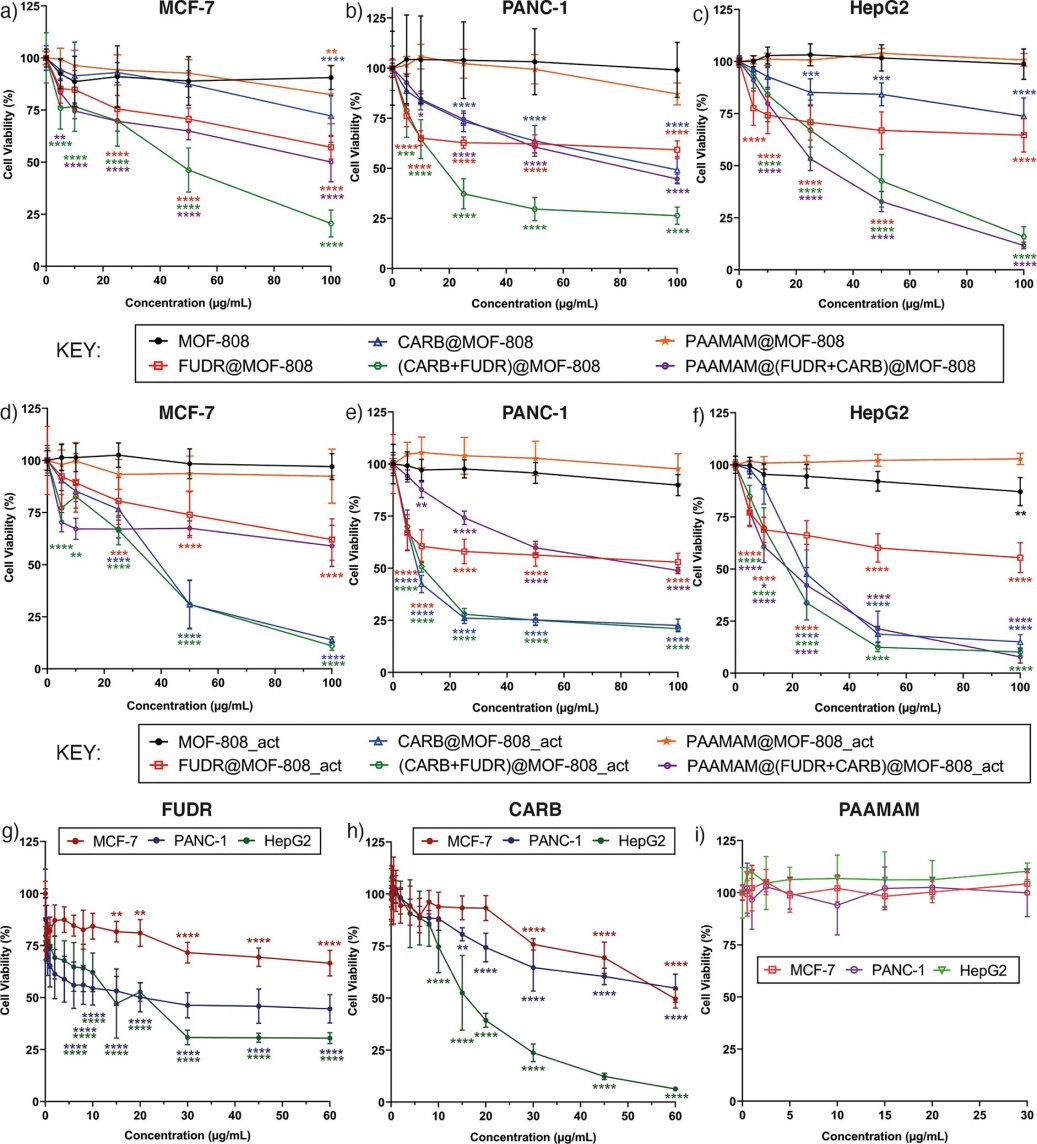
Cell viabilities of (a) MCF-7, (b) PANC-1, and (c) HepG2 cells treated with MOF-808 and its drug loaded and PAAMAM coated formulations, compared to cell viabilities of (d) MCF-7, (e) PANC-1, and (f) HepG2 cells treated with MOF-808_act and its drug loaded and PAAMAM coated formulations. Cell viabilities of the three cell lines when treated with (g) free FUDR, (h) free CARB, and (i) PAAMAM are also provided for comparison. All incubations carried out for 72 h. Viabilities assessed by the Alamar blue cell viability assay with untreated cells accepted as control. The data were expressed as mean and standard deviation (*n* = 3 × 3), *p* ≤ 0.05 (*), *p* ≤ 0.01 (**), *p* ≤ 0.001 (***), and *p* ≤ 0.0001 (****). The stars show the statistical significance of each data point from the control. An inset for part g to show statistical significance below 10 μg mL^–1^ is provided in Figure S38. The equivalent FUDR and CARB concentrations for each nanoparticle concentration used (between 5 and 100 μg mL^–1^) are reported in Table S1, and the data plotted as a function of CARB concentration are given in Figure 5, where appropriate.

Cytotoxicities were much more pronounced after 72 h incubation and are presented in Figure 4 as a function of the DDS concentration for ease of initial comparison. For all cell lines, the empty MOFs, the PAAMAM-coated empty MOFs, and PAAMAM itself did not induce any significant cytotoxicity, with cell viabilities well above 80% in all cases. In contrast, the drug-loaded MOFs reduced cell viability considerably. Despite the low drug loading values (∼1% (*w*/*w*)), both FUDR@MOF-808 and FUDR@MOF-808_act decreased the cell viability to around 50–75% across the different cell lines, with no significant difference between the two samples probably due to the similar FUDR loading of MOF-808 and MOF-808_act. Much more significant cytotoxicity was observed for the CARB-loaded MOFs, where CARB@MOF-808_act was seen to show increase cytotoxicity at 72 h compared to CARB@MOF-808. As seen in Table 1, activation of MOF-808 improves the CARB loading from 10.99% to 13.74%, which seems to have made it a more potent DDS against all three cancer cell lines. These enhancements in cytotoxicity confirm that the DDSs are stable enough to avoid rapid burst release or degradation in cell media prior to their endocytosis.

To compare samples with different percentage drug loading amounts, IC_50_ values were calculated where possible using GraphPad Prism 9 software package (GraphPad Software, Inc., San Diego, CA) and are presented in Table 2, normalized to the calculated drug content presented in Table 1. CARB@MOF-808 nanoparticles have an IC_50_ value of 96.7 μg mL^–1^ in PANC-1 cells for 72 h incubation, which corresponds to 10.6 μg mL^–1^ of CARB. The MeOH/H_2_O activated sample, CARB@MOF-808_act, further increased the cytotoxicity of CARB, decreasing the nanoparticle IC_50_ to 8.4 μg mL^–1^, with an equivalent CARB IC_50_ of 1.2 μg mL^–1^, indicating that the enhanced cytotoxicity compared to CARB@MOF-808 is not solely due to the higher CARB loading. These values were both significantly better than that of free CARB, which showed no IC_50_ value in PANC-1 in the used concentration range (up to 60 μg mL^–1^) and confirmed that there is a much greater efficacy of CARB when incorporated into MOF nanoparticles. Similar results are achieved for the MCF-7 and HepG2 cell lines, where CARB@MOF-808_act again outperforms CARB@MOF-808, even when CARB loading values are normalized, and shows a 10-fold and 5-fold decrease in IC_50_ against MCF-7 and HepG2 cells, respectively. These results suggest that even unfunctionalized MOF-808 is an excellent vector for CARB delivery, and that activation protocols significantly influence in vitro cytotoxicity, which may be a consequence of differing CARB loading locations within the more porous MOF-808_act.

**Table 2:** IC_50_ Values for the Cell Lines Incubated with the Samples for 72 h^a^

	IC_50_(μg mL^–1^)								
	MCF-7			PANC-1			HepG2		
sample	NP^b^(μg mL^–1^)	equiv^c^ FUDR (μg mL^–1^)	equiv CARB (μg mL^–1^)	NP (μg mL^–1^)	equiv FUDR (μg mL^–1^)	equiv CARB (μg mL^–1^)	NP (μg mL^–1^)	equiv FUDR (μg mL^–1^)	equiv CARB (μg mL^–1^)
MOF-808	-	-	-	-	-	-	-	-	-
FUDR@MOF-808	-	-	-	-	-	-	-	-	-
CARB@MOF-808	-	-	-	96.7	-	10.6	-	-	-
(FUDR+CARB)@MOF-808	45.9	0.27	4.2	17.8	0.11	1.6	42.3	0.25	3.9
PAAMAM@MOF-808	-	-	-	-	-	-	-	-	-
PAAMAM@(FUDR+CARB)@MOF-808	100	0.46	8.3	82.6	0.38	6.8	28.7	0.13	2.4
MOF-808_act	-	-	-	-	-	-	-	-	-
FUDR@MOF-808_act	-	-	-	-	-	-	-	-	-
CARB@MOF-808_act	39.4	-	5.4	8.4	-	1.2	24.0	-	3.3
(FUDR+CARB)@MOF-808_act	36.4	0.23	4.4	10.2	0.06	1.2	18.0	0.11	2.2
PAAMAM@MOF-808_act	-	-	-	-	-	-	-	-	-
PAAMAM@(FUDR+CARB)@MOF-808_act	-	-	-	94	0.65	9.0	18.5	0.13	1.8
FUDR	-	-	-	-	20.0	-	-	14.0	-
CARB	-	-	59.4	-	-	-		-	15.8
PAAMAM	-	-	-	-	-	-	-	-	-

a Unreported values are the values that could not be determined in the used sample concentration range.

b NP: nanoparticle;

c equiv: equivalent.

**Figure 5:**
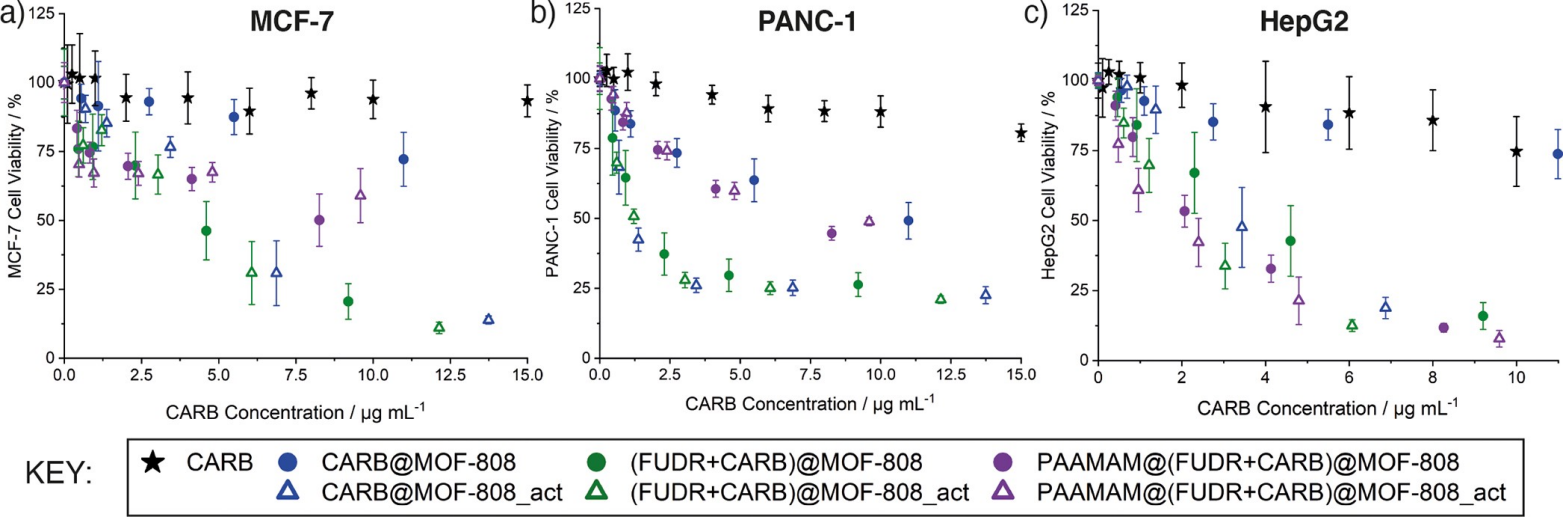
Cell viabilities, expressed as a function of CARB concentration, of (a) MCF-7, (b) PANC-1, and (c) HepG2 cells treated with CARB-loaded MOFs for 72 h, compared to treatment with free CARB, analyzed by Alamar blue cell viability assay. Untreated cells were accepted as control. It was not possible to prepare similar plots normalized to FUDR concentration due to the low FUDR loading values.

Dual drug loading of MOFs enhanced the therapeutic effect of the drugs and dramatically decreased the cell viability in all cell lines for 72 h incubation. Delineating the effects of the two drugs is difficult due to the different ratios and loading contents of FUDR versus CARB in each dual drug delivery sample. The IC_50_ values of the dual drug treatments have been normalized to both the FUDR and the CARB content in Table 2 to allow some comparison, and it is clear that there is a notable benefit of using two drugs together, as the normalized IC_50_ values for each drug in the dual delivery systems are lower than those of the free drugs and the single drug delivery systems. When values are normalized to FUDR content, the IC_50_ values for the dual drug delivery systems are enhanced by up to 2 orders of magnitude. For example, free FUDR has an IC_50_ of 20.0 μg mL^–1^ in PANC-1 and 14.0 μg mL^–1^ in HepG2; after 72 h incubation with (FUDR+CARB)@MOF-808, this dropped to 0.11 μg mL^–1^ and 0.25 μg mL^–1^, respectively, when normalized to FUDR concentration. In MCF-7 cells, where an IC_50_ could not be recorded even up to 60 μg mL^–1^ free FUDR, the (FUDR+CARB)@MOF-808 had an IC_50_ normalized to FUDR content of 0.27 μg mL^–1^. This apparently dramatic increase in cytotoxicity is a consequence of the much larger CARB loading compared to FUDR loading, so a more valid comparison would be to compare cytoxicity and IC_50_ values normalized to CARB content. For the CARB containing samples, the in vitro cell viability data have been presented as a function of CARB content in Figure 5 to aid visualization of the different effects on overall cytotoxicity.

When normalized to CARB content, the IC_50_ values for the dual drug systems decreased compared to the free drug and single drug-loaded system, with the differences much more significant when comparing the nonactivated samples, CARB@MOF-808 and (FUDR+CARB)@MOF-808, than when comparing the MeOH/H_2_O preactivated samples, CARB@MOF-808_act and (FUDR+CARB)@MOF-808_act. As a consequence, the IC_50_ values for (FUDR+CARB)@MOF-808 and (FUDR+CARB)@MOF-808_act are very similar when normalized to CARB content, which might be a result of the higher CARB loading of the activated samples and possible drug resistance of cancer cells after a certain drug concentration.^67^ For example, against MCF-7 cells, the IC_50_ values for (FUDR+CARB)@MOF-808 and (FUDR+CARB)@MOF-808_act normalized to CARB loading are 4.2 and 4.4 μg mL^–1^, respectively, slightly lower than CARB@MOF-808_act (5.4 μg mL^–1^ CARB) but significantly lower compared to free CARB (59.4 μg mL^–1^). Nevertheless, the decreases in CARB-normalized IC_50_ values for the dual drug-loaded samples compared to the single drug-loaded samples confirm the beneficial effect of concomitantly delivering FUDR with CARB and show that this system is a rare example of combination drug delivery from MOF nanoparticles.^68−74^

The mannose binding lectin receptors (mannose receptors, CD206) are overexpressed on many cells, including immune system cells (macrophages, dendritic cells) and various cancer cells such as liver cancer.^75^ As such, it was hoped that the PAAMAM coating would further enhance the cytotoxicity of the samples due to enhanced uptake, but results were variable when compared to the uncoated analogues. Here PAAMAM coating of the nanoparticles improved their cytotoxicity against HepG2 cells; normalized to CARB loading, the PAAMAM coating further reduces IC_50_ values. Higher expression of CD206 in liver cancer samples compared to healthy adjacent liver tissue has been reported by Fan et al. previously.^75^ Kim et al. have also shown enhanced cellular internalization of mannose-conjugated ionizable lipid nanoparticles (LNPs) compared to unconjugated LNPs in HepG2 cells via CD206.^76^ For the nonactivated samples, going from (FUDR+CARB)@MOF-808 to PAAMAM@(FUDR+CARB)@MOF-808 reduces IC_50_ from 3.9 μg mL^–1^ to 2.4 μg mL^–1^, while for the activated samples, going from (FUDR+CARB)@MOF-808_act to PAAMAM@(FUDR+CARB)@MOF-808_act slightly reduces IC_50_ from 2.2 μg mL^–1^ to 1.8 μg mL^–1^. This was not found to be statistically significant, probably due to the drug resistance of cancer cells as seen with the CARB@MOF-808_act samples (IC_50_ 3.3 μg mL^–1^ CARB). In contrast, the PAAMAM-coated nanoparticles showed slightly less cytotoxicity over 72 h incubation compared to their uncoated analogues toward both MCF-7 and PANC-1 cells. Nevertheless, significant enhancements in cytotoxicity toward MCF-7 and PANC-1 compared to free CARB are maintained, and this different response may indicate potential for development of selectivity in anticancer activity in vivo based on differing cellular receptor profiles.

The main conclusion of the in vitro assays is that MOF-808 can enhance the cytotoxicity of FUDR through nanoparticulate delivery and is a highly promising vector for CARB delivery, where orders of magnitude increases in anticancer cytotoxicity can be observed even for uncoated, single-drug-loaded samples. In single drug delivery, the activation protocol for MOF-808 is key to enhanced cytotoxicity, but in dual delivery of CARB and FUDR, while cytotoxicity is further enhanced, the reliance on different activation protocols is less pronounced. PAAMAM coating has variable effects on cytotoxicity, but further enhancement is observed against HepG2 cells. This may be a consequence of different cellular receptor profiles or modification of endocytosis routes through MOF surface modification, but the maintained cytotoxicity enhancements compared to the free drugs are highly promising.

On the basis of the promising evidence from the cytotoxicity studies, cellular uptake was then investigated using flow cytometry at 24 h incubation, to avoid the significant cell death observed for longer incubation times (Figure 4) which might result in the damage of cellular membrane and lysis of cytoplasm and intracellular MOFs. The nanoparticles were stained with a fluorophore, calcein (Figure S39 and Table S2), which we have previously used to determine MOF uptake within cells, as it is not significantly endocytosed as a free molecule due to its hydrophilicity.^45,77−79^ As such, enhanced intracellular calcein fluorescence when delivered by a MOF compared to free calcein can be used as a proxy for MOF endocytosis.

**Figure 6:**
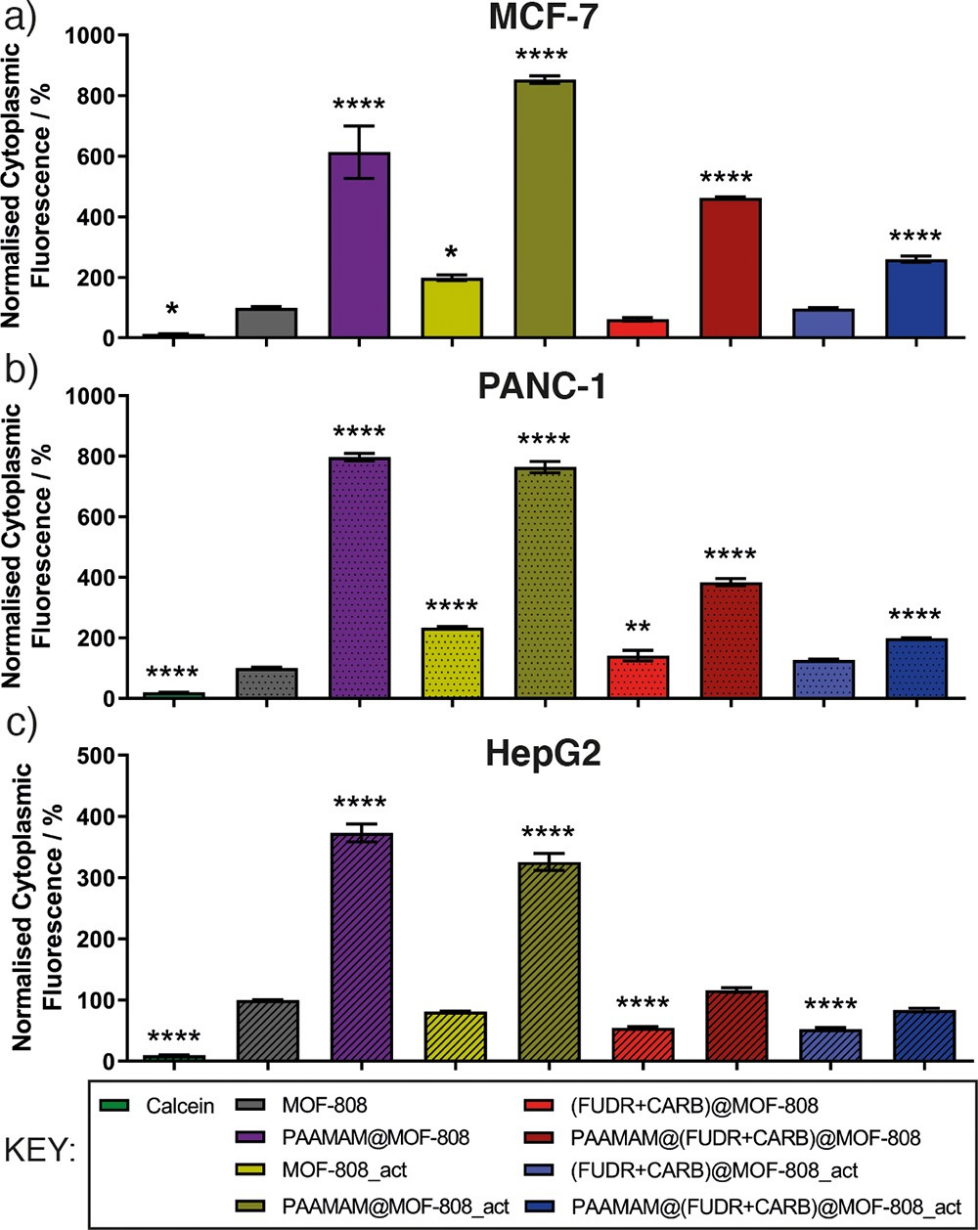
Normalized intracellular fluorescence of (a) MCF-7, (b) PANC-1, and (c) HepG2 cells incubated with MOF samples. MOFs were loaded with fluorophore calcein for fluorescence-based intracellular detection via flow cytometry. Fluorescence values are normalized to MOF-808 (100%).

In all cell lines, flow cytometry showed that MOF-808 can transport significant amounts of calcein into the cells, while the PAAMAM coating significantly improved the cellular uptake of the nanoparticles further in all cell lines compared to uncoated analogues (Figure 6). For example, in MCF-7 cells, PAAMAM@MOF-808 showed a 6-fold increase in uptake of calcein compared to MOF-808, and an 8.5-fold uptake enhancement in PANC-1. These enhancements in endocytosis are significantly greater than those we have achieved previously using folic acid-modified Zr MOFs to target folate receptor-positive cell lines such as HeLa. In these cases, significant enhancements in cytotoxicity of dichloroacetate were observed, which were ascribed to modification of specific endocytosis pathways rather than total uptake.^80^ A similar enhancement in cytotoxicity of 5-FU delivered from folic acid-modified MOF-808 has also been reported, but quantitative endocytosis enhancements were not determined in this study.^62^ The MeOH/H_2_O activation protocol also enhanced cellular internalization of MOF-808 in MCF-7 cells, for both coated and uncoated samples, but the effect was not so pronounced in PANC-1 or HepG2 cells. Nevertheless, the PAAMAM coating significantly enhanced the endocytosis of the empty MOFs by up to 850%. The protocol was also applied to drug-loaded samples of MOF-808 following the postsynthetic drug modifications. FUDR and/or CARB loading did not cause any significant change in the cellular uptake, but surface functionalization of these drug-loaded MOFs by PAAMAM again enhanced their cellular internalization, although to a lesser extent than the empty samples, which may explain why significant enhancements in cytotoxicity were not observed for these drug-loaded MOFs in general.

## Conclusion

In summary, we have shown that nanoscale MOF-808 is an excellent candidate for drug delivery applications, by demonstrating the following: (i) orders of magnitude increases in the in vitro cytotoxicity of carboplatin when delivered from MOF-808, (ii) the ability to deliver two drugs, carboplatin and floxuridine, from one nanoparticle with synergistic cytotoxic effects, and (iii) its amenability to surface functionalization with a glycopolymer and subsequent enhancements in endocytosis efficiency. By significantly enhancing the anticancer cytotoxicity of CARB and FUDR, it may be possible to reduce dosages and minimize undesirable off-target effects through delivery with MOF-808. The dramatically enhanced endocytosis of MOF-808 nanoparticles when coated with PAAMAM also suggests potential for in vivo targeting, which will be a future avenue of study. We have also demonstrated the importance of full activation of MOF-808 prior to drug loading, which not only enhances overall loading of carboplatin but delivers further improvements in cytotoxicity even when the carboplatin concentration being delivered is normalized. This indicates that the location of the cargo within the MOF-808 nanoparticle is of importance in delivery efficacy^81^ and is something that can be further optimized in concert with the installation of surface polymer units. It also calls into question the ability to appropriately compare results between different laboratories where different activation protocols may be used. Further investigation of MOF-808 as a drug delivery vector, including in vitro and in vivo stability, biocompatibility, and hybridization with alternative carbohydrate functionality,^82^ is underway.

## Supporting Information Available

Additional experimental details, materials and methods, synthetic procedures, further characterization data, and experimental results (PDF)
