# Supplementary material for: Glycopolymer-Functionalized MOF-808 Nanoparticles as a Cancer-Targeted Dual Drug Delivery System for Carboplatin and Floxuridine
Source: ACS Appl Nano Mater. 2022 Jun 22;5(10):13862–73. doi: 10.1021/acsanm.2c01632 (PMC9623548; doi:10.1021/acsanm.2c01632)
Supplement: Supplementary file 1 — an2c01632_si_001.pdf [file an2c01632_si_001.pdf]

SUPPORTING INFORMATION

Glycopolymer-Functionalised MOF-808 Nanoparticles as a Cancer-Targeted Dual Drug Delivery System for Carboplatin and Floxuridine

Fatma Demir Duman[a], Alessandra Monaco[b], Rachel Foulkes[a], C. Remzi Becer[b], Ross S. Forgan[a*]

[a]WestCHEM, School of Chemistry, University of Glasgow, University Avenue, Glasgow G12 8QQ, UK.

[b]Department of Chemistry, University of Warwick, CV4 7AL Coventry, UK.

E-mail: ross.forgan@glasgow.ac.uk

S1. Materials and Methods

S1.1. Materials

Zirconyl chloride octahydrate ($ZrOCl_2 \cdot 8H_2O$, 98+%) was purchased from Acros Organics. Acetic acid (glacial) was purchased from VWR. *N*,*N*-dimethylformamide (DMF) was obtained from Honeywell. Methanol and acetonitrile were purchased from Fisher Scientific. Floxuridine (FUDR) and carboplatin (CARB) were purchased from Tokyo Chemical Industry Co., Ltd. Roswell Park Memorial Institute 1640 medium (RPMI 1640, 1x, catalogue number: 21870076), Minimum Essential Medium (MEM) medium (catalogue number: 11090081), fetal bovine serum (FBS), 100 mM sodium pyruvate and 0.25% trypsin-EDTA (1x) were obtained from Thermo Fisher Scientific, Gibco. MEM non-essential amino acid solution (100x) (without L-glutamine), penicillin-streptomycin (with 10,000 units penicillin and 10 mg streptomycin/mL), L-glutamine solution (200 mM) and calcein were purchased from Sigma Aldrich. AlamarBlue™ Cell Viability Reagent was purchased from Invitrogen. 96-well and 6-well cell culture plates were obtained from Corning. Ultra-pure water (H_2O, 18.2 MΩ cm) was obtained from a Millipore Milli-Q Gradient system (Millipore, Bedford, MA). All reagents were obtained from commercial sources and were used without any further purification.

Human hepatoblastoma cell line (HepG2) was purchased from European Collection of Animal Cell Cultures (ECACC Reference No 85011430, Porton Down, UK). Human pancreatic ductal adenocarcinoma cell line (PANC-1) was purchased from Sigma-Aldrich (catalogue number: 87092802). Human breast cancer cells (MCF-7) were obtained from University of Glasgow, Institute of Cancer Sciences.

S1.2. Methods

Synthesis of MOF-808 and MOF-808_act

MOF-808: Zirconyl chloride octahydrate (1.208 g, 3.75 mmol, 3 equivalents) and benzene-1,3,5-tricarboxylic acid (262.7 mg, 1.25 mmol, 1 equivalent) were dissolved in DMF (50 mL). The modulator, acetic acid (50 mL) was added into this mixture, which was sonicated briefly (20 sec). The solution was sealed in a 500 ml glass jar and kept in an oven at 130 °C for 24 h. To remove unreacted chemicals, the nanoparticles were collected by centrifugation (4500 rpm, 15 min), washed by dispersion-centrifugation cycles with DMF (2 × 45 mL) and acetone (5 × 45 mL), and dried in a desiccator for two days to yield MOF-808.

MOF-808_act: 650 mg of MOF-808 was dispersed in a methanol (260 mL) and water (36.4 mL) mixture and stirred at room temperature for 1 day. The nanoparticles were washed by dispersion-centrifugation cycles with methanol (5 × 40 mL) and dried in a desiccator for two days to yield MOF-808_act.

Synthesis of PAAMAM

PAAMAM was synthesized via RAFT polymerization. Mannose acrylamide (1.0 g, 3.6 mmol) and acrylic acid (0.26 g, 3.6 mmol) were added to a pressure resistant vial and dissolved in mixture of DMF/H_2O (70/30 *v*/*v*, 1 mL) and purged with N_2. Next, [[(butylthio)carbonothioyl]thio]propanoic acid (PABTC, 21.4 mg, 0.09 mmol) and 4,4'-azobis (4-cyanovaleric acid) (ACVA, 2.5 mg, 0.009 mmol) were dissolved in a small amount of DMF (0.3 mL) and were added to the reactor vial. The reaction solution was degassed at room temperature for 15 min. The vial was placed in an oil bath at 70 °C to start the polymerisation. The polymer conversion was monitored by ^{1}H NMR spectroscopy and the reaction was stopped when full monomer conversion was obtained (12 h). Finally, the polymer was purified by dialysis against distilled water for 2 days and freeze-dried to obtain a white solid.

Drug Loading

For post-synthetic incorporation of FUDR, CARB, and both FUDR and CARB to MOF-808 or MOF-808_act, 120 mg of each MOF was dispersed in methanol (24 mL). In a separate vial 120 mg of FUDR, 120 mg of CARB or both FUDR and CARB (120 mg of each) were dissolved in 24 mL of methanol containing 6.72 mL water and mixed with MOF-808 or MOF-808_act after being sonicated for 15 min at room temperature to obtain FUDR@MOF-808, CARB@MOF-808 and (FUDR+CARB)@MOF-808, or FUDR@MOF-808_act CARB@MOF-808_act and (FUDR+CARB)@MOF-808_act, respectively. The drug solutions were stirred at room temperature for one day and the obtained nanoparticles were washed by dispersion-centrifugation cycles with methanol (5 × 40 mL) and dried in a desiccator for two days.

PAAMAM coating of MOFs

For glycopolymer (PAAMAM) coating of MOF-808, the MOF nanoparticles (75 mg) were dispersed in 15 mL of methanol. In a separate vial, the glycopolymer (7.5 mg) was dissolved in 15 mL of methanol. Then, the solutions were mixed and stirred at room temperature for one day. The coated

samples were washed by dispersion-centrifugation cycles with methanol (5 × 30 mL) and dried in a desiccator for two days. The procedure was repeated with MOF-808_act, (FUDR+CARB)@MOF-808 and (FUDR+CARB)@MOF-808_act.

Characterization

^{1}H Nuclear Magnetic Resonance Spectroscopy (NMR): NMR spectra were collected on either a Bruker AVIII 400 MHz spectrometer, a Bruker AVI 500 MHz spectrometer, or a Bruker DPX-300 spectrometer (glycopolymer) and referenced to residual solvent peaks. Measurements were carried out at room temperature.

Gel permeation chromatography (GPC): GPC measurements of the glycopolymer were carried out on an Agilent 1260 Infinity II-MDS instrument with two PLAquagel Mixed-M columns in H_2O/MeOH (80/20) with 0.1 M $NaNO_3$ equipped with the following detectors: a refractive index (RI), viscometer (VS), light scattering (LS), and variable wavelength detector (VWD). The instrument was calibrated with narrow linear poly(ethylene glycol) standards. All samples were passed through 0.2 µm nylon filters prior to GPC measurements.

Powder X-Ray Diffraction (PXRD): PXRD measurements were performed at 298 K using a Rigaku MiniFlex benchtop diffractometer equipped with a Cu sealed tube X-ray source where the CuKα wavelength = 1.4505 Å.

Thermogravimetric Analysis (TGA): Measurements were conducted using a TA Instruments Q500 Thermogravimetric Analyser. Data were collected from room temperature to 800 °C with a heating rate of 10°C min^{-1} under an air atmosphere.

Fourier Transform Infrared Spectroscopy (FTIR): IR spectra of solids were collected at a range of 400-4000 cm^{-1} using an attenuated total reflection (ATR)-FTIR spectrometer (Thermoscientific Nicolet Summit FTIR spectrometer with an Everest ATR) with an accompanying OMNIC software.

Scanning Electron Microscopy (SEM): The powder samples were coated with Au/Pd for 50 seconds using Polaron SC7640 sputter coater and imaged using a Carl Zeiss Sigma Variable Pressure Analytical SEM with Oxford Microanalysis. Particle size distribution was measured manually using ImageJ software version 1.52a (National Institutes of Health, Bethesda, MD, USA) by randomly selecting 100 particles from an SEM image.

Dynamic Light Scattering (DLS)/Zeta potential: The hydrodynamic size, polydispersity index (PDI) and zeta potential of MOFs in water were determined using a Malvern Dynamic Light Scattering (DLS)

with a Zetasizer Nano ZS potential analyser equipped with Non-Invasive Backscatter optics (NIBS) and a 50 mW laser at λ = 633 nm. The samples were prepared with a concentration of 0.25 mg mL^{-1} by sonication for 20 min prior to measurement. The pH of the solutions was adjusted to about 7.4 to eliminate pH effect on zeta potential of the nanoparticles. All measurements were carried out in triplicate.

Gas Uptake and Pore-Size Distribution: N_2 adsorption isotherms were recorded on a Quantachrome Autosorb iQ gas sorption analyser at 77 K. Samples were degassed under vacuum at 120 °C for 20 h by using an internal turbo pump. BET surface areas were calculated from the isotherms using the Micropore BET Assistant in the Quantachrome ASiQwin operating software. Pore size distributions were calculated using N_2 at 77 K on carbon, slit/cylinder/sphere pore QSDFT, adsorption model within the same software (version 3.01).

UV/Vis Spectroscopy: UV/Vis absorbance spectra were recorded on a Shimadzu UV-1800 and analyses of data were performed using the software UVProbe (version 2.51).

FUDR Determination: 2 mg of the prepared formulations were dispersed in 10 mL of PBS (1x, pH 7.4) and stirred for 4 days at room temperature. The degradation solutions were lyophilized on a Christ Alpha 2-4 LO plus freeze dryer to evaporate PBS and then, the degradation product was dissolved in methanol (4 mL) and centrifuged. The supernatant was filtered through 0.2 µm PTFE filters prior to analysis by HPLC and was analysed by a Shimadzu reverse-phase HPLC (RP-HPLC) system equipped with Shimadzu LC-20AT pumps, a Shimadzu SIL-20A autosampler and a Shimadzu SPD-20A UV-Vis detector (monitoring at λ = 268 nm for FUDR and λ = 205 nm for benzene-1,3,5-tricarboxylic acid) using a C18 Kinetex® reverse-phase LC column (250 × 4.6 mm, 5 µm). Collection of the chromatograms was completed using a mobile phase of water:acetonitrile (95:5% with 0.1% trifluoroacetic acid) for 20 min at a flow rate of 1 mL min^{-1}. The drug concentration was calculated according to a calibration curve of FUDR prepared in methanol. FUDR loading content was reported from the equation below (*n* =3):

$$FUDR\ (\%, w/w) = \frac{weight\ of\ loaded\ drug}{weight\ of\ drug\ loaded\ MOFs}\ x\ 100\%$$

(1)

CARB Determination: Samples were submitted to MEDAC Ltd (Surrey, UK) for inductively coupled plasma optical emission spectrometry (ICP-OES) analysis of Pt content of CARB-loaded samples. CARB loading was calculated from the Pt results considering molecular weight of CARB.

Cell culture

MCF-7 human breast cancer and PANC-1 human pancreatic ductal adenocarcinoma cells were cultured in Roswell Park Memorial Institute (RPMI) 1640 medium supplemented with 10% (*v/v*) FBS, 1% (*v/v*) penicillin (100 U/mL) streptomycin (100 µg/mL) and 2 mM L-glutamine. HepG2 human hepatoblastoma cells were cultured in Minimum Essential Media (MEM) supplemented with 10% (*v/v*) FBS, 1% (*v/v*) penicillin (100 U/mL) streptomycin (100 µg/mL), 2 mM L-glutamine and 1% (*v/v*) non-essential amino acid (NEAA) solution. All cells were grown in a humidified incubator at 37°C in 5% CO_2 and passaged every 2–3 days.

Cytotoxicity assay

MCF-7 and PANC-1 cells at a density of 5000 cells/well and HepG2 cells at a density of 8000 cells/well were seeded into 96-well plates separately and incubated at 37°C in a 5% CO_2 atmosphere. On the second day, they were subjected to the samples at different concentrations with fresh culture medium and incubated for additional 24 h or 72 h. Cells without any treatment were employed as positive controls. At the end of incubation, Alamar Blue reagent was introduced to each well according to the manufacturer's instructions (10% *v/v*) and the cells were further incubated at 37°C in 5% CO_2 for 4 h. Fluorescence reading was performed under 557/10 nm excitation and 593/10 nm emission using a CLARIOstar microplate reader (BMG Labtech, Ortenberg, Germany). The relative cell viability was calculated in reference to the untreated control cells using the following equation:

$$\text{cell viability (\%)} = \left[\frac{\text{sample fluorescence intensity}}{\text{control fluorescence intensity}}\right] \text{x } 100 \qquad (2)$$

The average cell viability was calculated using three different passages of cells with 3 repeats with each (*n* = 3x3).

Intracellular Uptake of MOFs

Calcein Loading of MOFs: 15 mg of MOF was dispersed in 7.5 mL of methanol and subsequently mixed with 7.5 mL of methanolic solution of calcein (2 mg mL^{-1}). The loading solution was left to stir at room temperature for 1 day and the calcein loaded MOFs were collected by centrifugation (4500 rpm, 20 min) and wash through dispersion centrifugation cycles with fresh MeOH until the supernatant solution remains colourless (around 5 times), and then dried in a desiccator for two days.

Flow Cytometry Analysis: Intracellular uptake of MOF nanoparticles was determined using a BD LSRII running FACS-Diva software (BD Bioscience) equipped with Alexa Fluor 488 (530/30) and PE-Cy5 (660/20) filters. MCF-7, PANC-1 and HepG2 cells were seeded at a density of 1×10^6 cell/well into 6-well plates and incubated with the calcein loaded samples at 50 µg mL^{-1} concentration at 37°C in a 5% CO_2. The second day, cells were detached by trypsin-EDTA and resuspended in flow cytometry buffer (DPBS with 1% FBS and 2mM EDTA) after being collected by centrifugation. Free calcein was used as a positive control. SYTOX AAdvanced dead cell stain (S10274 A) was used to determine dead cells after treatment of cells with MOFs (Calcein λ_{ex} 488 nm, λ_{em} 530 ± 30 nm; SYTOX AAdvanced λ_{ex} 561 nm, λ_{em} 660 ± 20 nm). All experiments were repeated three times and data are reported as the average. The analysis of data was performed using FCS Express software (version 6).

Statistical analysis

Statistical analysis of data was carried out using one-way ANOVA with Tukey's multiple comparison test of GraphPad Prism 9 software package (GraphPad Software, Inc., USA). $p < 0.05$ was considered statistically significant. All data were presented as mean ± standard deviation (SD).

S2. MOF Synthesis

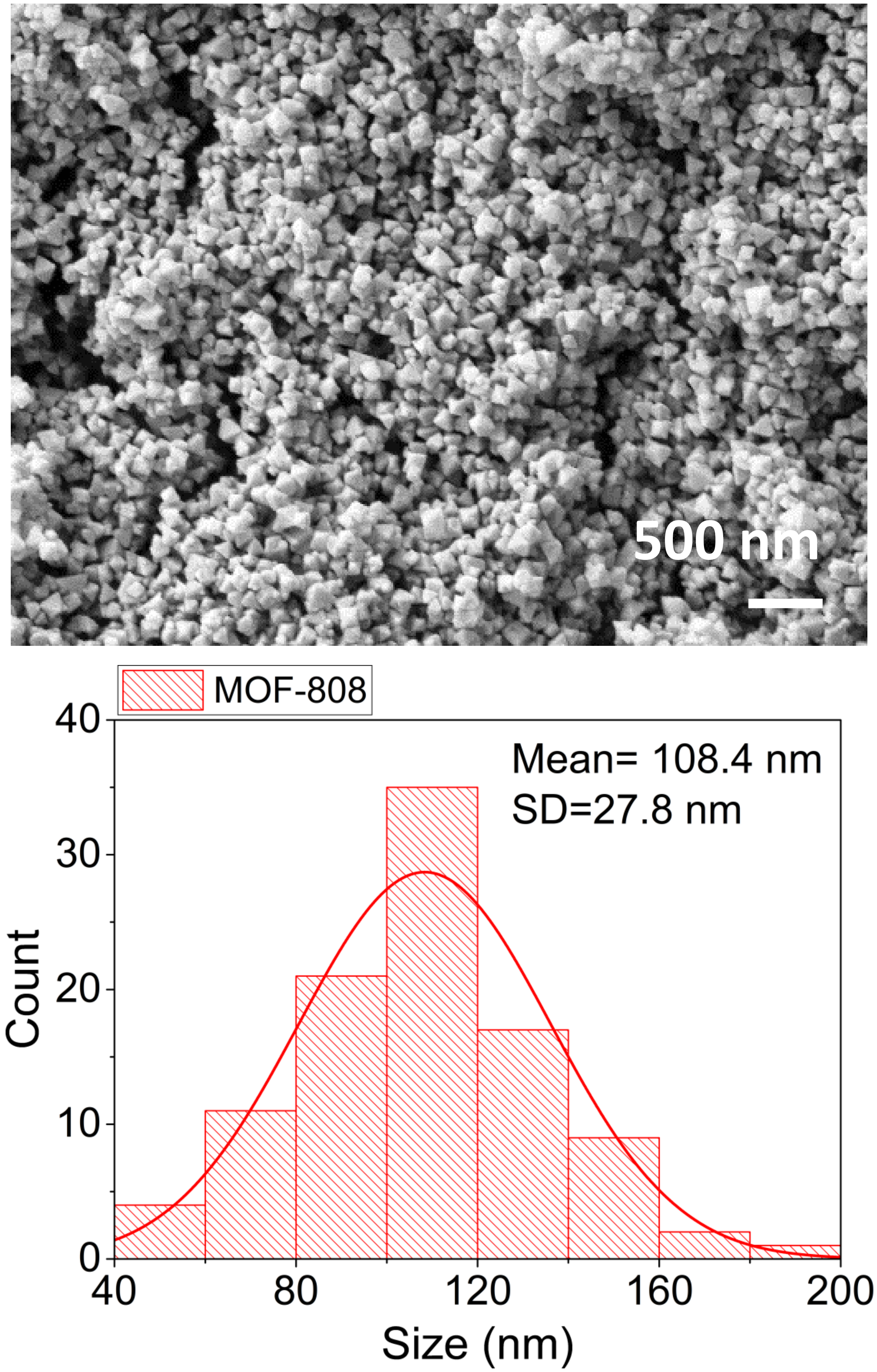

Figure S1. SEM image and particle size distribution histograms of MOF-808.

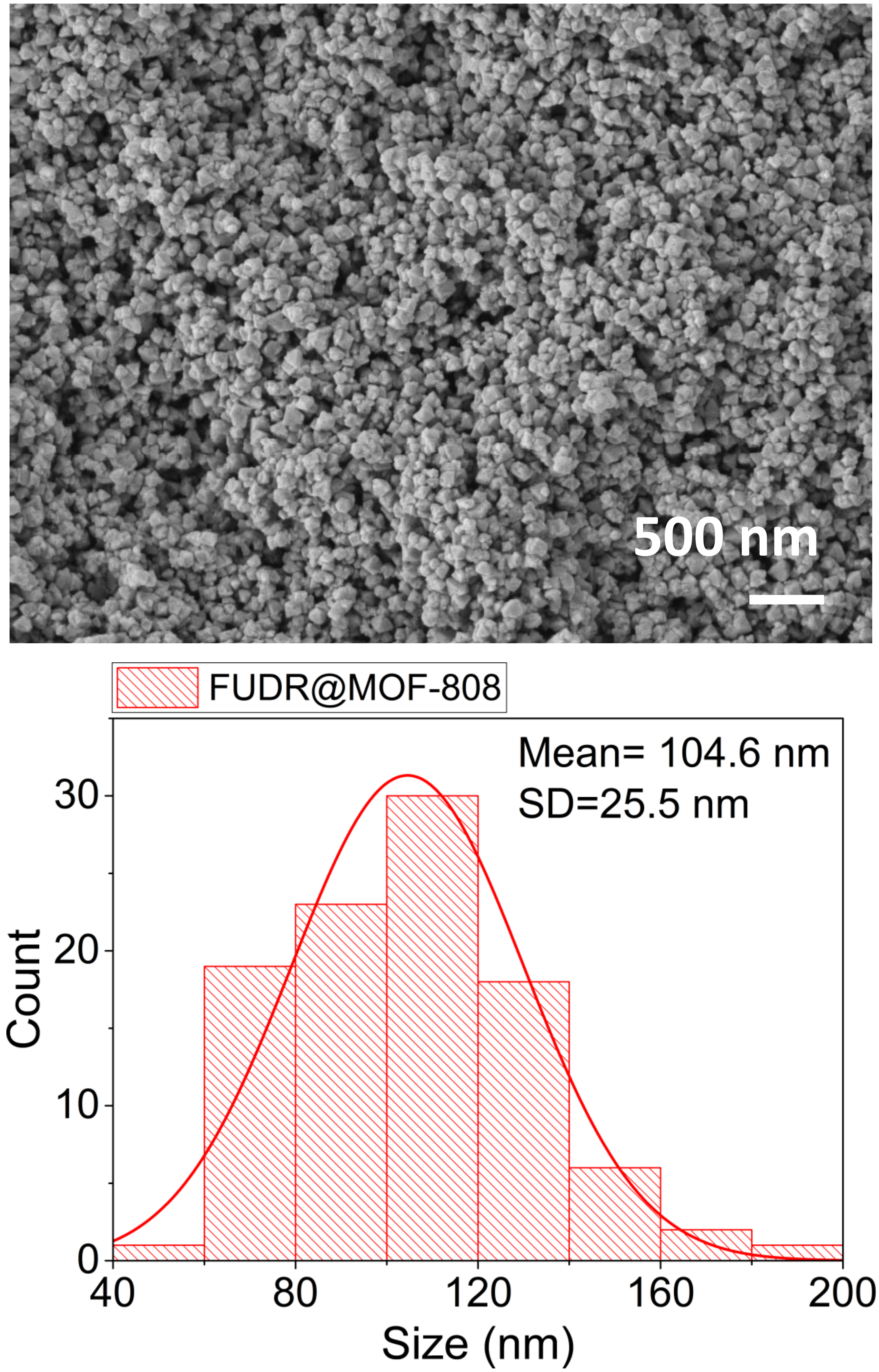

Figure S2. SEM image and particle size distribution histograms of FUDR@MOF-808.

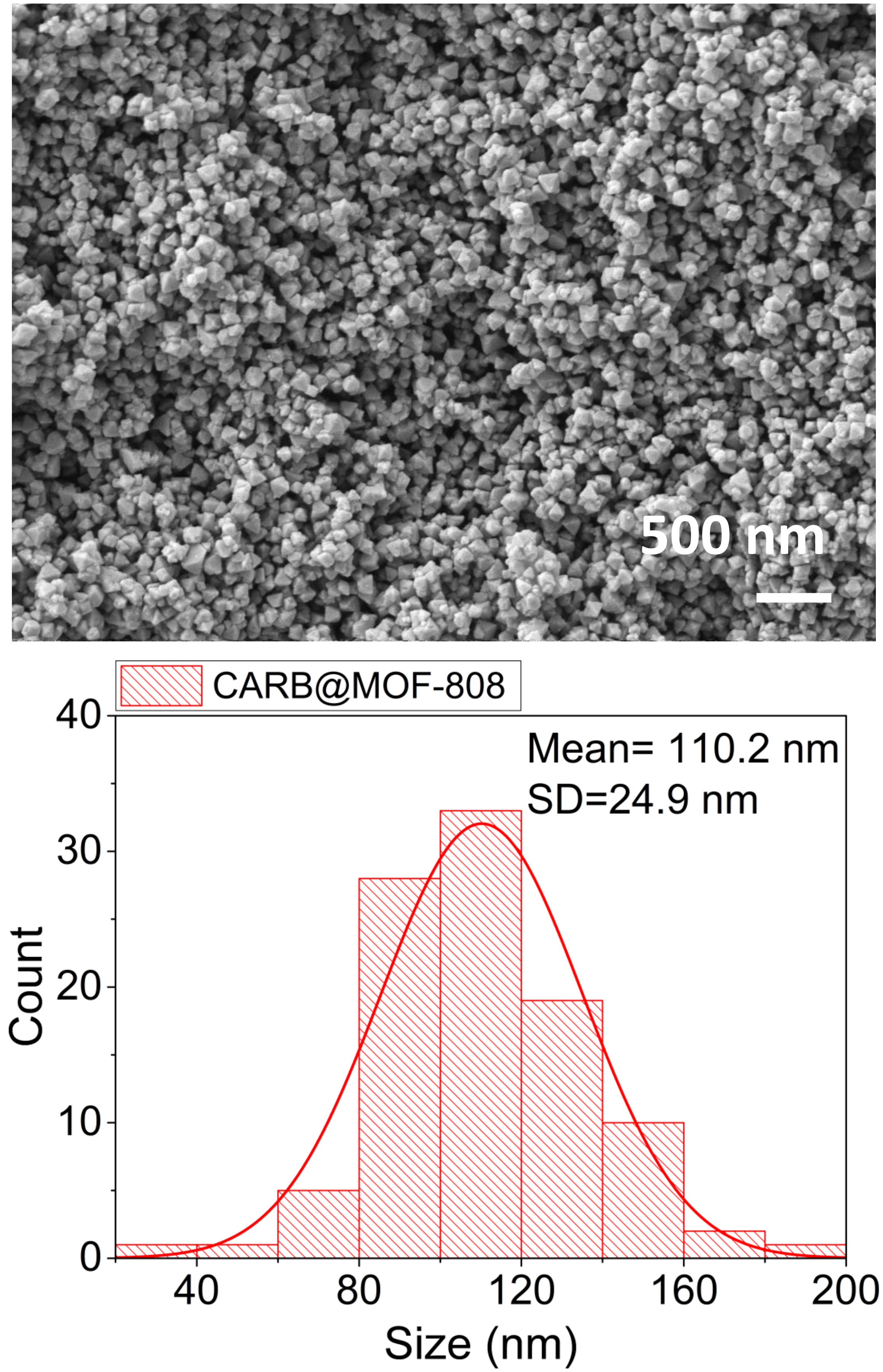

Figure S3. SEM image and particle size distribution histograms of CARB@MOF-808.

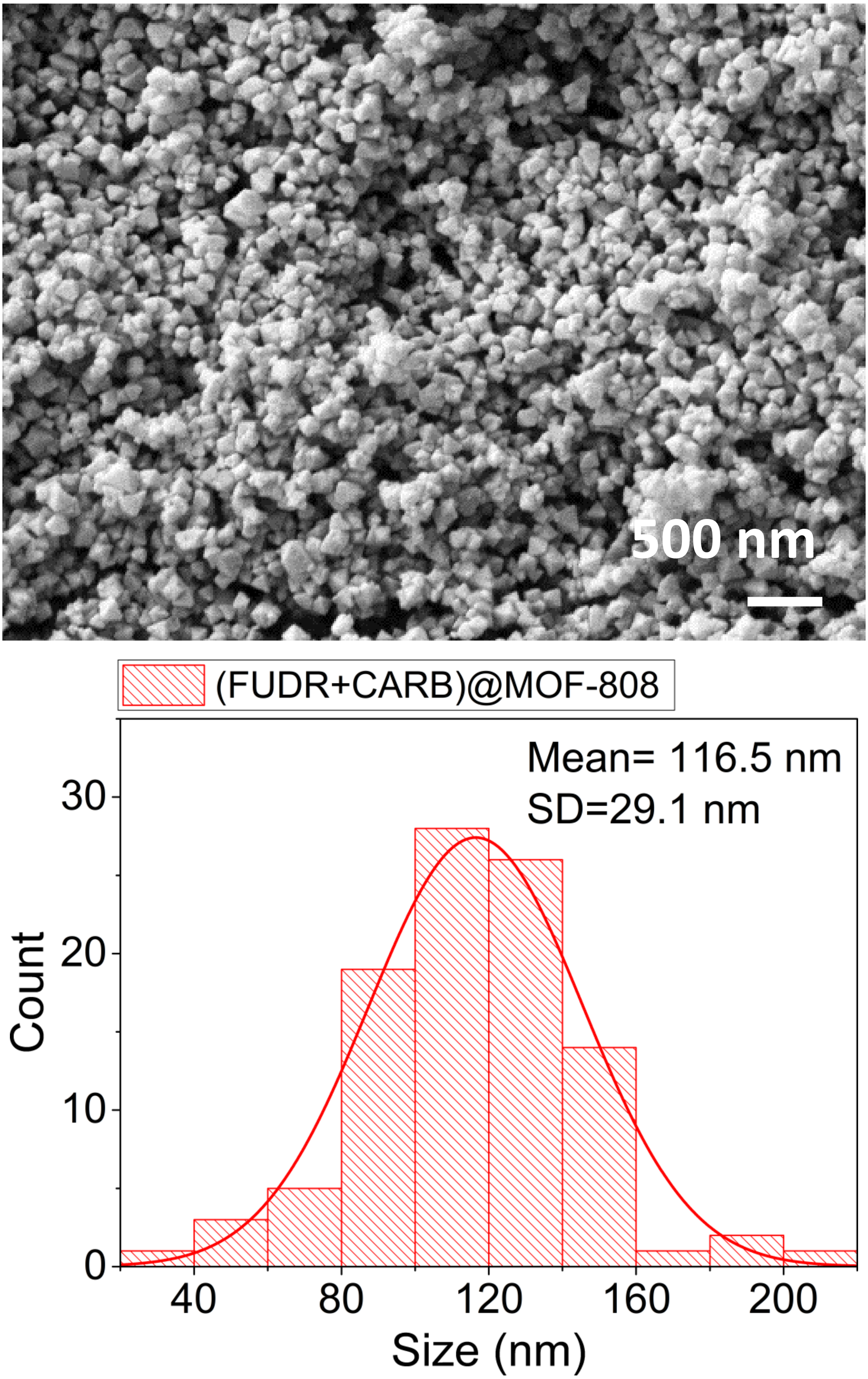

Figure S4. SEM image and particle size distribution histograms of (FUDR+CARB)@MOF-808.

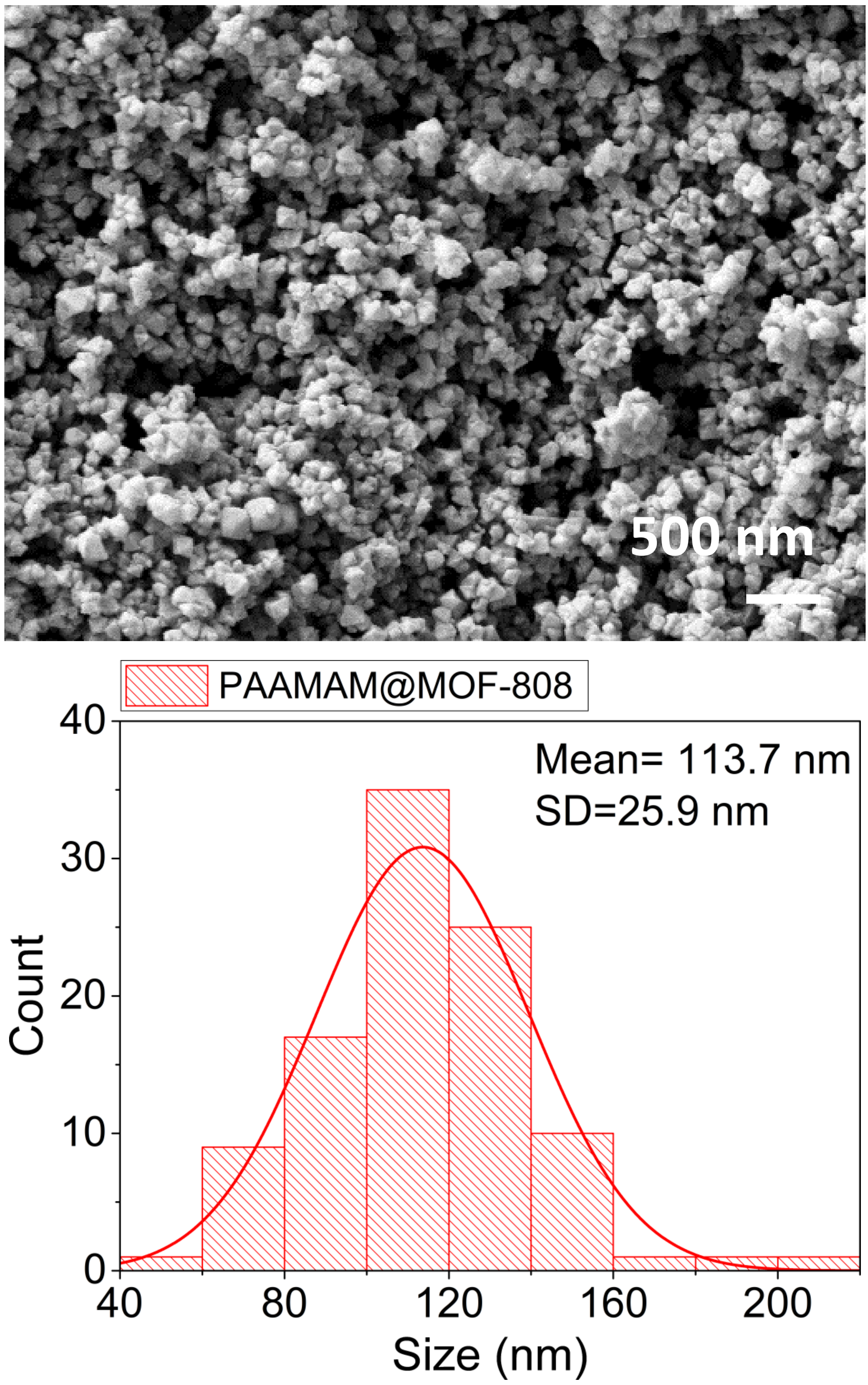

Figure S5. SEM image and particle size distribution histograms of PAAMAM@MOF-808.

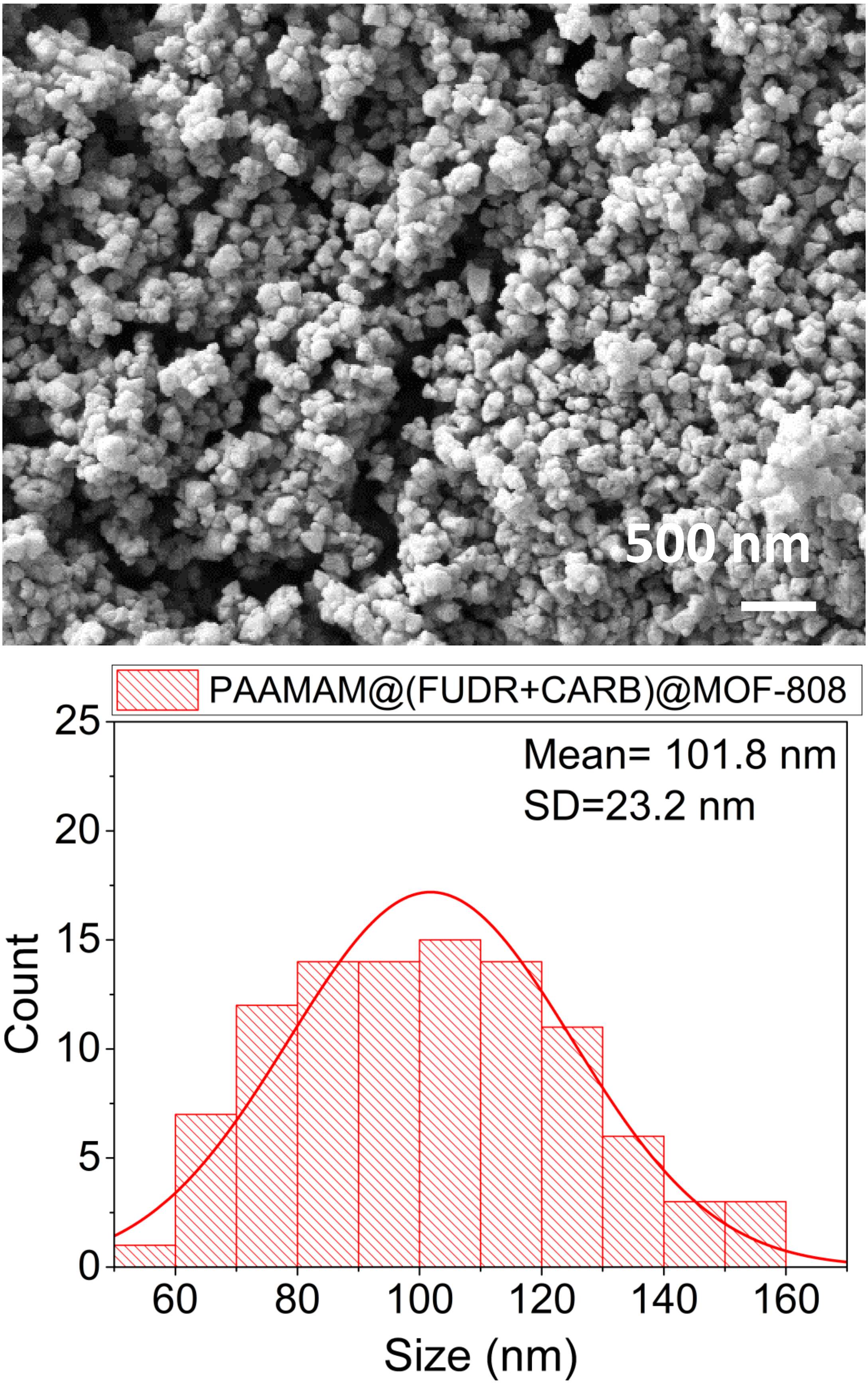

Figure S6. SEM image and particle size distribution histograms of PAAMAM@(FUDR+CARB)@MOF-808.

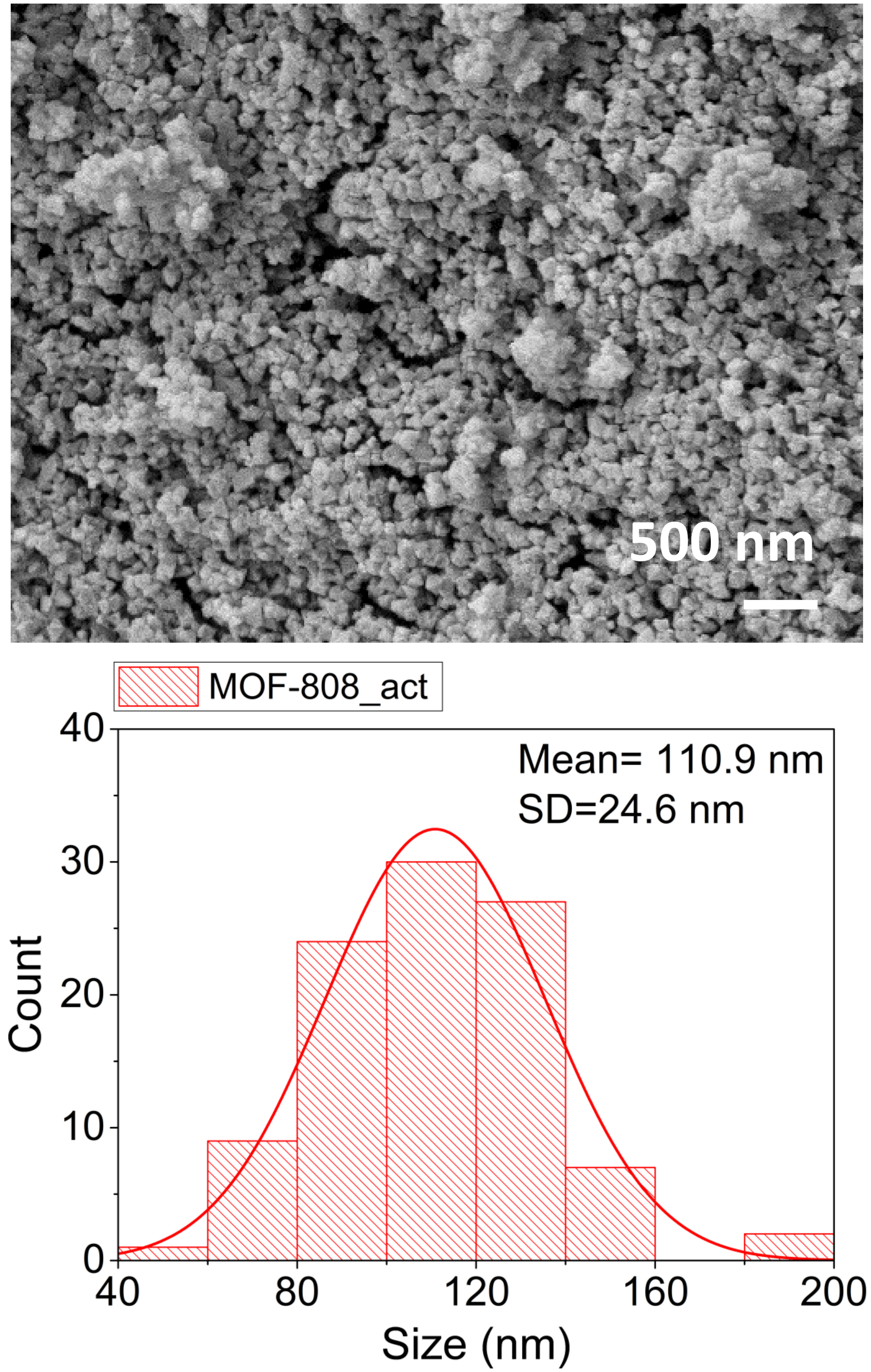

Figure S7. SEM image and particle size distribution histograms of MOF-808_act.

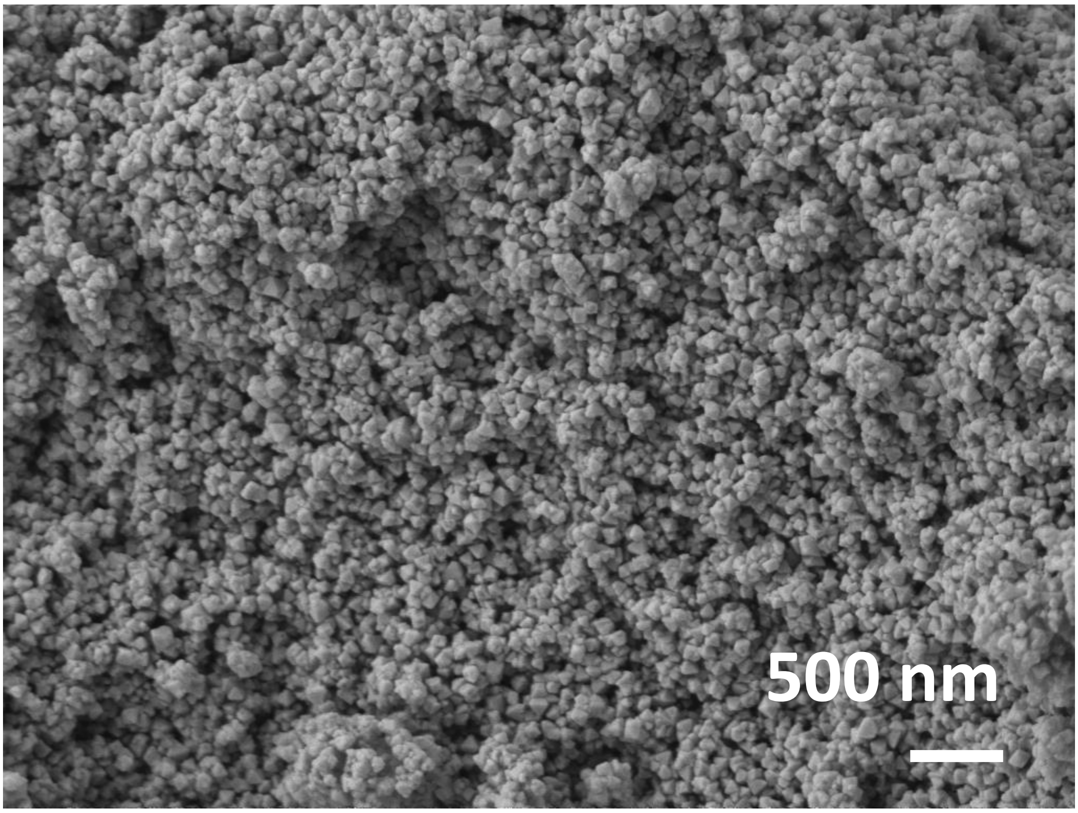

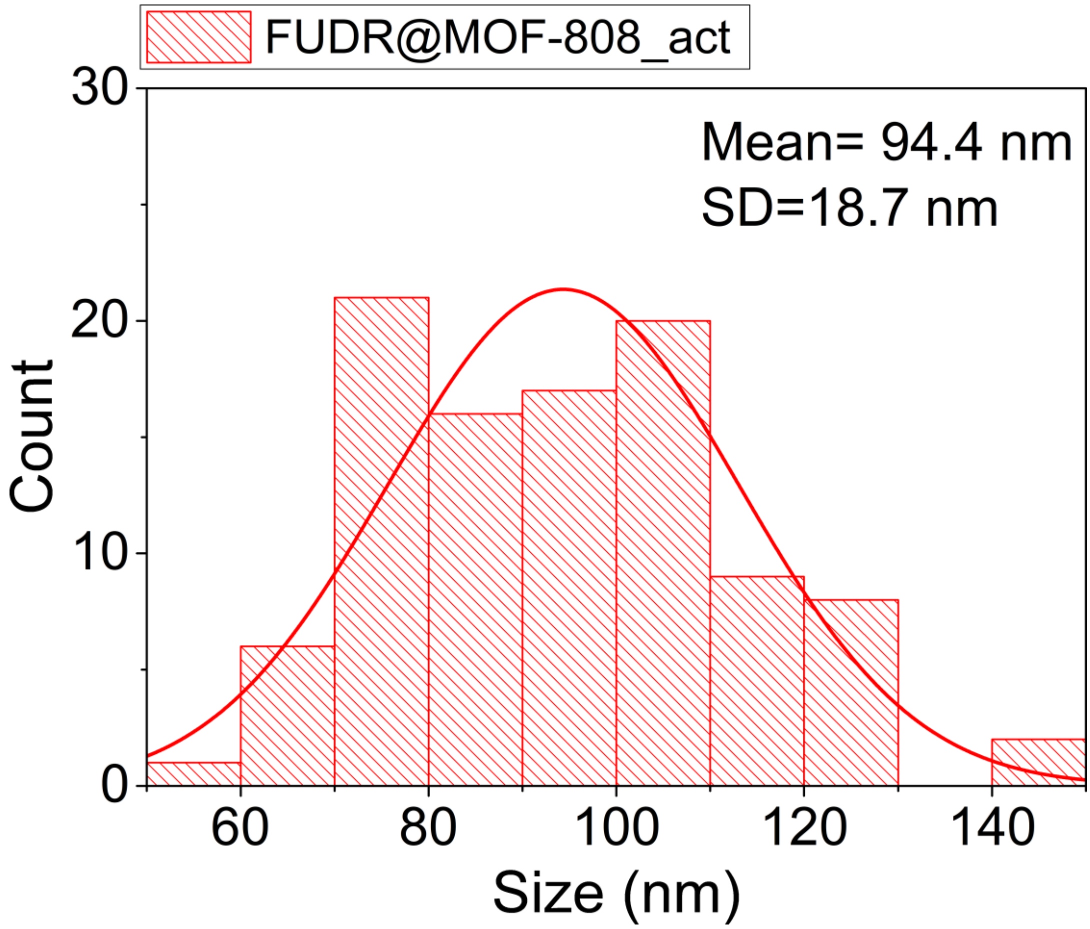

Figure S8. SEM image and particle size distribution histograms of FUDR@MOF-808_act.

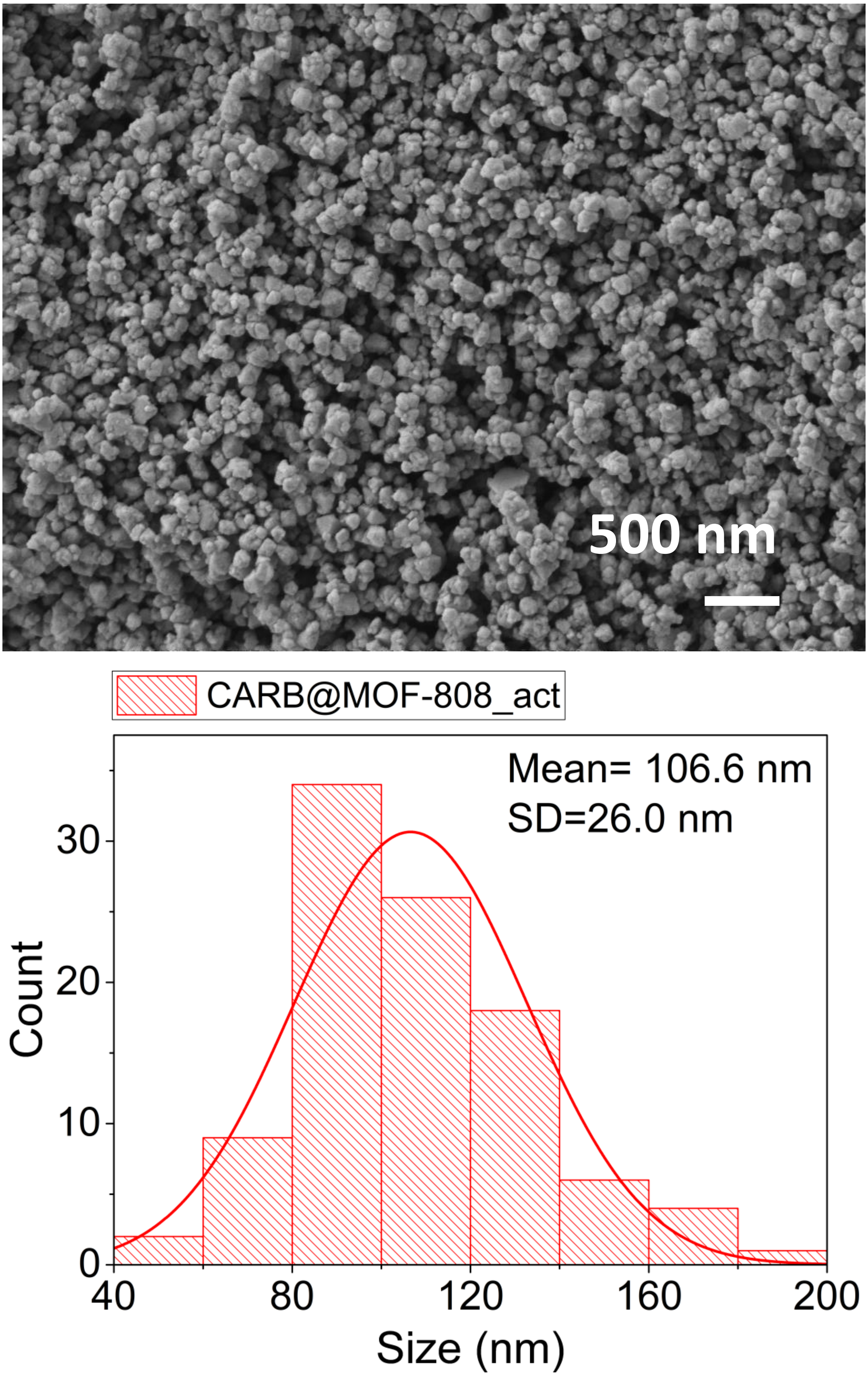

Figure S9. SEM image and particle size distribution histograms of CARB@MOF-808_act.

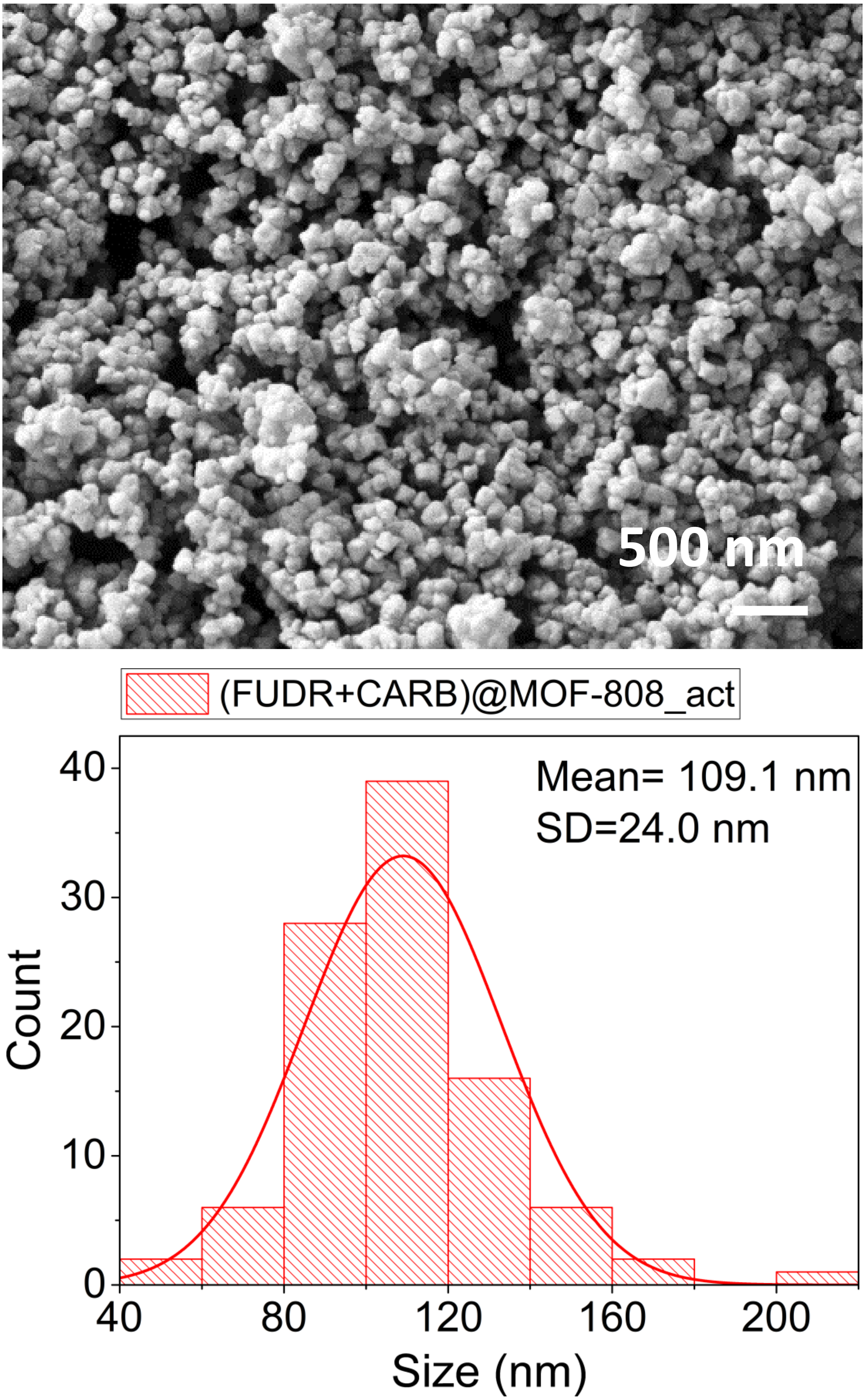

Figure S10. SEM image and particle size distribution histograms of (FUDR+CARB)@MOF-808_act.

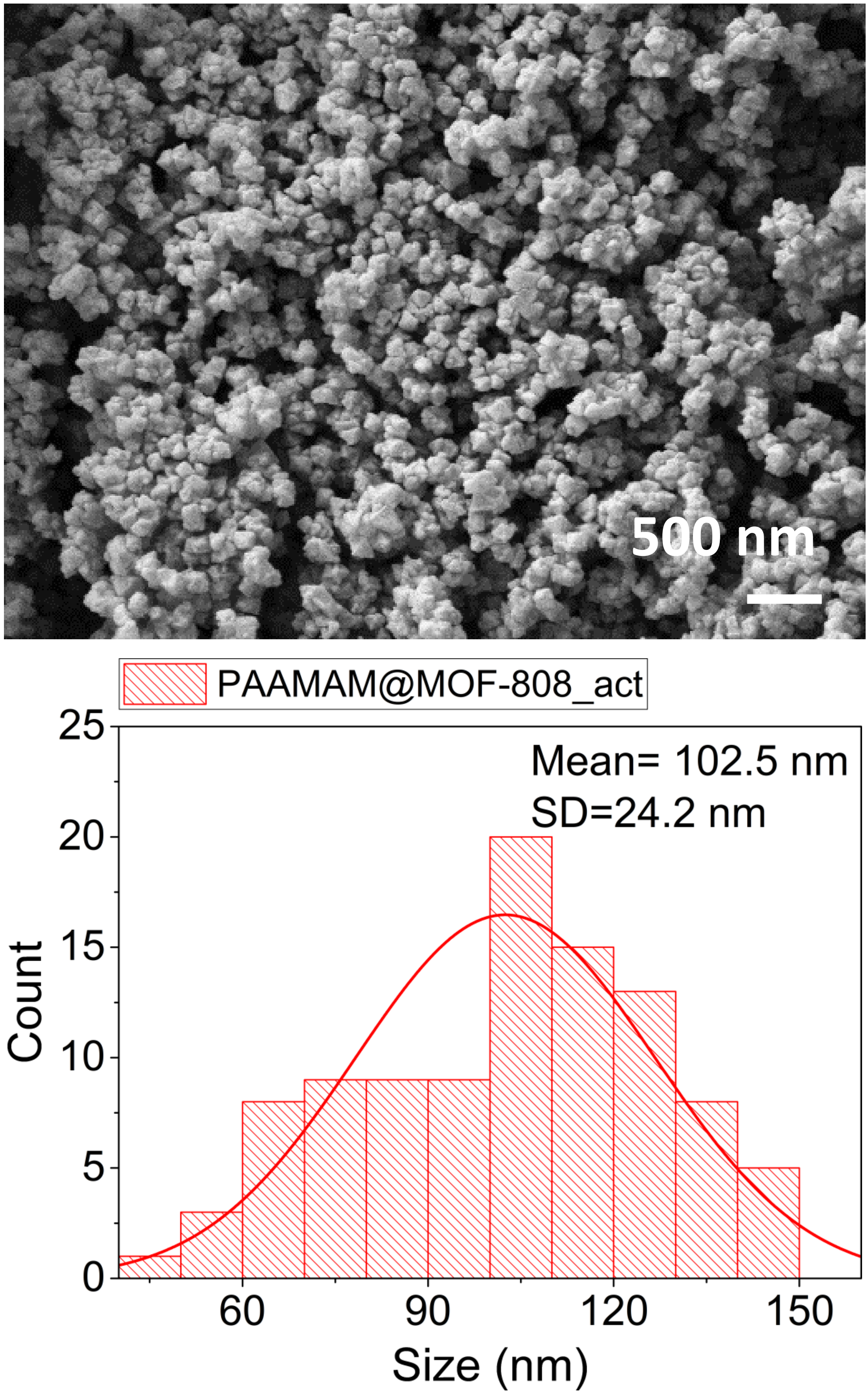

Figure S11. SEM image and particle size distribution histograms of PAAMAM@MOF-808_act.

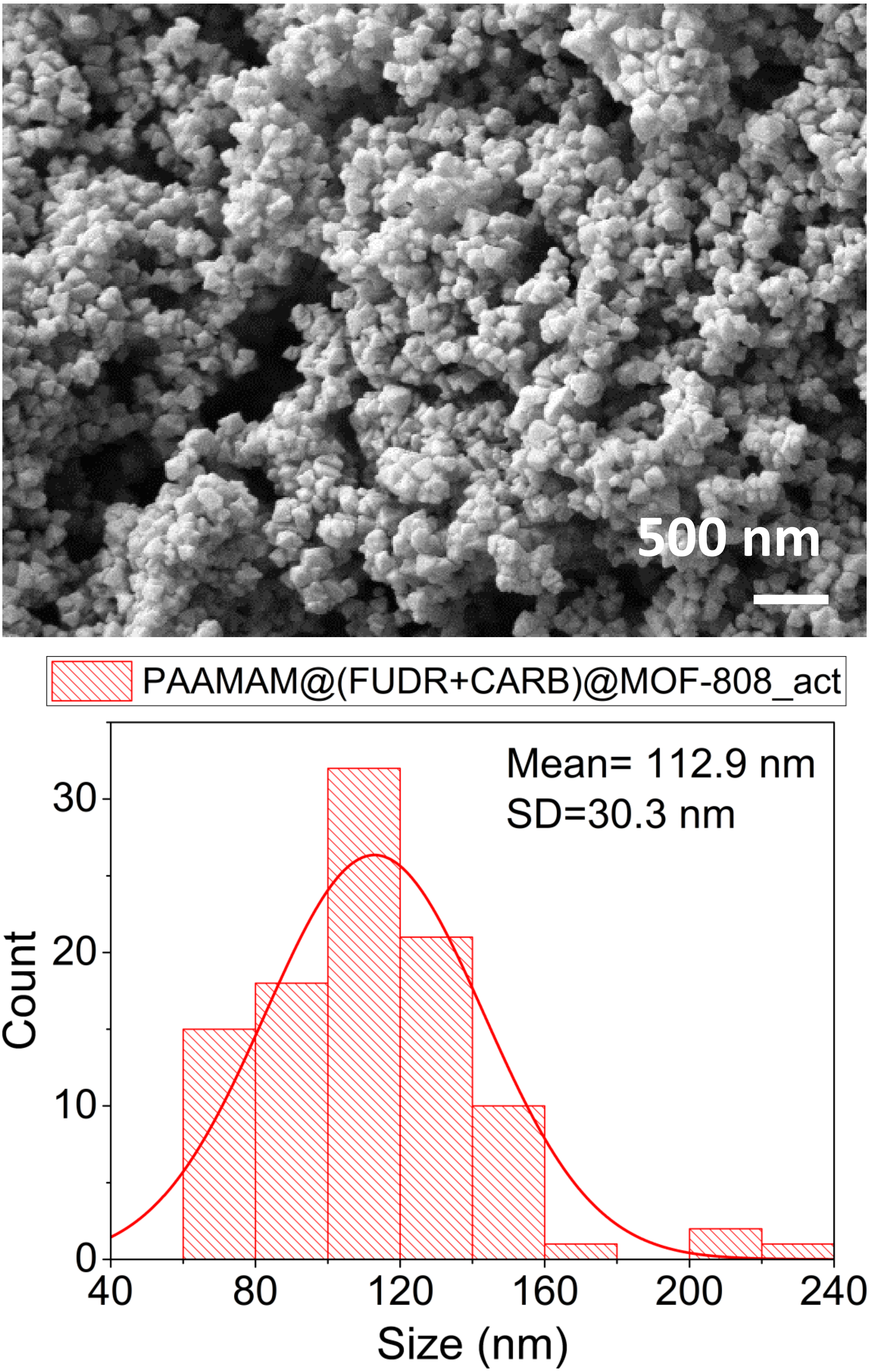

Figure S12. SEM image and particle size distribution histograms of PAAMAM@(FUDR+CARB)@MOF-808_ act.

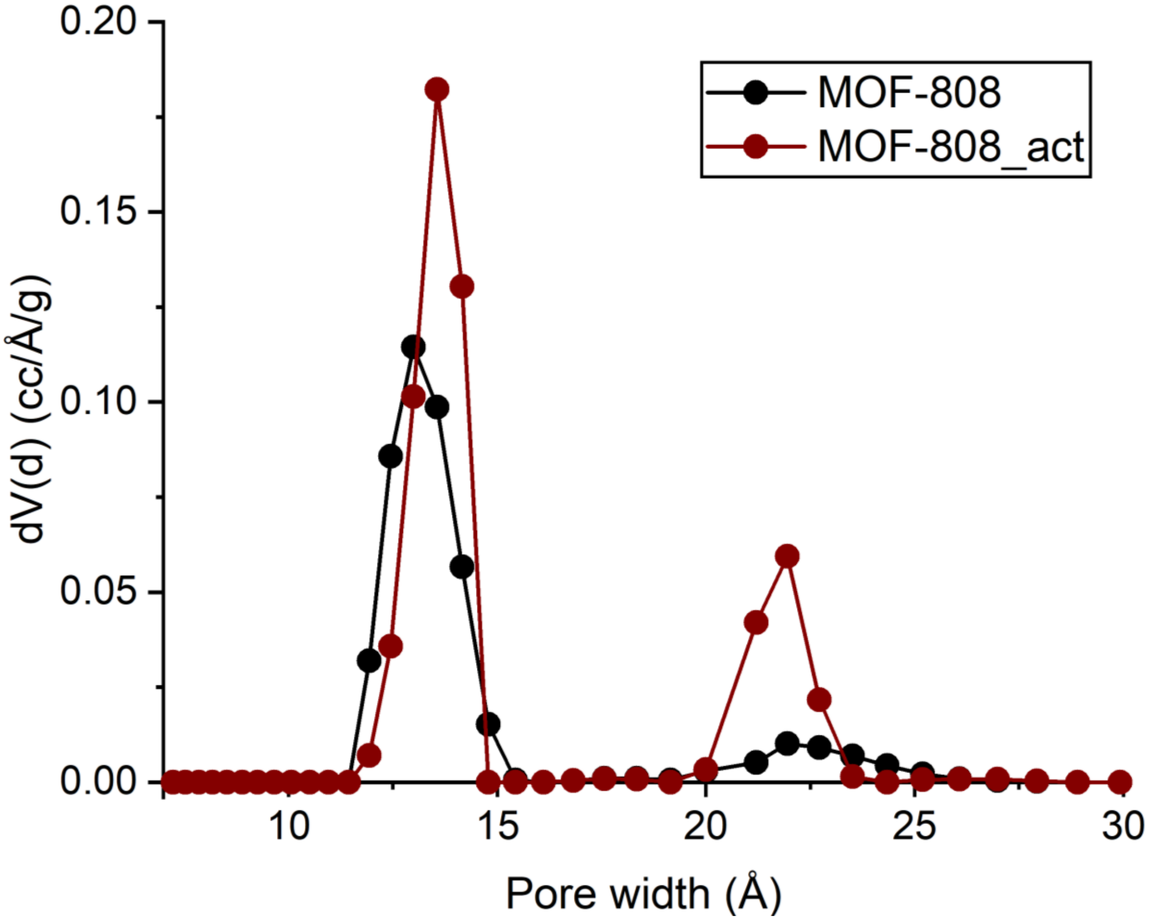

Figure S13. Comparison of pore size distributions (N_2 at 77 K on carbon, slit/cylinder/sphere pores, QSDFT, adsorption model) for MOF-808 and MOF-808_act, calculated from the corresponding N_2 adsorption isotherms in Figure 2c.

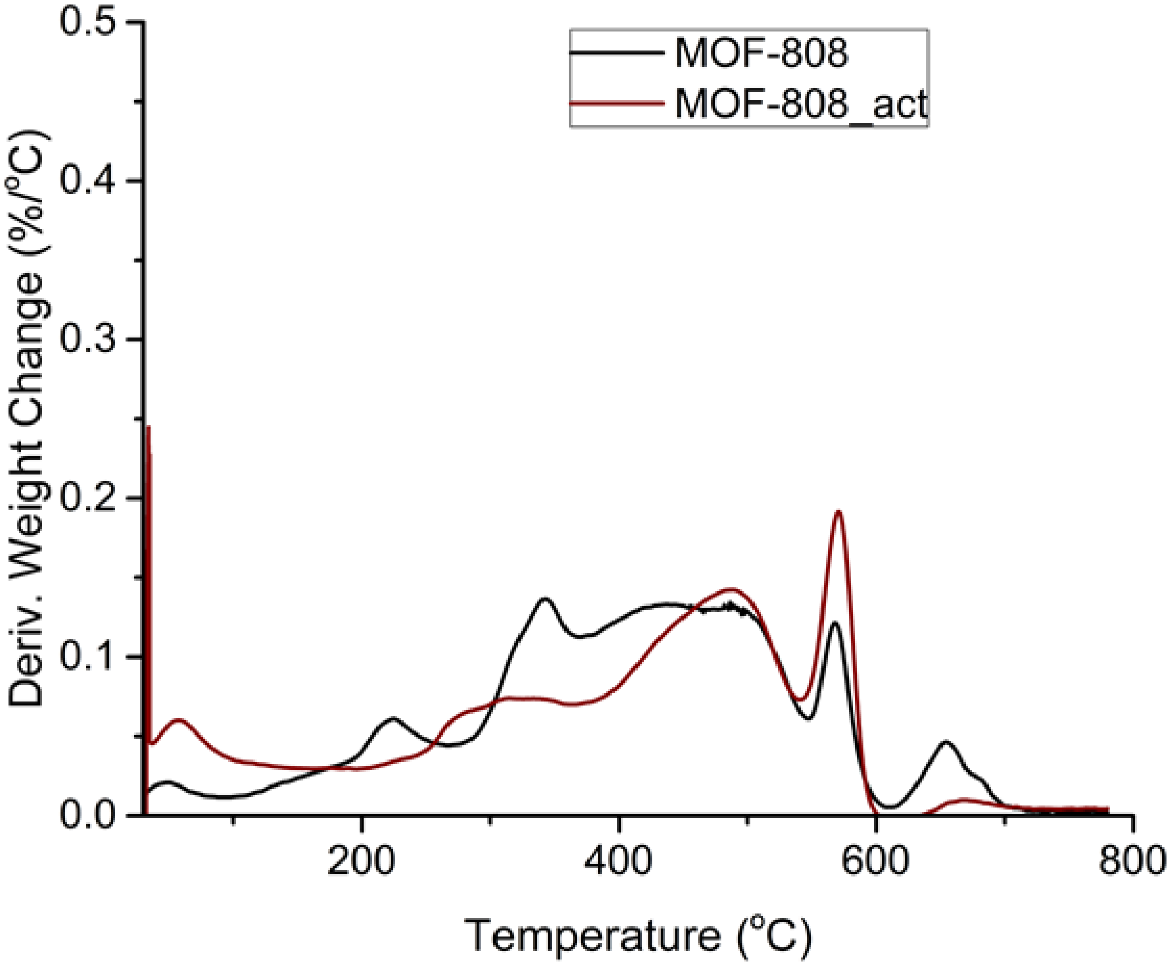

Figure S14. TGA traces of MOF-808 and MOF-808_act plotted as the derivative weight change (%/°C) vs temperature. Derivative calculated from the trace in Figure 2d.

S3. Glycopolymer Synthesis and Characterization

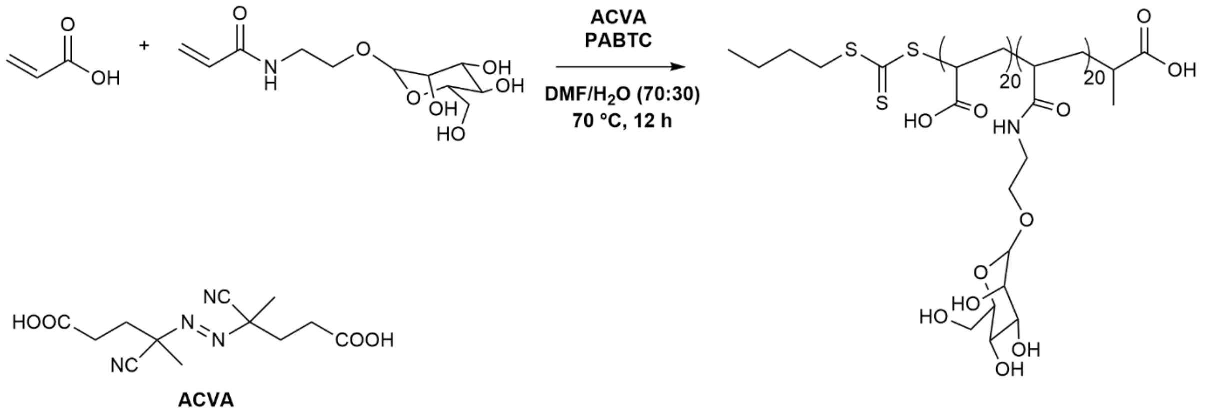

Scheme S1. Reaction scheme for the RAFT copolymerisation of acrylic acid and mannose acrylamide monomers to obtain poly(Acrylic Acid-Mannose Acrylamide).

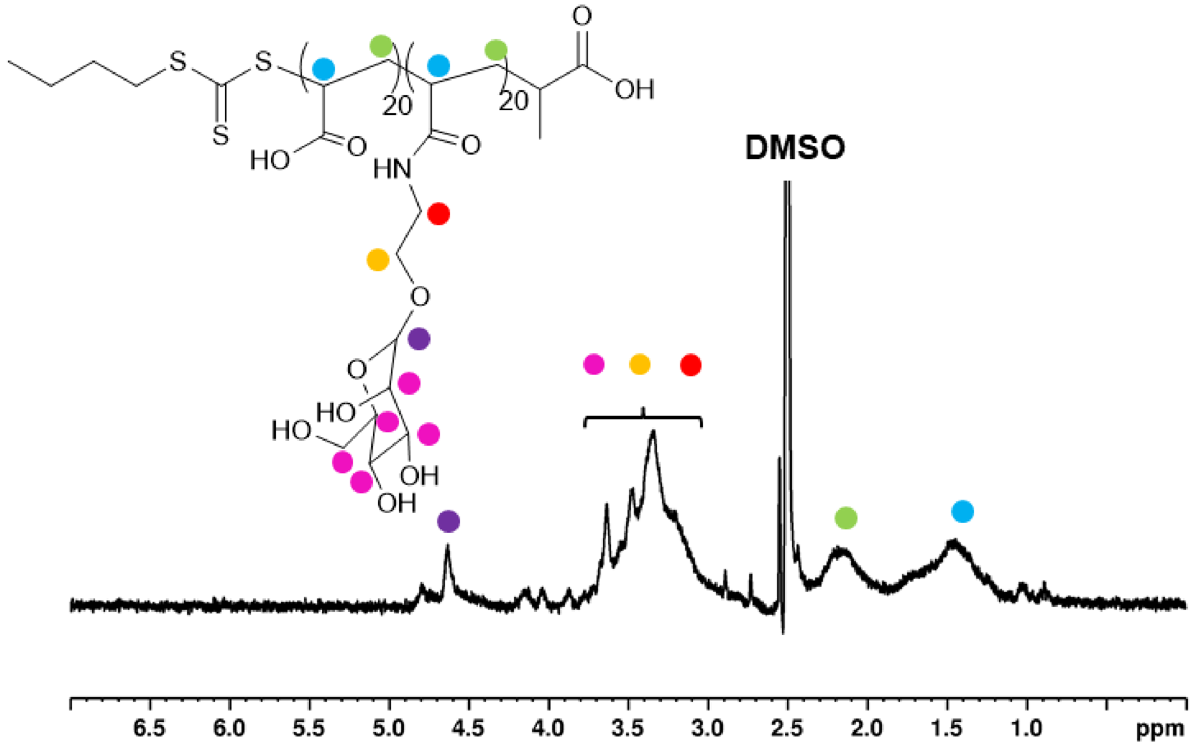

Figure S15. ^1H NMR spectroscopy of poly(Acrylic Acid-Mannose Acrylamide) in DMSO-d_6 at >99% conversion.

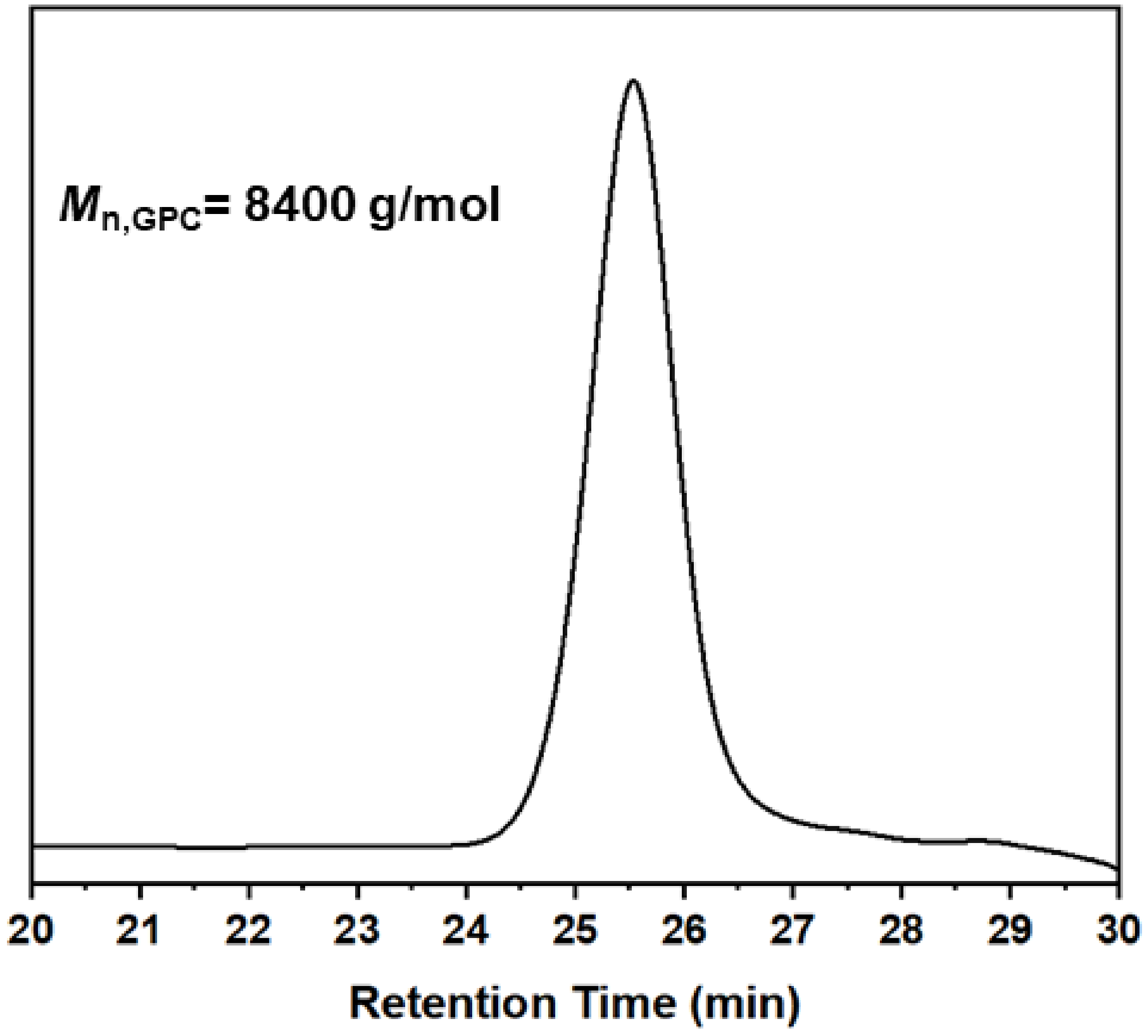

Figure S16. GPC trace of poly(Acrylic Acid-Mannose Acrylamide) in H_2O/MeOH (80/20) with 0.1 M $NaNO_3$.

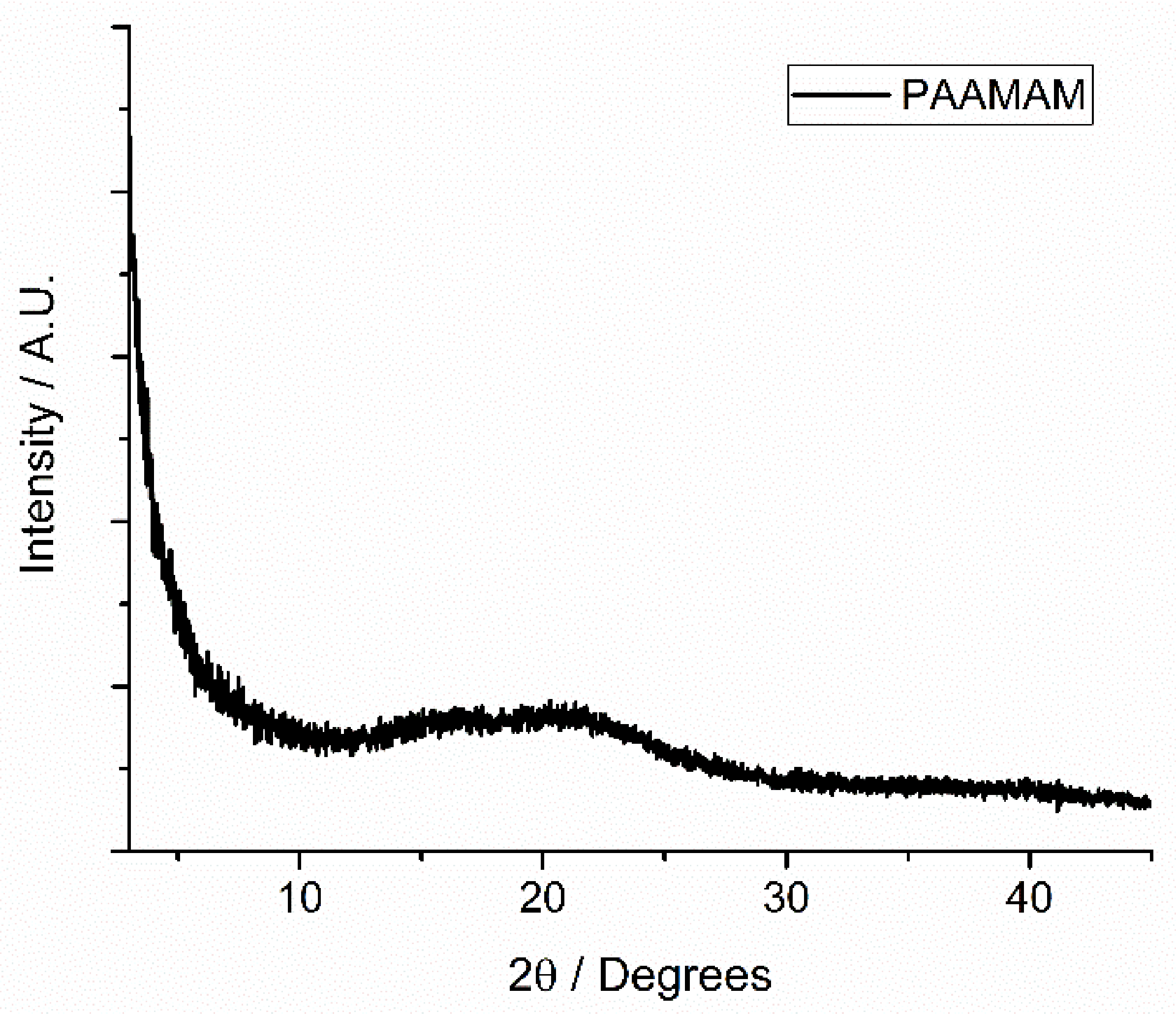

Figure S17. PXRD pattern of the amorphous PAAMAM glycopolymer.

S4. Drug-Loading

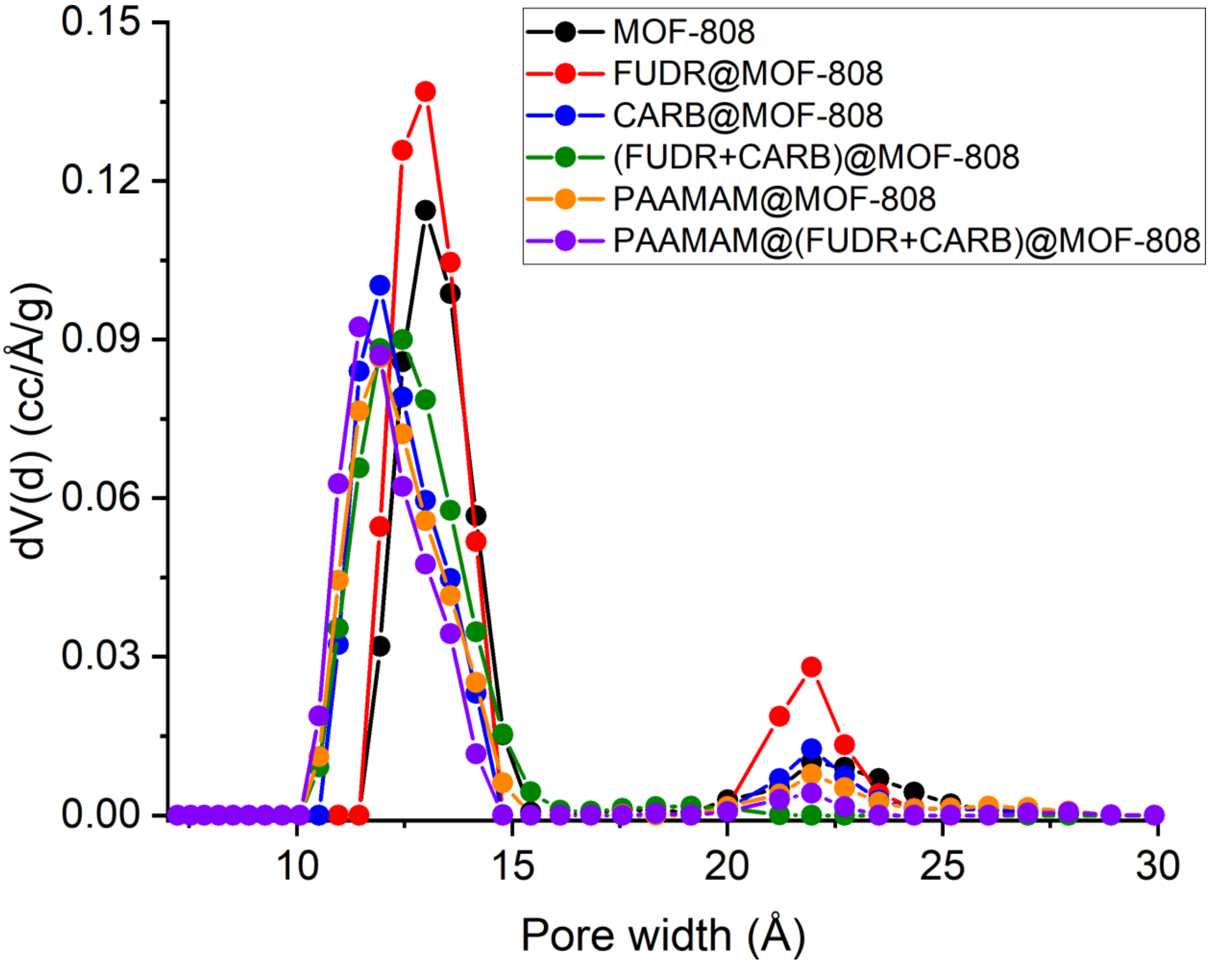

Figure S18. Pore size distribution (N_2 at 77 K on carbon, slit/cylinder/sphere pores, QSDFT, adsorption model) for MOF-808 and its functionalized derivatives calculated from the corresponding N_2 adsorption isotherms in Figure 3c.

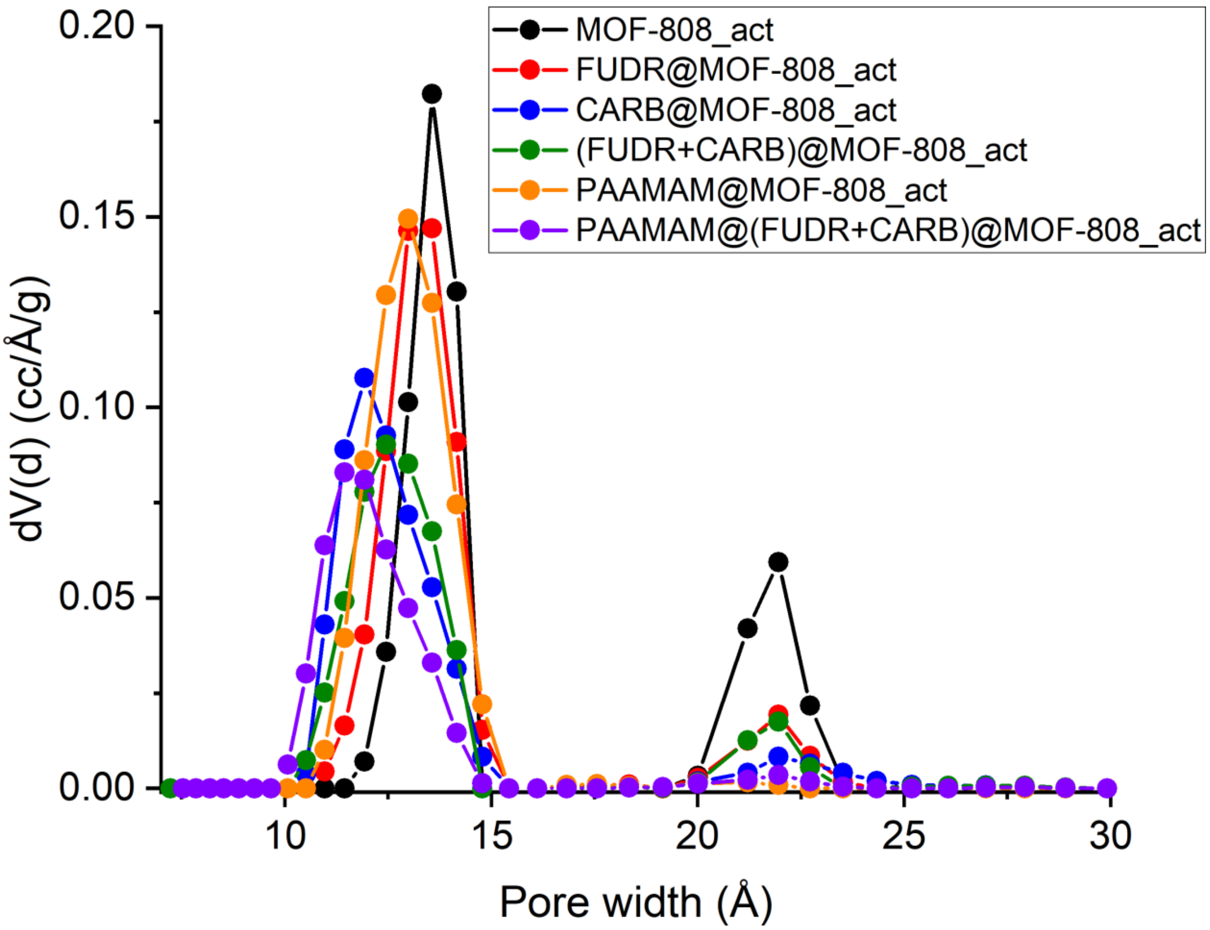

Figure S19. Pore size distribution (N_2 at 77 K on carbon, slit/cylinder/sphere pores, QSDFT, adsorption model) for MOF-808_act and its functionalized derivatives calculated from the corresponding N_2 adsorption isotherms in Figure 3d.

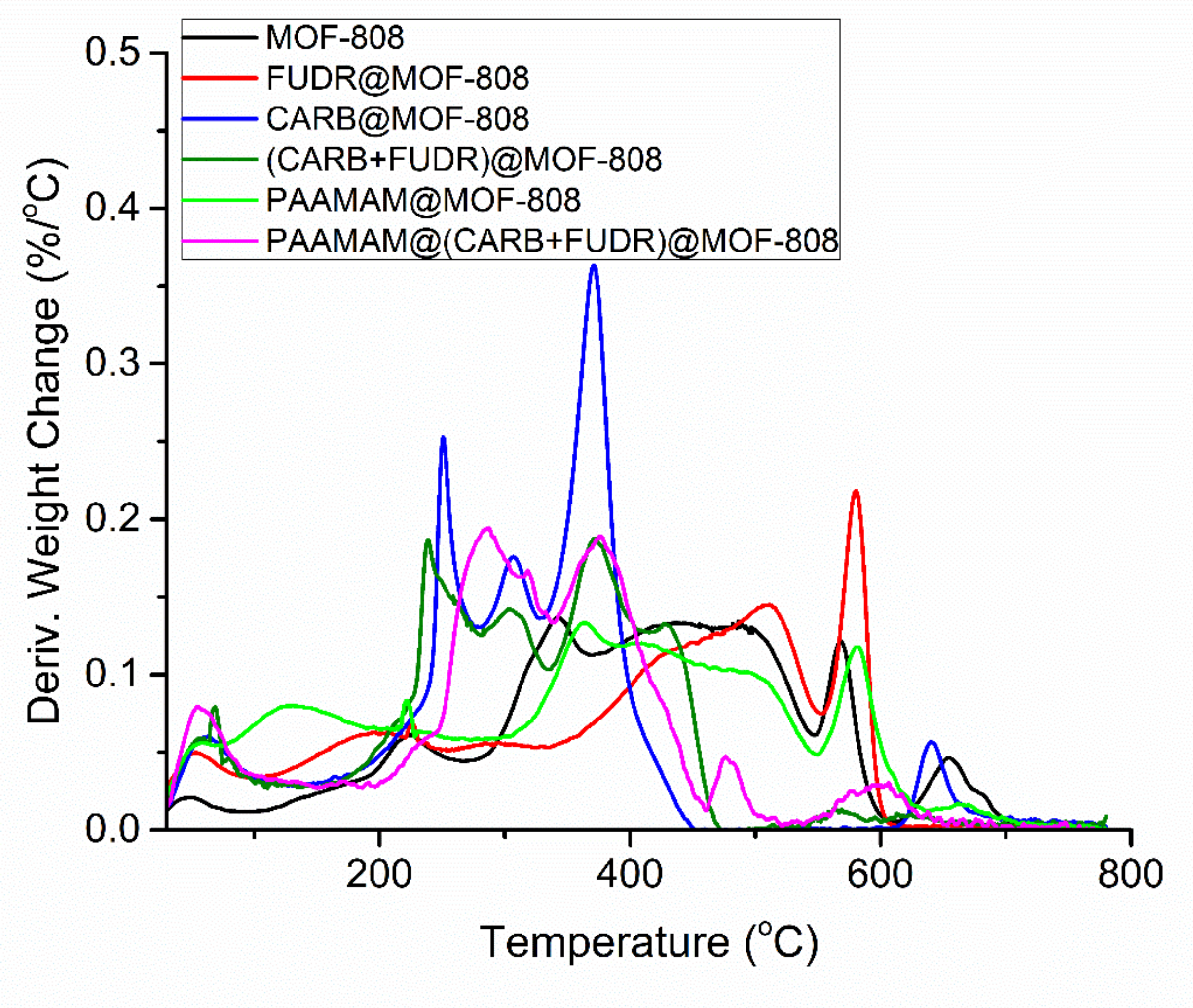

Figure S20. Derivative weight change (%/°C) vs temperature for MOF-808 compared to its drug and glycopolymer functionalised structures.

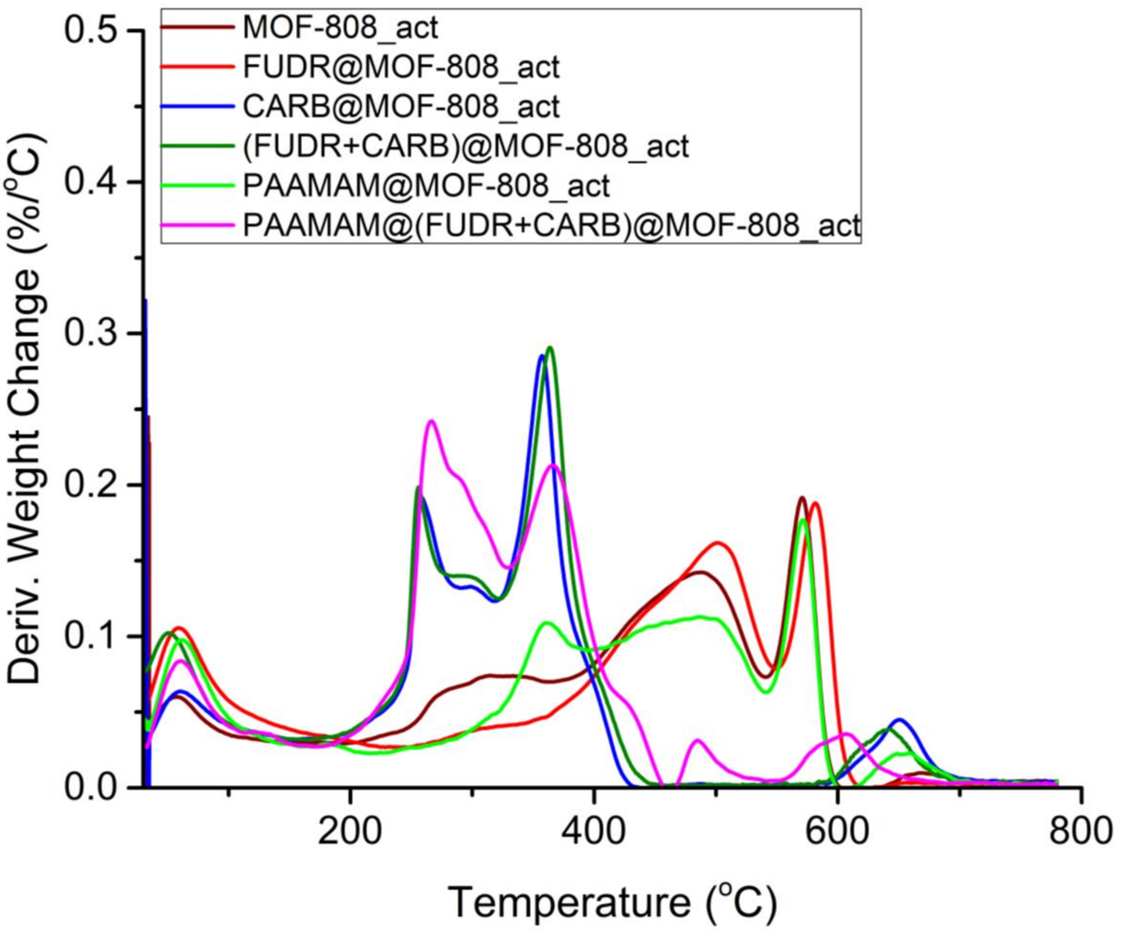

Figure S21. Derivative weight change (%/°C) vs temperature for MOF-808_act compared to its drug and glycopolymer functionalised structures.

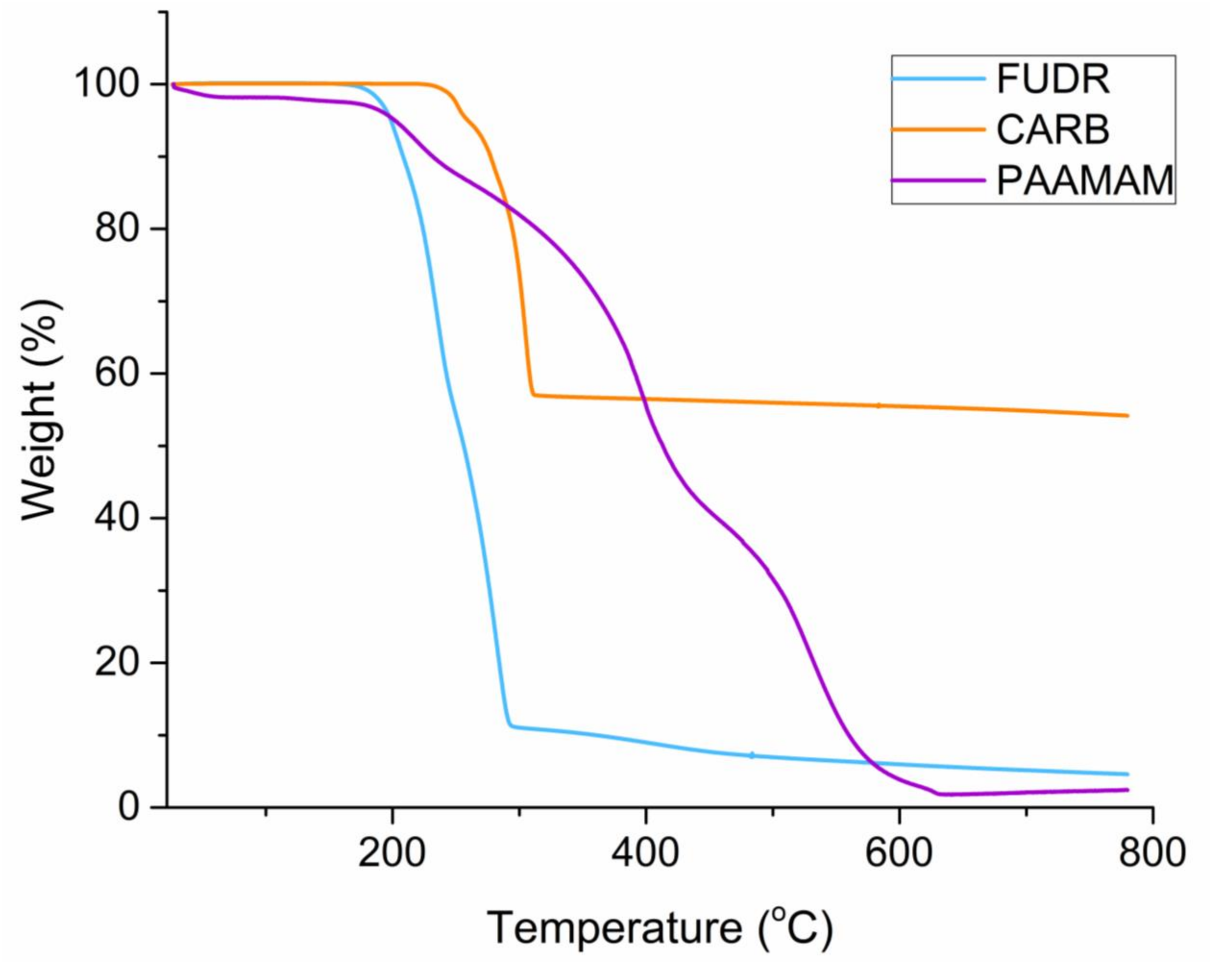

Figure S22. TGA traces of the chemotherapy drugs FUDR and CARB, and the glycopolymer, PAAMAM, represented as weight loss (%) vs temperature.

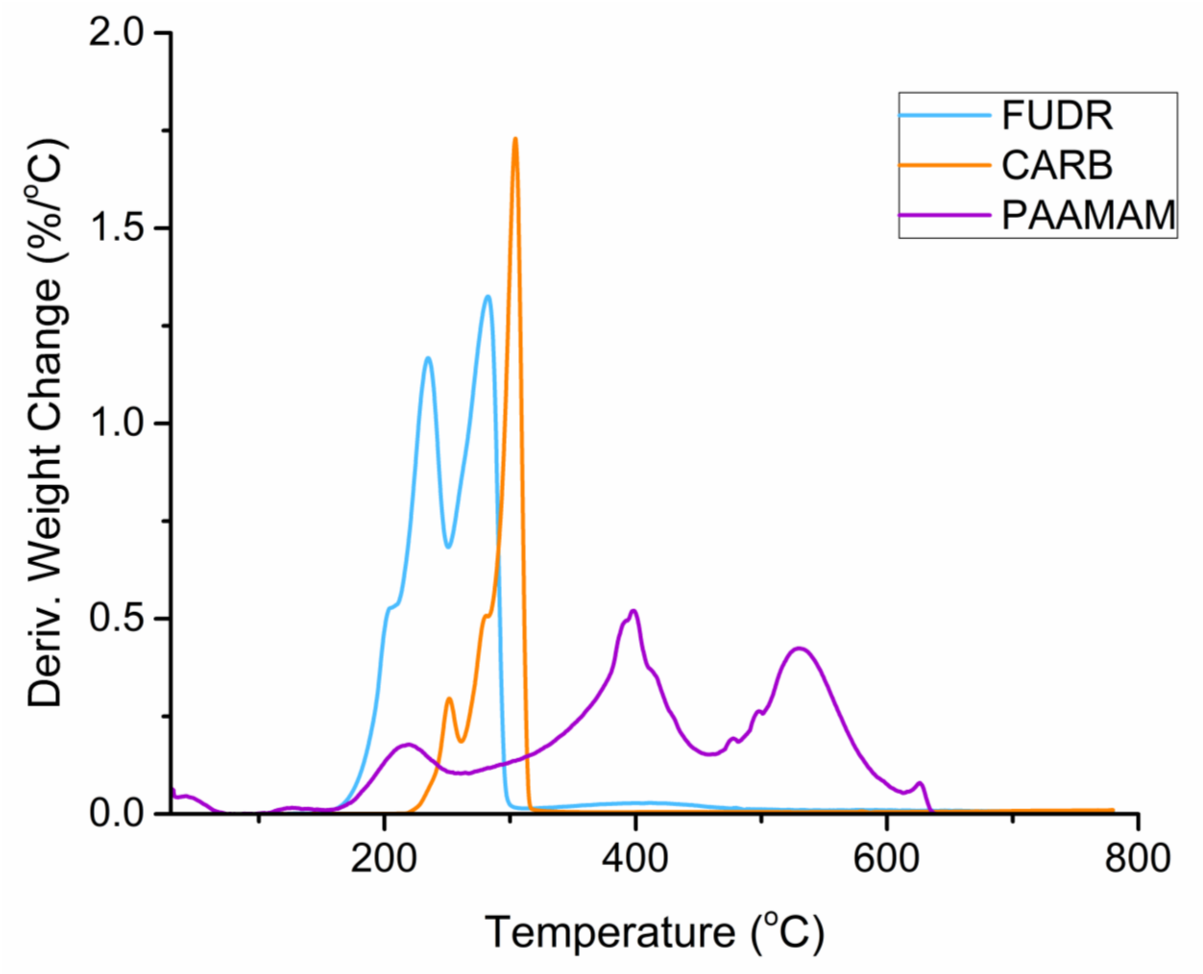

Figure S23. TGA traces of the chemotherapy drugs FUDR and CARB, and the glycopolymer, PAAMAM, represented as derivative weight change (%/°C) vs temperature. Derived from Figure S11.

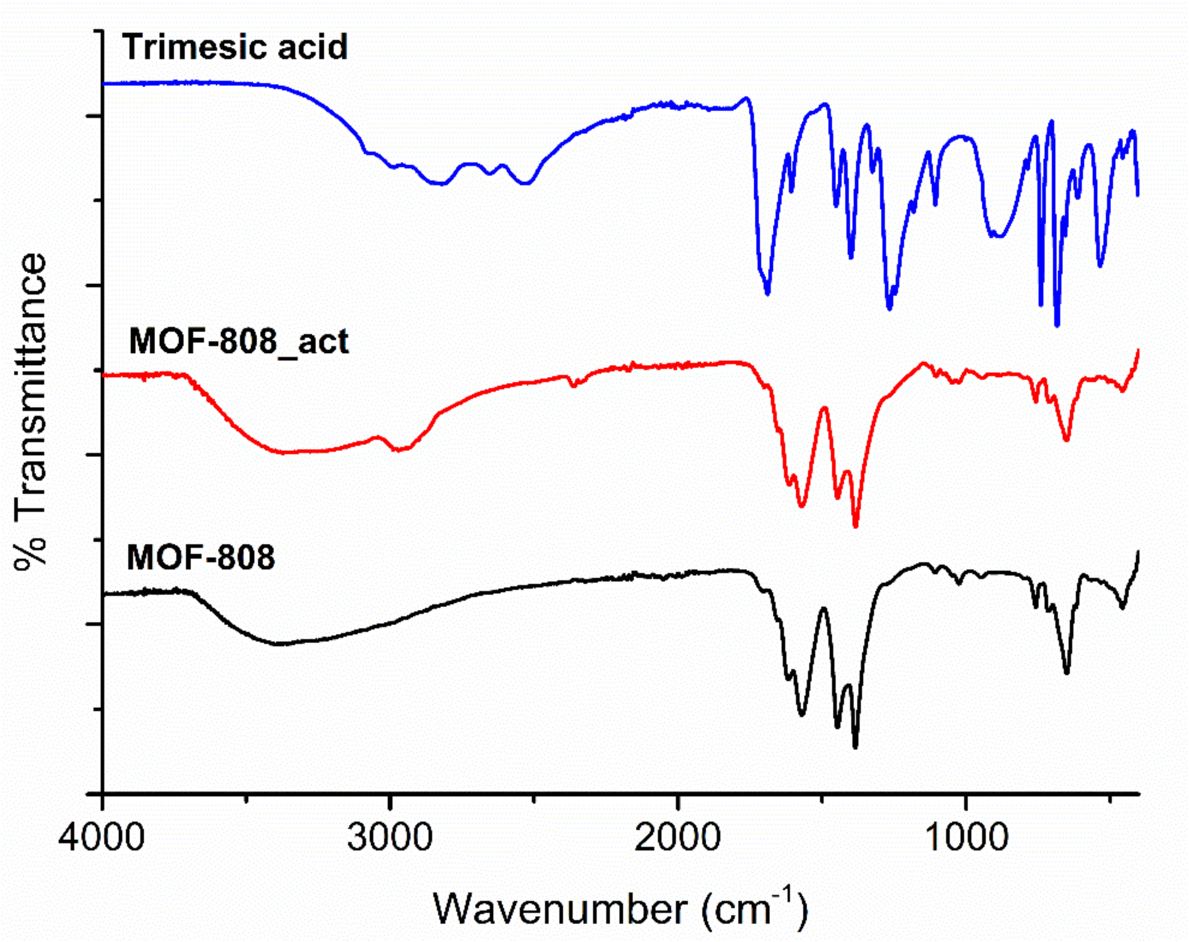

Figure S24. FTIR spectra of MOF-808 and MOF-808_act in comparison to the linker, trimesic acid.

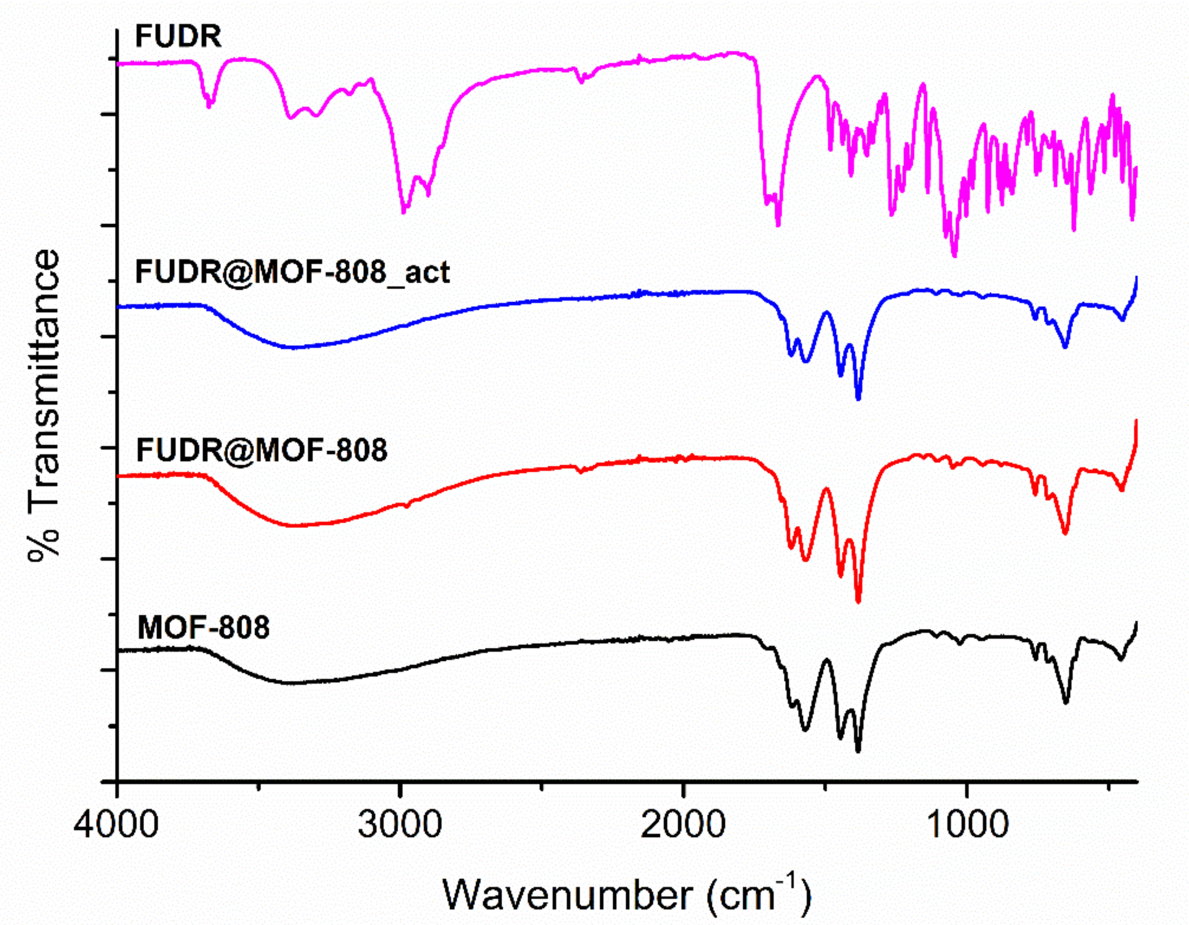

Figure S25. FTIR spectra of MOF-808, FUDR@MOF-808, FUDR@MOF-808_act and FUDR.

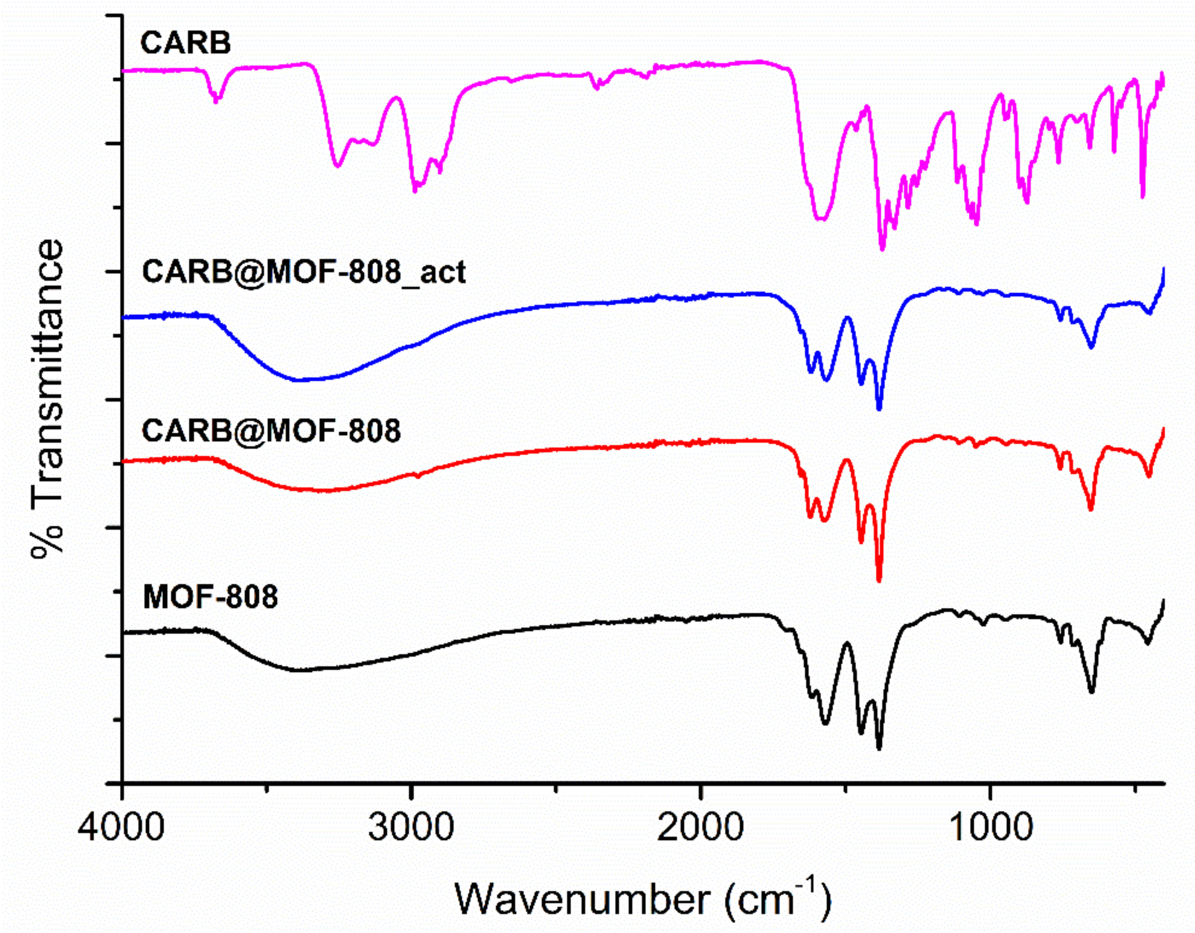

Figure S26. FTIR spectra of MOF-808, CARB@MOF-808, CARB@MOF-808_act and CARB.

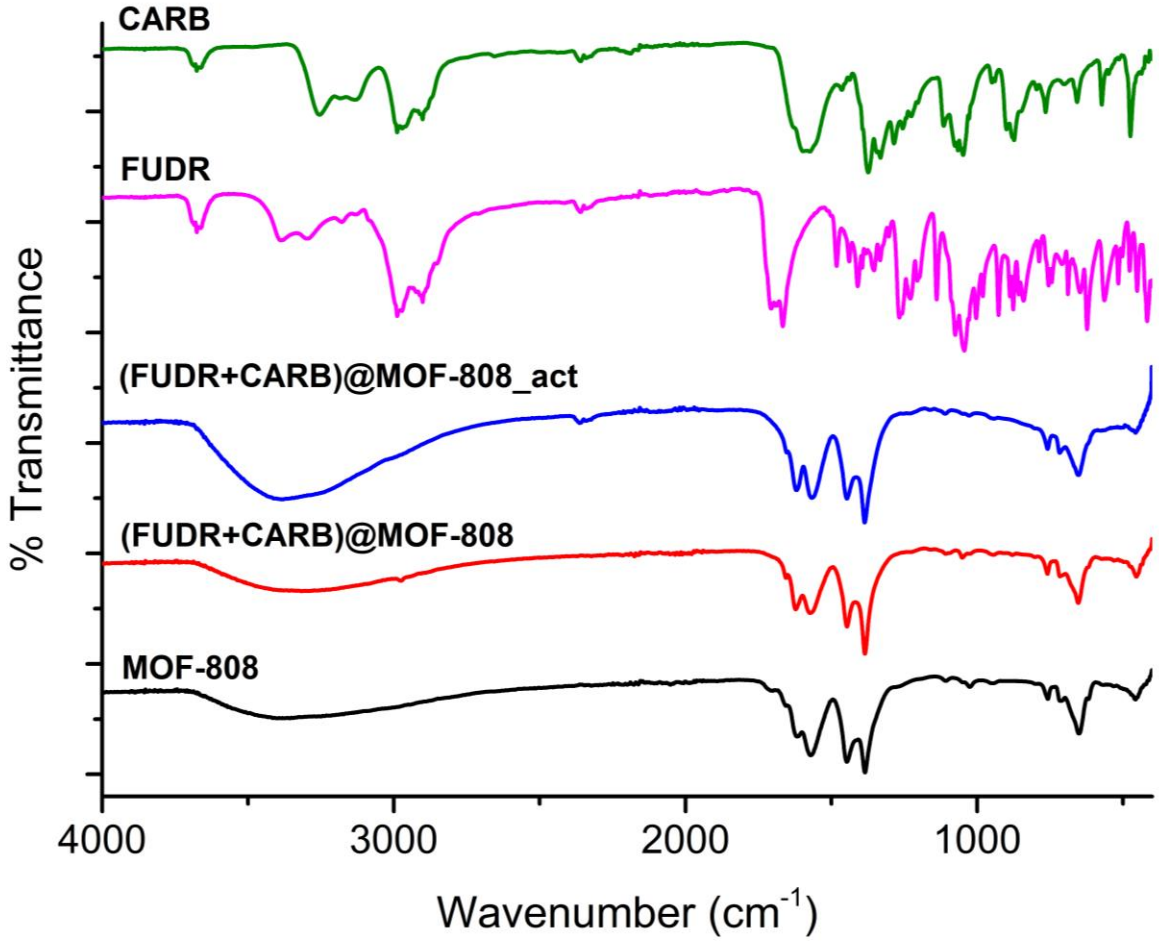

Figure S27. FTIR spectra of MOF-808, (FUDR+CARB)@MOF-808, (FUDR+CARB)@MOF-808_act as well as FUDR and CARB for comparison.

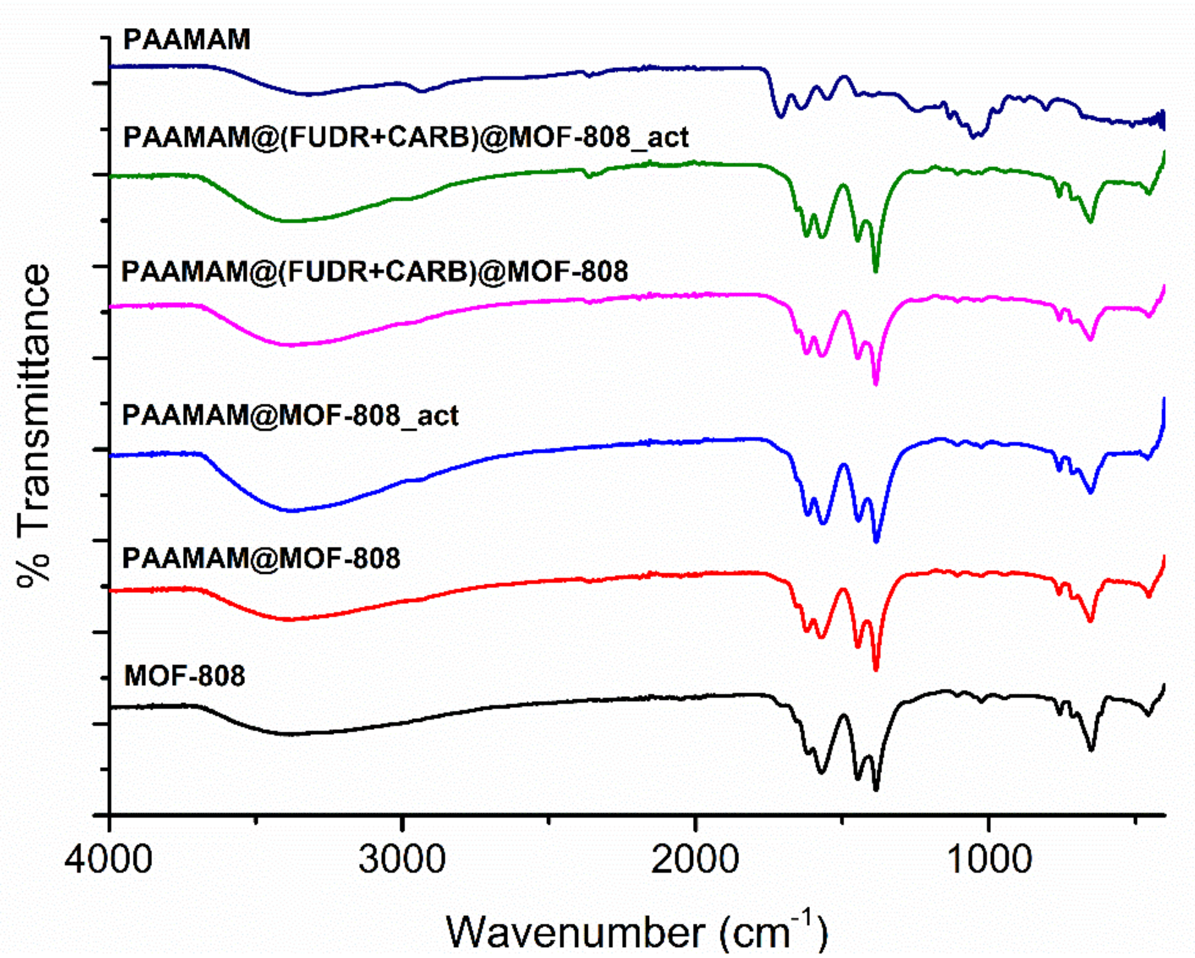

Figure S28. FTIR spectra of MOF-808, PAAMAM@MOF-808, PAAMAM@MOF-808_act, PAAMAM@(FUDR+CARB)@MOF-808, PAAMAM@(FUDR+CARB)@MOF-808_act and PAAMAM.

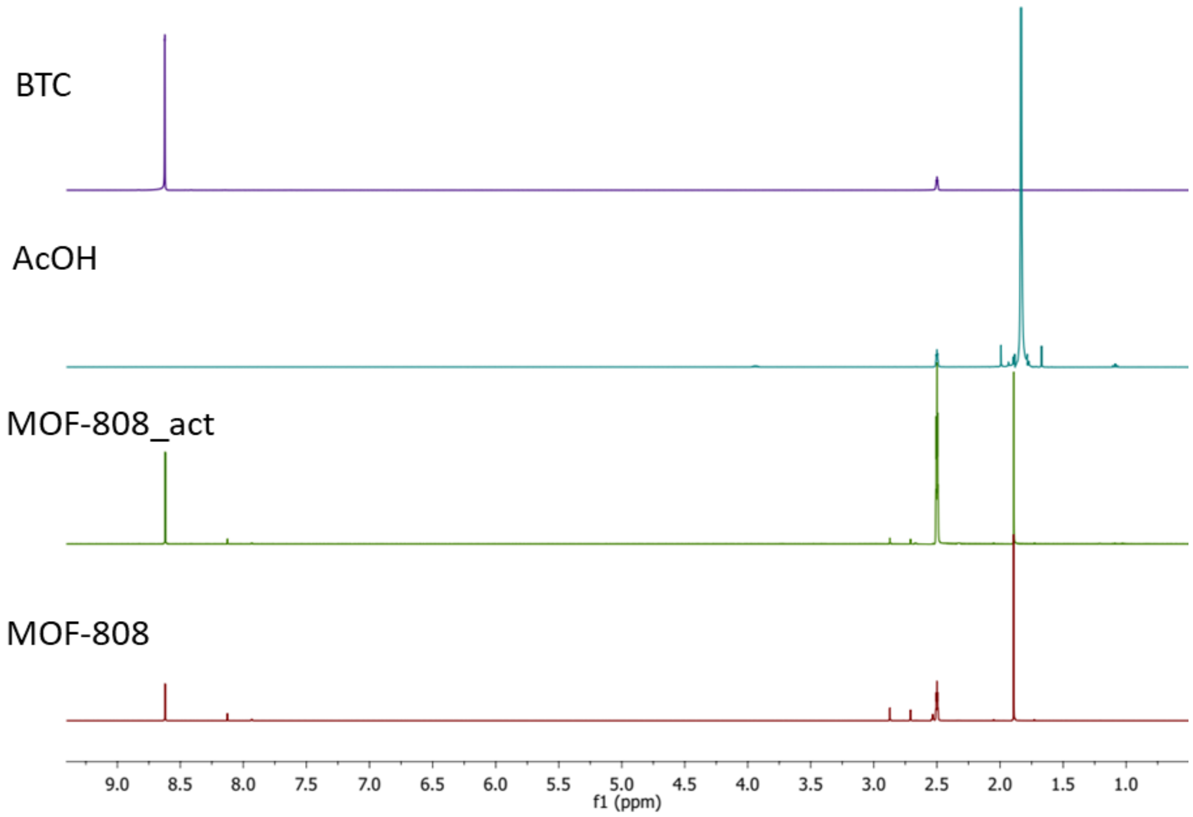

Figure S29. ^{1}H NMR spectrum (D_2SO_4 / DMSO-d_6) of digested MOF-808 and MOF-808_act as well as acetic acid (AcOH) and benzene-1,3,5-tricarboxylic acid (BTC) for comparison.

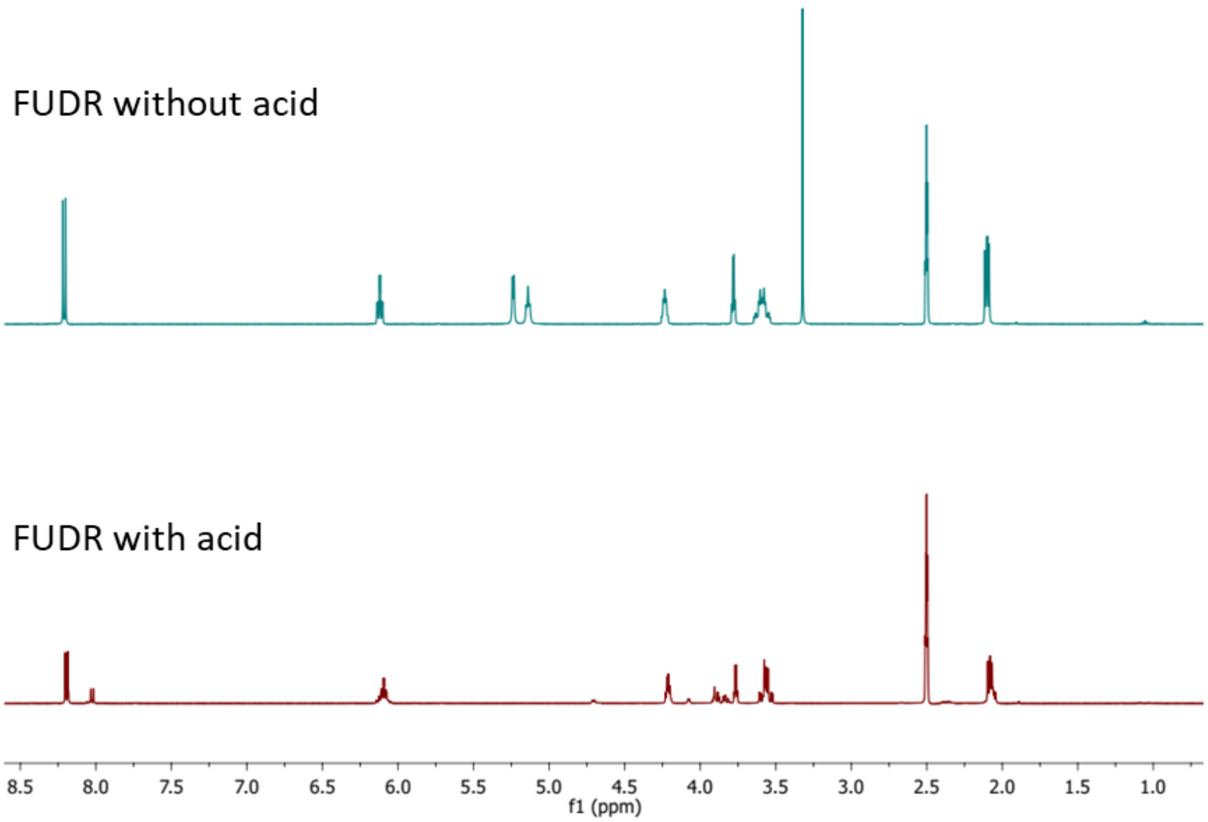

Figure S30. ^{1}H NMR spectrum (D_2SO_4 / DMSO-d_6) of digested FUDR in comparison to that of FUDR without D_2SO_4 acid treatment in DMSO-d_6.

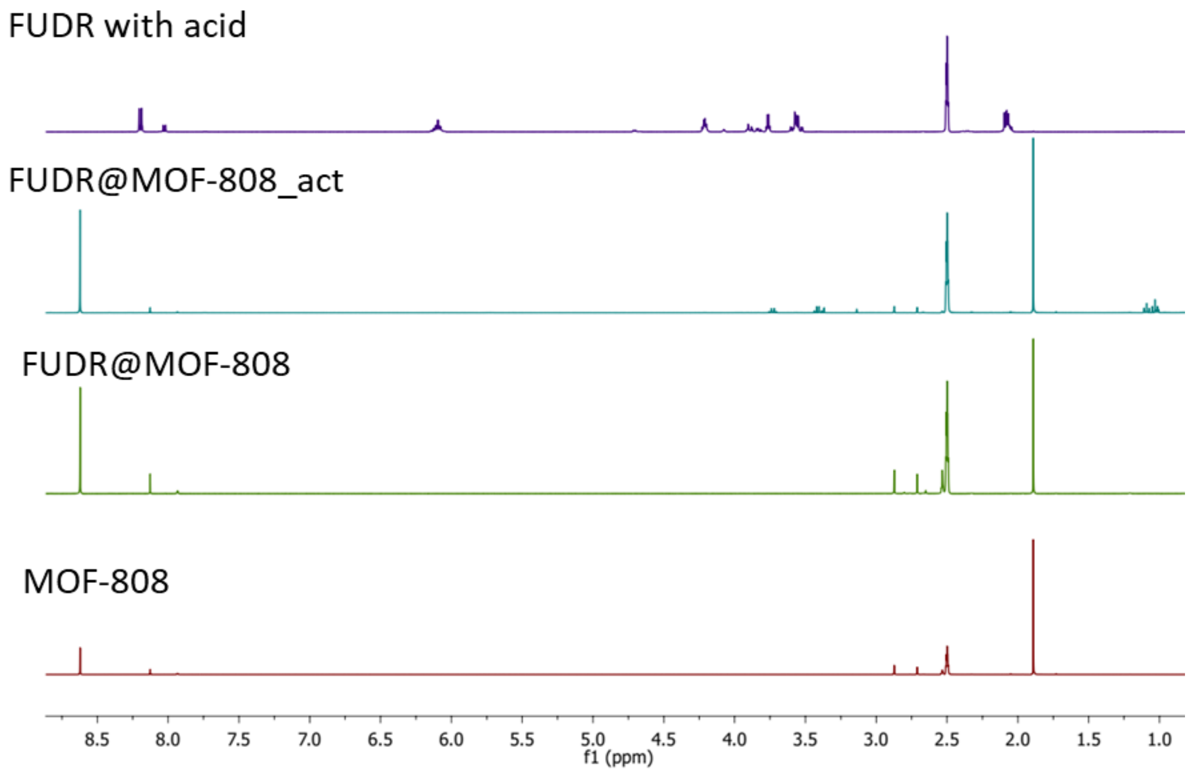

Figure S31. ^{1}H NMR spectrum (D_2SO_4 / DMSO-d_6) of digested MOF-808, FUDR@MOF-808, FUDR@MOF-808_act and FUDR.

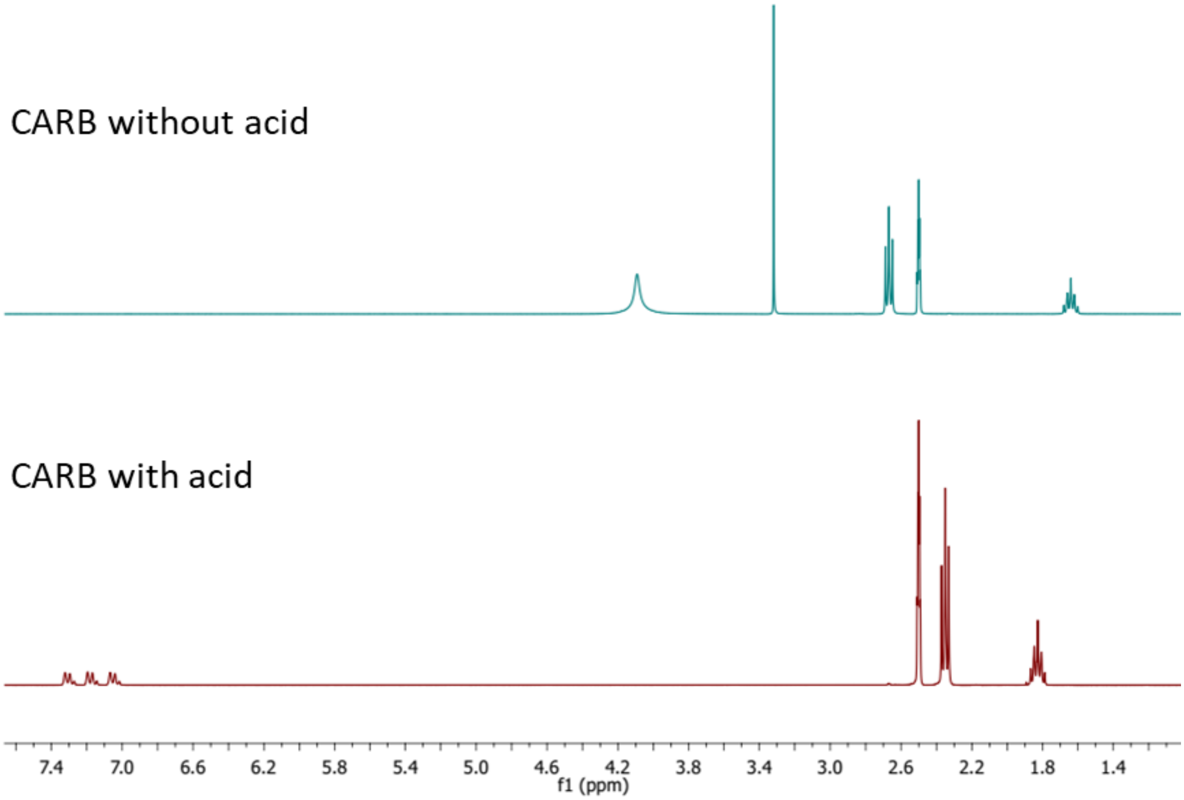

Figure S32. ^{1}H NMR spectrum (D_2SO_4 / DMSO-d_6) of digested CARB in comparison to that for CARB without D_2SO_4 acid treatment in DMSO-d_6.

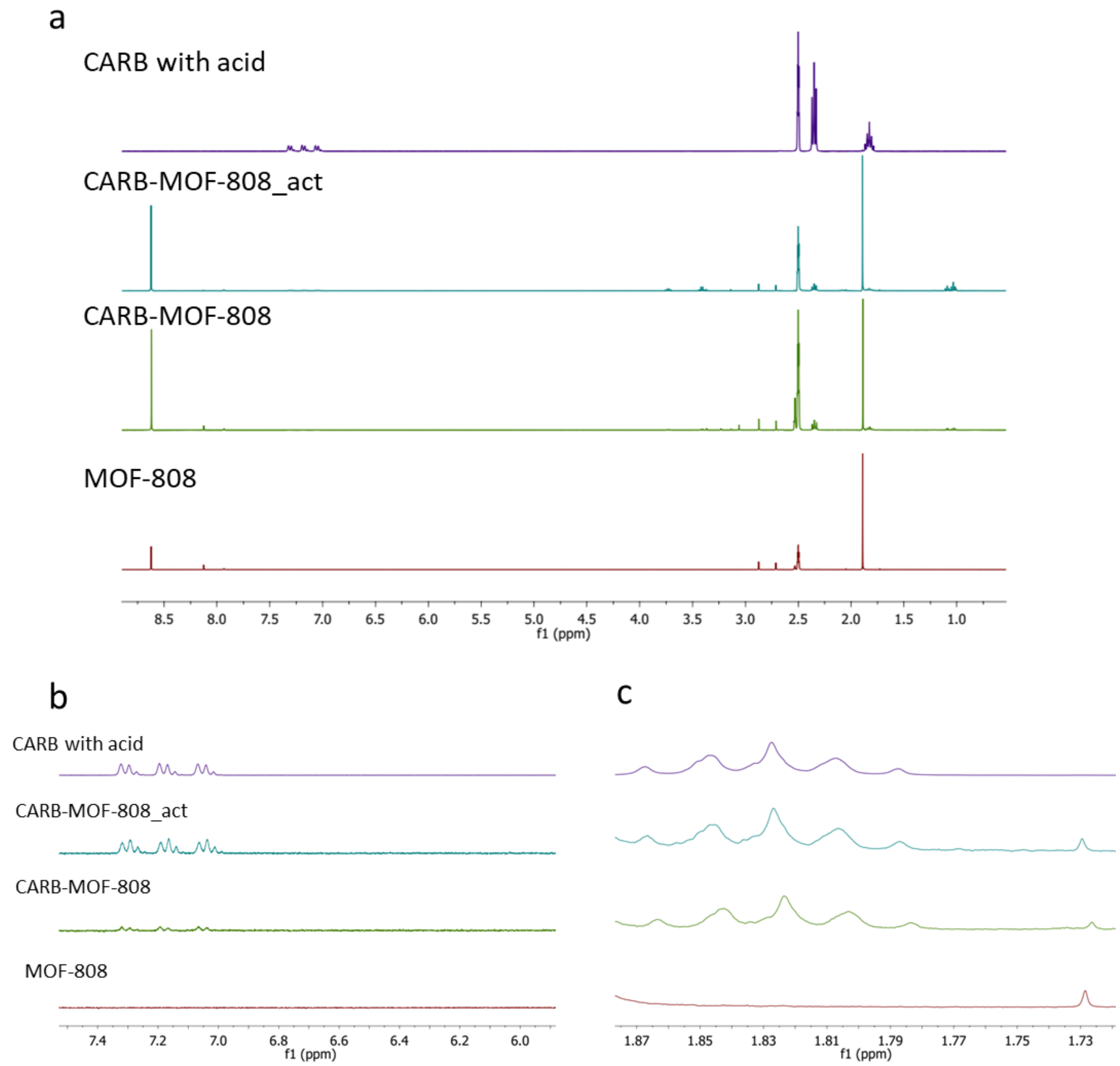

Figure S33. ^{1}H NMR spectrum (D_2SO_4 / DMSO-d_6) of (a) digested MOF-808, CARB@MOF-808, CARB@MOF-808_act and CARB, and (b) partial ^{1}H NMR spectrum around 7.4 – 6.0 ppm and (c) around 1.87 – 1.73 ppm.

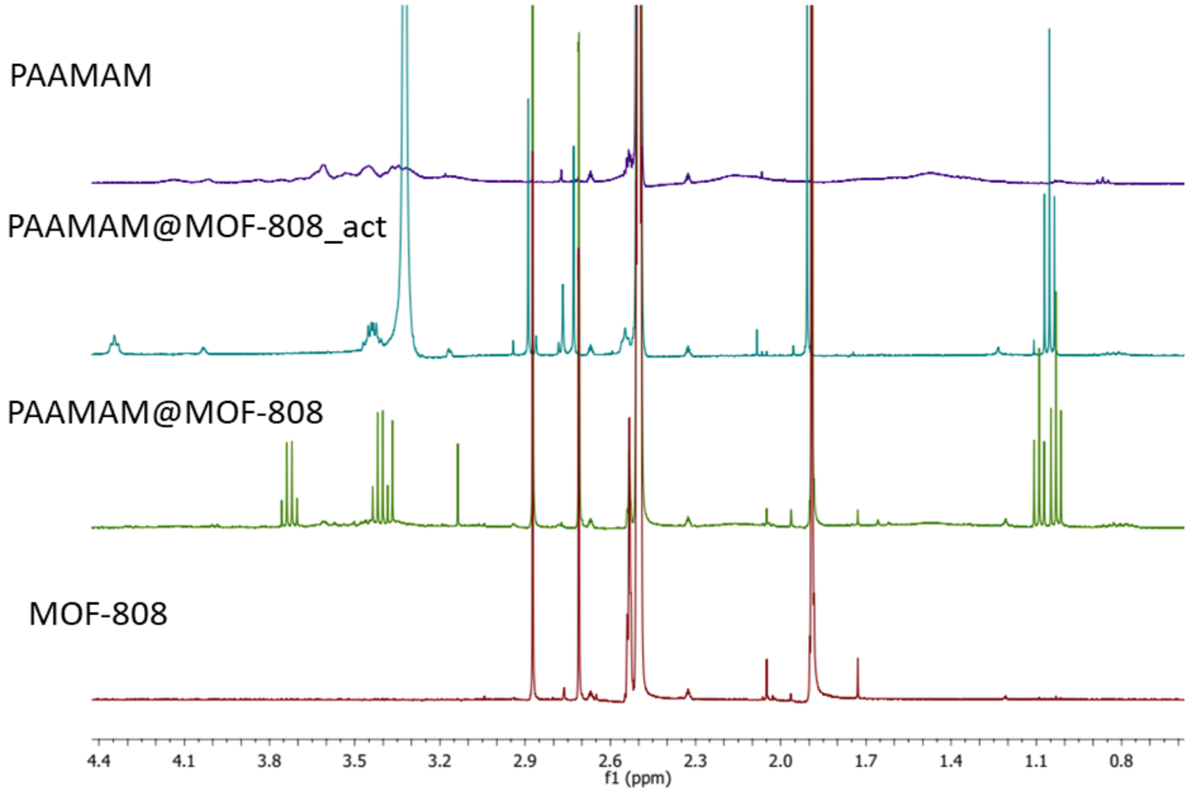

Figure S34. ^{1}H NMR spectrum (D_2SO_4 / DMSO-d_6) of MOF-808, PAAMAM@MOF-808, PAAMAM@MOF-808_act and PAAMAM.

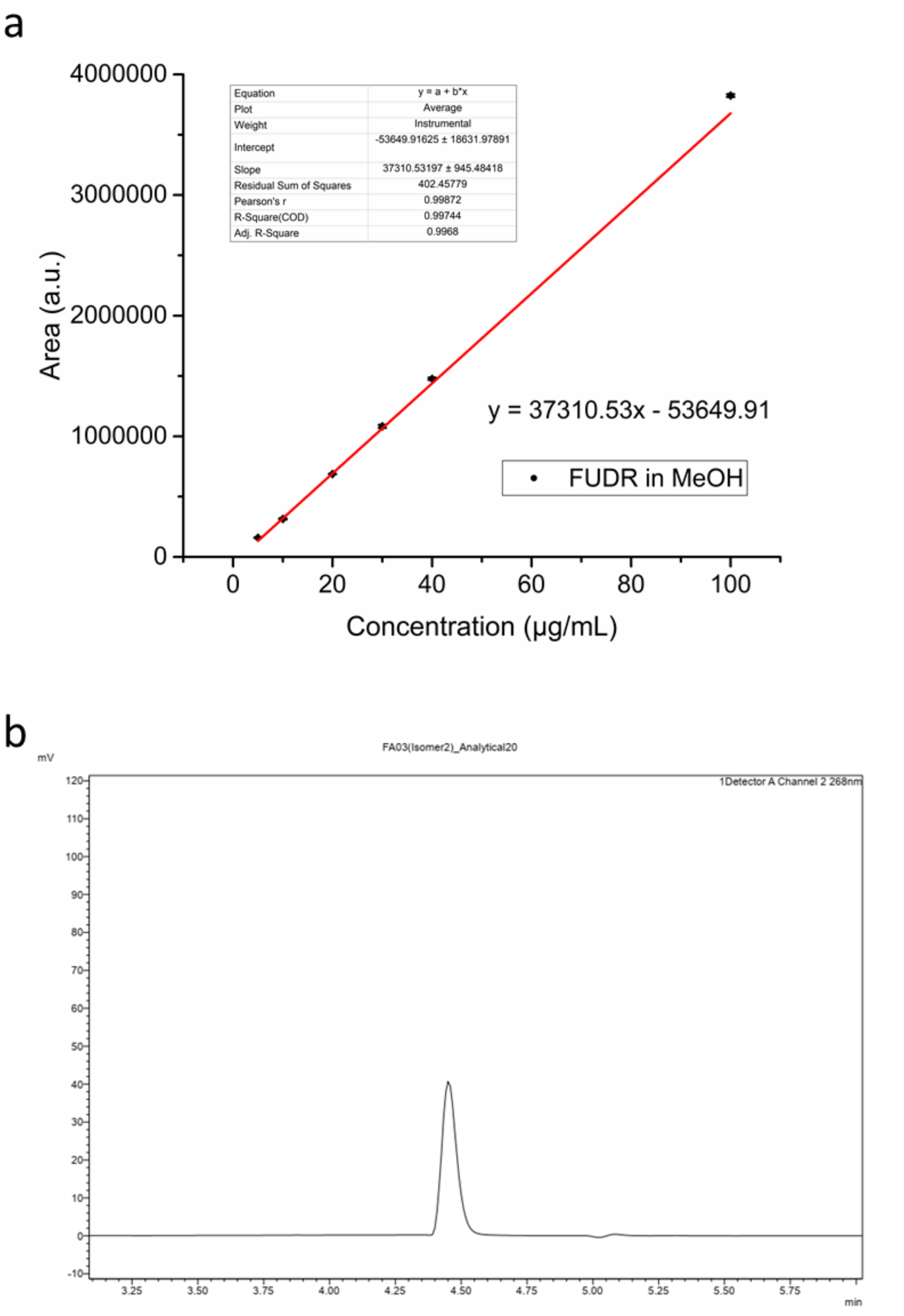

Figure S35. FUDR determination by HPLC (a) Calibration curve of FUDR prepared using RT-HPLC at λ = 268 nm in methanol (n = 3), r = 0.99872; (b) FUDR retention time in methanol (5 µg mL^{-1} FUDR).

S5. *In Vitro* Cytotoxicity

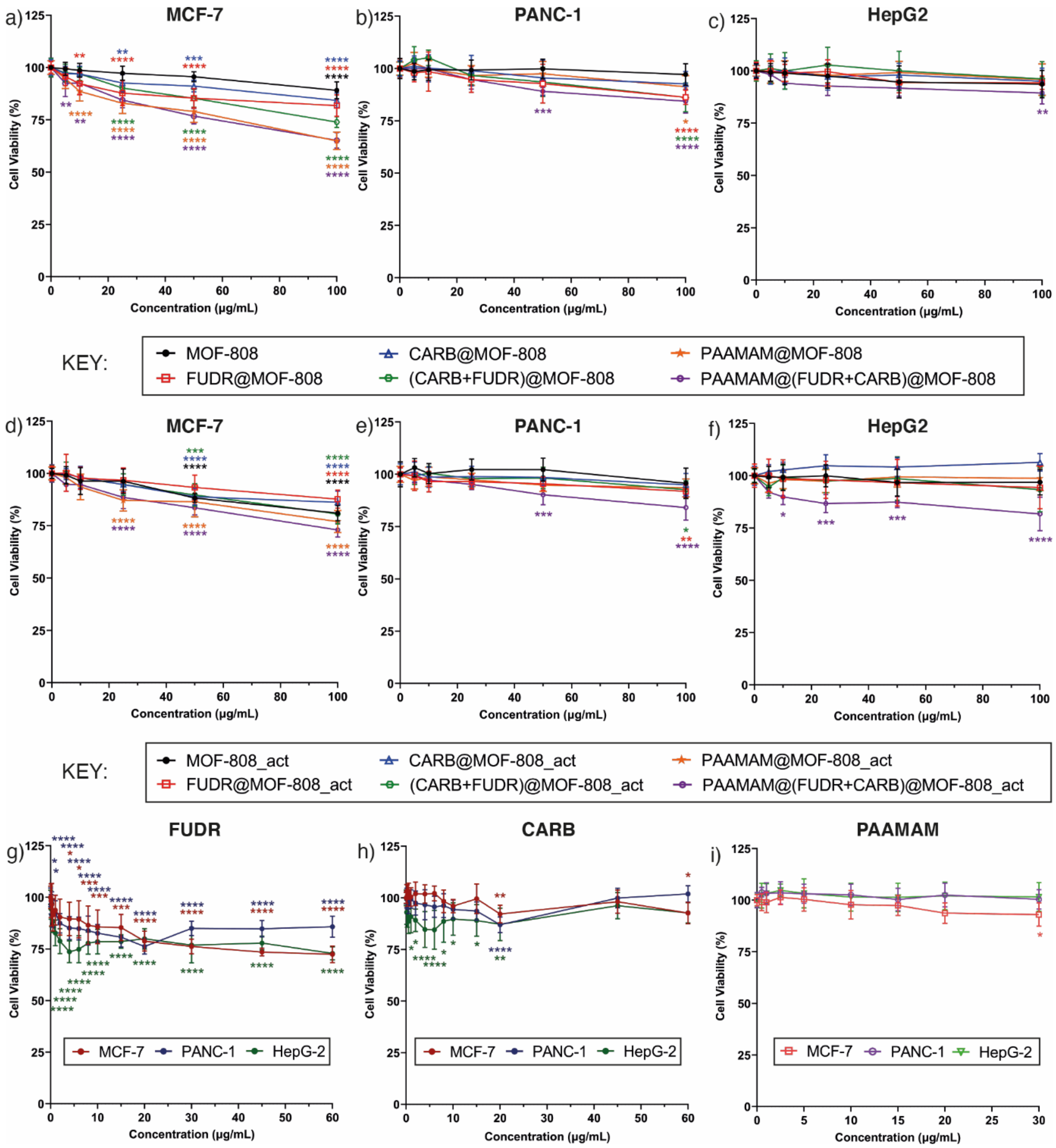

Figure S36. Cell viabilities of a) MCF-7, b) PANC-1 and c) HepG2 cells treated with MOF-808 and its drug loaded and PAAMAM coated formulations, compared to cell viabilities of d) MCF-7, e) PANC-1 and f) HepG2 cells treated with MOF-808_act and its drug loaded and PAAMAM coated formulations. Cell viabilities when treated with g) free FUDR, h) free CARB, and i) PAAMAM are also provided for comparison. All incubations were carried out for 24 h. Viabilities were assessed by the Alamar blue cell viability assay with untreated cells accepted as control. The data were expressed as mean and standard deviation (n = 3×3), $p \leq 0.05$ (*), $p \leq 0.01$ (**), $p \leq 0.001$ (***) and $p \leq 0.0001$ (****). The stars show the statistical significance of each data point from the control.

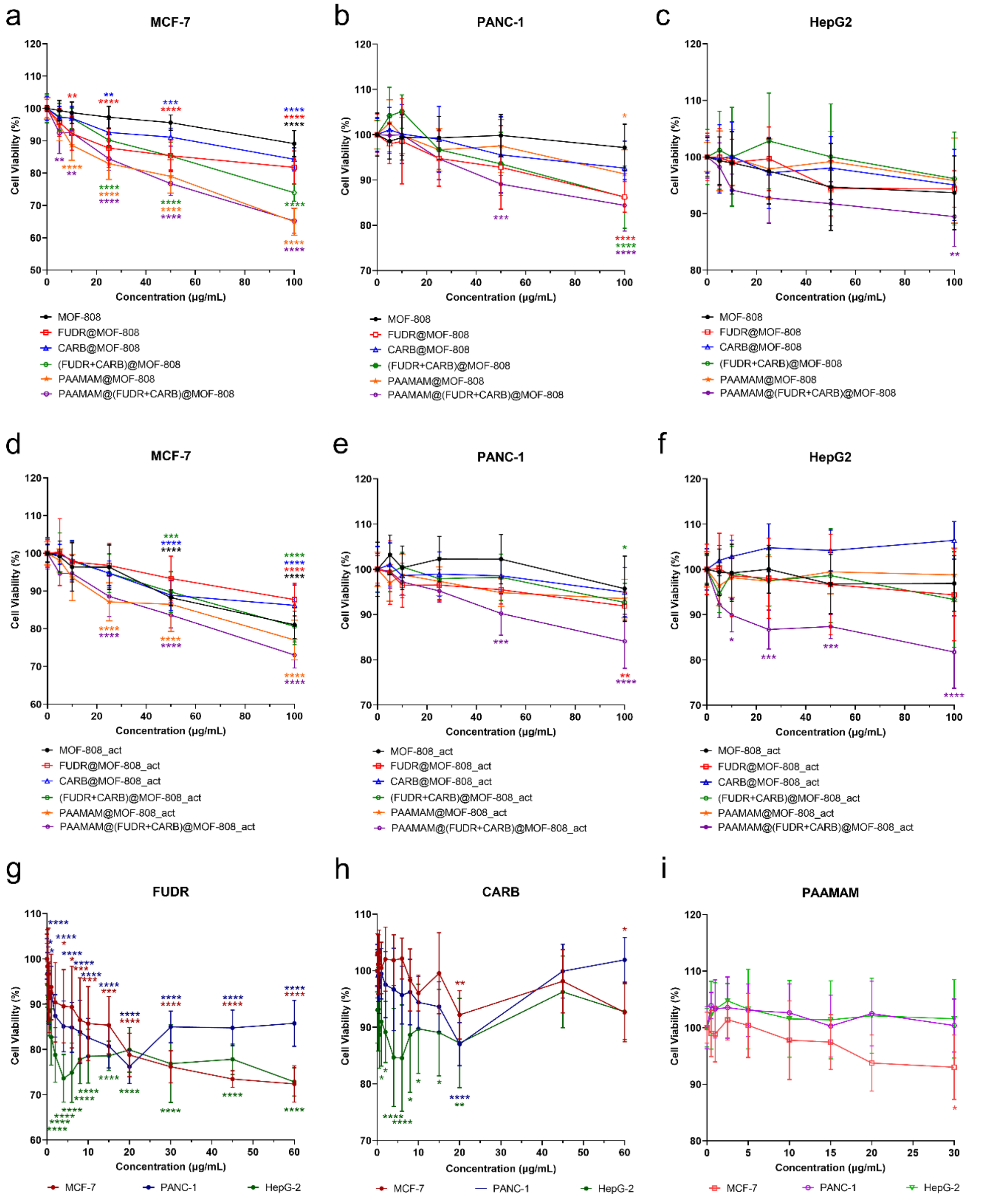

Figure S37. Cell viability of MCF-7, PANC-1 and HepG2 cells treated with MOF-808 and MOF-808_act and their drug loaded and PAAMAM coated formulations, and free FUDR, CARB and PAAMAM for 24 h, performed by Alamar blue cell viability assay. Data are taken from Figure S36 but rescaled on the y axis to better distinguish differences between samples. Cell viabilities of a) MCF-7, b) PANC-1 and c) HepG2 cells treated with MOF-808 and its drug loaded and PAAMAM coated formulations, compared to cell viabilities of d) MCF-7, e) PANC-1 and f) HepG2 cells treated with MOF-808_act and its drug loaded and PAAMAM coated formulations. Cell viabilities of the three cell lines when treated

with g) free FUDR, h) free CARB, and i) PAAMAM. Untreated cells were accepted as control. The data were expressed as mean and standard deviation (n = 3×3), $p \leq 0.05$ (*), $p \leq 0.01$ (**), $p \leq 0.001$ (***) and $p \leq 0.0001$ (****). The stars show the statistical significance of each data point from the control.

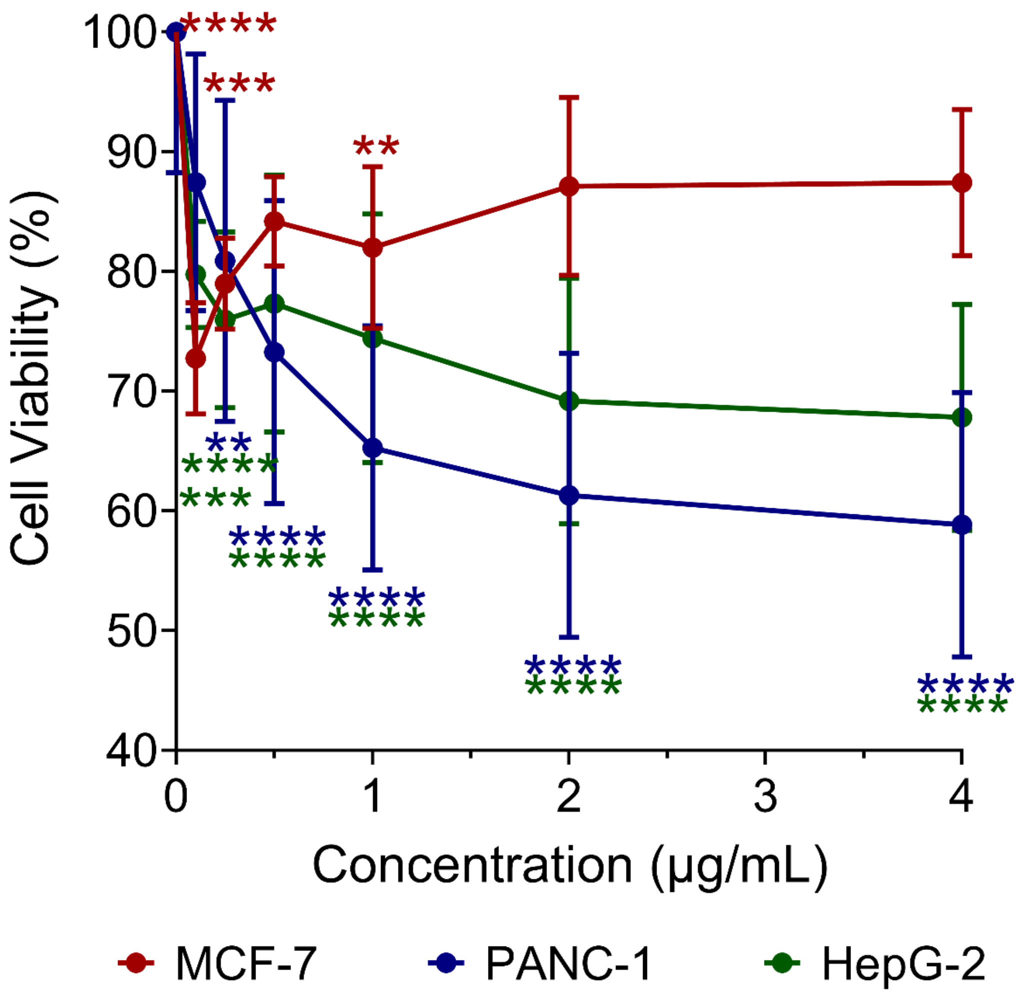

Figure S38. Cell viability of MCF-7, PANC-1, and HepG2 cells against low concentrations of FUDR after 72 h incubation. Data are reproduced from Figure 4g to show statistics in the low concentration regime. Viabilities assessed by the Alamar blue cell viability assay with untreated cells accepted as control. The data were expressed as mean and standard deviation (n = 3×3), $p \leq 0.05$ (*), $p \leq 0.01$ (**), $p \leq 0.001$ (***) and $p \leq 0.0001$ (****). The stars show the statistical significance of each data point from the control.

Table S1. Equivalent FUDR and CARB concentrations to nanoparticle concentrations used in 72 h cytotoxicity study (Figure 4).

	Cytotoxicity Studies Concentrations (µg mL^{-1})						
**Samples**	**Nanoparticle concentration (µg mL^{-1})**	**0**	**5**	**10**	**25**	**50**	**100**
FUDR@MOF-808	Eq. FUDR concentration (µg mL^{-1})	0	0.059	0.118	0.295	0.59	1.18
CARB@MOF-808	Eq. CARB concentration (µg mL^{-1})	0	0.5495	1.099	2.7475	5.495	10.99
(FUDR+CARB)@MOF-808	Eq. FUDR concentration (µg mL^{-1})	0	0.0295	0.059	0.1475	0.295	0.59
	Eq. CARB concentration (µg mL^{-1})	0	0.46	0.92	2.3	4.6	9.2
PAAMAM@(FUDR+CARB)@MOF-808	Eq. FUDR concentration (µg mL^{-1})	0	0.023	0.046	0.115	0.23	0.46
	Eq. CARB concentration (µg mL^{-1})	0	0.413	0.826	2.065	4.13	8.26
FUDR@MOF-808_act	Eq. FUDR concentration (µg mL^{-1})	0	0.0645	0.129	0.3225	0.645	1.29
CARB@MOF-808_act	Eq. CARB concentration (µg mL^{-1})	0	0.687	1.374	3.435	6.87	13.74
(FUDR+CARB)@MOF-808_act	Eq. FUDR concentration (µg mL^{-1})	0	0.0315	0.063	0.1575	0.315	0.63
	Eq. CARB concentration (µg mL^{-1})	0	0.607	1.214	3.035	6.07	12.14
PAAMAM@(FUDR+CARB)@MOF-808_act	Eq. FUDR concentration (µg mL^{-1})	0	0.0345	0.069	0.1725	0.345	0.69
	Eq. CARB concentration (µg mL^{-1})	0	0.4795	0.959	2.3975	4.795	9.59

S6. Flow Cytometry

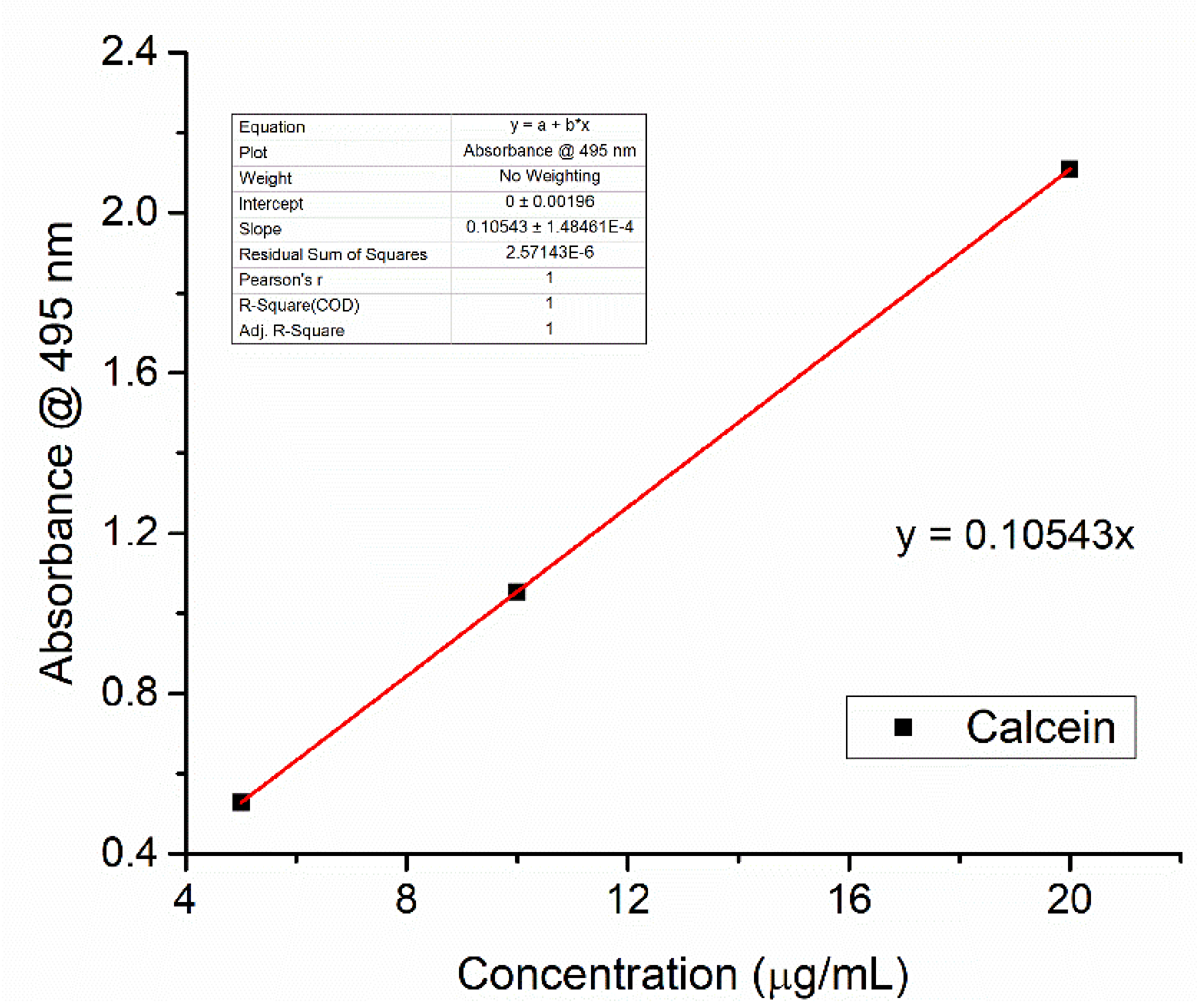

Figure S39. Calibration curve for calcein in PBS (1X, pH 7.4) at 495 nm. r = 1.

Table S2. % Calcein loading (*w/w*) in calcein loaded samples measured in degradation solutions of calcein loaded samples using the calibration curve for calcein in Figure S23.

**Samples**	***w/w* % calcein**
MOF-808	8.7
PAAMAM@MOF-808	1.9
(CARB+FUDR)@MOF-808	12.0
PAAMAM@(CARB+FUDR)@MOF-808	3.3
MOF-808_act	5.5
PAAMAM@MOF-808_act	2.3
(CARB+FUDR)@MOF-808_act	12.3
PAAMAM@(CARB+FUDR)@MOF-808_act	4.0
